# Pharmacological potential of Chinese botanical drugs in managing chronic kidney disease by targeting mitochondrial quality control

**DOI:** 10.3389/fphar.2025.1725842

**Published:** 2026-02-19

**Authors:** Hongyu Liu, Shumin Huang, Shichun Chen, Shuzhen Liang, Minying Huang, Shiyu Li, Yongxiang Xu, Baocheng Xie

**Affiliations:** 1 Department of Pharmacy, Central Hospital of Guangdong Prison, Guangzhou, Guangdong, China; 2 The Tenth Affiliated Hospital, Southern Medical University (Dongguan People’s Hospital), Dongguan, Guangdong, China; 3 Department of Pharmacy, The Tenth Affiliated Hospital, Southern Medical University (Dongguan People’s Hospital), Dongguan, Guangdong, China; 4 Department of Pharmacy, The Third Affiliated Hospital of Guangzhou Medical University, Guangzhou, Guangdong, China; 5 Rehabilitation Department, Central Hospital of Dalian University of Technology, Dalian, Liaoning, China

**Keywords:** Chinese botanical drugs, chronic kidney disease, gut-kidney axis, mitochondrial dysfunction, mitochondrial quality control

## Abstract

Chronic kidney disease (CKD) is a multifactorial health issue characterized by structural and functional impairments of the kidneys, with significant incidence and mortality rates in global populations. Mitochondrial quality control (MQC) comprises cellular mechanisms that maintain mitochondrial health, and imbalances in the MQC system, including abnormalities in mitochondrial oxidative stress, dynamics, biogenesis, autophagy, and apoptosis, have been implicated in the onset and progression of CKD. In addition, the interplay between gut microbiota, microbial metabolites, and mitochondrial integrity has gained increasing attention in CKD research. Consequently, therapeutic strategies targeting MQC have attracted considerable research interest. Chinese botanical drugs (CBD), known for their multi-component, multi-target profiles and favorable safety, demonstrate considerable potential in slowing CKD progression by modulating MQC. This review systematically summarizes current evidence on CBD metabolites and formulations that ameliorate CKD through MQC regulation. Firstly, we outline the mechanisms of action of MQC system, with a focus on its role in CKD. We then discussed the pivotal role of the gut microbiota-microbial metabolites-mitochondria axis in the progression of CKD. Finally, we provide a summary of CBD metabolites and formulations that target the MQC system for CKD treatment to date, and explore their specific therapeutic mechanisms. Despite promising preclinical findings, we also critically assess limitations within the available literature, such as methodological variability and a lack of clinical validation. By integrating current knowledge and identifying key research gaps, this review aims to inform future studies and advance the development of CBD-based therapies for CKD.

## 1 Introduction

Chronic kidney disease (CKD) is a complex clinical syndrome entailing progressive structural and functional impairment of the kidneys, commonly arising from conditions such as diabetic kidney disease (DKD) and glomerulonephritis. With a global prevalence of 14.3% and considerable associated mortality, CKD poses a substantial public health challenge (Barrera-Chimal et al., 2019). Pathologically, CKD presents with glomerular hypertrophy, mesangial widening, podocyte damage, and even glomerulosclerosis and interstitial fibrosis (Bi et al., 2024; Humphreys, 2018). CKD is classified into five stages based on the glomerular filtration rate (GFR). Early detection and intervention can reduce complications and improve quality of life. However, late-stage CKD may progress to end-stage renal failure and uremia, which is accompanied by multi-system symptoms such as cardiovascular, gastrointestinal, and respiratory disorders, as well as metabolic dysfunction, necessitating timely renal replacement therapy. Current management relies on conventional treatments, such as corticosteroids, immunosuppressants, and biologics. However, these agents are associated with significant adverse reactions (e.g., electrolyte imbalances, hepatorenal damage, increased infection risk) and exhibit variable individual responses, which complicates the determination of optimal dosage and treatment regimens (Agarwal et al., 2022; Gohda and Murakoshi, 2022; Khoo et al., 2021). Furthermore, specific therapeutics for CKD remain scarce.

Mitochondria, double-membrane organelles known as “cellular powerhouses,” generate adenosine triphosphate (ATP) through oxidative phosphorylation (OXPHOS) and are highly abundant in the kidney (Kummer and Ban, 2021). Renal function relies on mitochondrial biogenesis, fusion, and fission within intrinsic cells to adapt to metabolic changes. Mitochondrial dysfunction is common in kidney diseases of diverse etiologies (e.g., diabetes mellitus [DM], hypertension [HTN]), inducing oxidative stress (OS), autophagy, excessive fission, fusion defects, and apoptosis. This ultimately leads to cellular energy depletion and triggers pathological alterations in cellular function and structure. Shah et al. (2024) confirmed a positive correlation between mitochondrial dysfunction and AKI-to-CKD transition, making the maintenance of mitochondrial dynamic homeostasis a key factor in protecting renal cells. To preserve mitochondrial integrity, mitochondrial quality control (MQC), a network including OS neutralization, dynamics regulation, mitophagy (a selective form of autophagy that removes damaged mitochondria), biogenesis, and apoptosis (Roca-Portoles and Tait, 2021), collectively sustains mitochondrial health (Picca et al., 2018). Loss of MQC causes mitochondrial damage and organ failure (Bhargava and Schnellmann, 2017; Suliman and Piantadosi, 2016), and growing evidence links MQC disorders to CKD pathogenesis (Bhatia et al., 2019; Tang et al., 2021), making MQC-targeted interventions promising for renal protection. In recent years, the interplay between gut microbiota alterations, microbial metabolites, and mitochondrial dysfunction in CKD has emerged as a research hotspot (Tao et al., 2024). Within the pathophysiological mechanisms of CKD development, the role of the gut microbiota-microbial metabolites-mitochondria axis has become increasingly evident. Targeting this axis represents a novel approach for both preventing and treating CKD.

Chinese botanical drugs (CBD) contain abundant natural bioactive metabolites, many rich in phytochemicals, including polyphenols, flavonoids, saponins, and alkaloids, which have been extensively studied in managing CKD. Based on the principles of pattern differentiation and treatment in Traditional Chinese Medicine, CBD formulations applied in clinical practice demonstrate significant value in preventing, treating, and delaying the progression of kidney disease. Modern research confirms that CBD formulation and their active metabolites can modulate mitochondrial quality through specific pathways, thereby exerting therapeutic effects on CKD. While current evidence supports the potential of CBD in modulating MQC, it is important to note that many studies are preliminary and lack mechanistic depth. For instance, the specific bioactive metabolites responsible for the observed effects are often unidentified, and the interactions within multi-CBD formulations remain poorly understood. Moreover, the majority of studies are confined to *in vitro* or rodent models, raising questions about their relevance to human pathophysiology. This review aims to elucidate how CBD intervenes in and treats the progression of CKD by regulating the MQC system. The objective is to promote the clinical application of CBD and provide a theoretical basis for its use in CKD interventions.

A total of 264 articles were initially identified from PubMed, Web of Science, Embase, and Scopus using keywords such as “chronic kidney disease,” “mitochondria,” and “Chinese botanical drugs,” focusing primarily on studies published within the last 10 years. A limited number of older references were also included. The articles were then screened according to their relevance to the research topic and full-text availability. After applying these criteria, a final selection of 76 articles was made. For detailed information, refer to Supplementary Material 1.

## 2 Role of the MQC system in the pathogenesis of CKD

**FIGURE 1:**
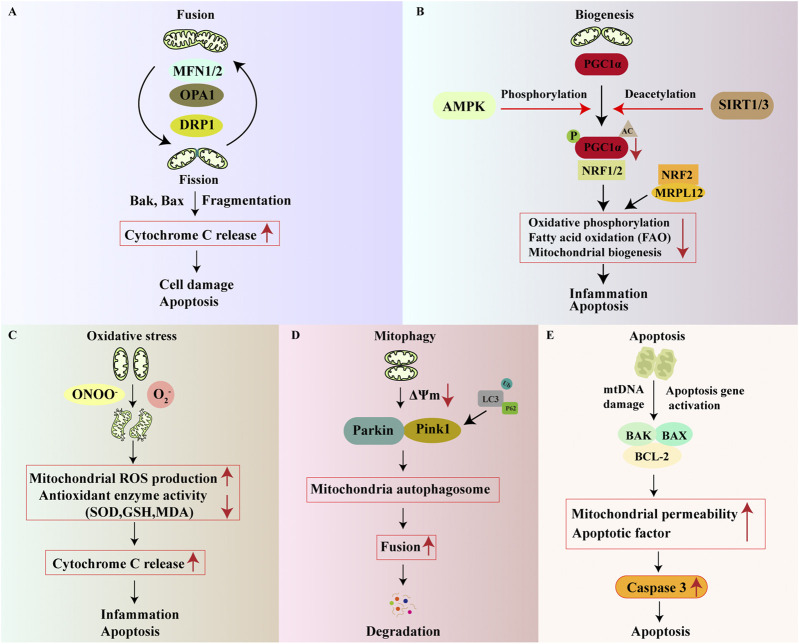
Molecular regulatory mechanisms of MQC. The main mechanisms of MQC include mitochondrial dynamics, mitochondrial biogenesis, mitochondrial oxidative stress, mitophagy, and mitochondrial apoptosis. **(A)** Mitochondrial fusion and fission jointly maintain mitochondrial dynamics stability. MFN1/2 and OPA1 are key regulators of mitochondrial fusion, while DRP1 is a key regulator of mitochondrial fission. When fusion and fission are imbalanced, cytochrome C (Cyt C) release increases, ultimately leading to mitochondrial apoptosis; **(B)** PGC-1α plays a central role in mitochondrial biogenesis. Several regulators, including SIRT and AMPK, are involved in the regulation of PGC-1α expression and activity. Reduced PGC-1α expression leads to weakened mitochondrial biogenesis, promoting inflammation and apoptosis; **(C)** When mitochondria undergo oxidative stress, the activity of their antioxidant enzymes and molecules (SOD, GSH) decreases, leading to increased Cyt C release and promoting inflammation and apoptosis; **(D)** Maintenance of MMP is a prerequisite for inhibiting abnormal mitophagy. A decrease in membrane potential stimulates the activation of the Parkin/PINK1 pathway, leading to the formation of autophagosomes, ultimately resulting in mitophagy and degradation; **(E)** mtDNA damage and/or activation of apoptotic genes leads to increased expression of pro-apoptotic/anti-apoptotic protein ratio (Bax/Bcl-2), which in turn activates downstream caspase-3 expression, resulting in mitochondrial apoptosis.

**FIGURE 2:**
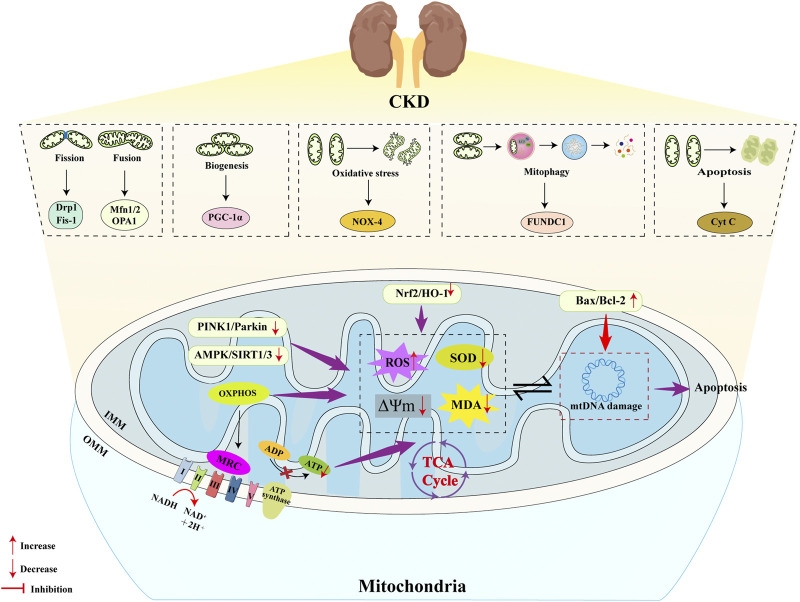
Molecular mechanisms of MQC in CKD. Multiple pathways within MQC have been identified as contributing to the onset and progression of CKD. In terms of mitochondrial dynamics, CKD patients manifest elevated mitochondrial fission and diminished fusion, a phenomenon that contributes to CKD progression via elevated DRP1 expression and reduced OPA1 levels. PGC-1α has been identified as a pivotal regulatory factor in the process of mitochondrial biogenesis. In CKD patients, PGC-1α expression is significantly suppressed, leading to reduced mitochondrial biogenesis through inhibition of the AMPK/SIRT1/3 signaling pathway. Mitochondria possess an antioxidant system to counteract ROS, but in CKD, the Nrf2/HO-1 pathway is inhibited, weakening the mitochondrial antioxidant system and increasing mitochondrial oxidative stress, thereby accelerating CKD progression. Mitophagy is a self-protective mechanism that maintains cellular homeostasis by clearing damaged mitochondria. However, in cases of CKD, the mitophagy pathway PINK1/Parkin is inhibited, thereby preventing damaged mitochondria in the kidneys from being cleared promptly. This, in turn, accelerates the progression of CKD over time. In CKD patients, there is an increase in mitochondrial apoptosis, which is driven by an imbalance in the pro-apoptotic/anti-apoptotic protein ratio (Bax/Bcl-2). These proteins cause mtDNA damage and increase mitochondrial oxidative stress, which in turn leads to further mitochondrial apoptosis.

Mitochondria serve as metabolic hubs and signaling platforms, mediating fundamental cellular processes including ATP production via oxidative phosphorylation (OXPHOS), cellular catabolism, nutrient signal regulation, and maintenance of protein homeostasis (Bennett et al., 2022). To sustain cellular stability, cells have evolved an intricate, nuclear-mitochondrial genome-coordinated MQC system (Choong et al., 2021). Reactive oxygen species (ROS) play an indispensable role in cells under physiological conditions. However, excessive amounts of ROS have the potential to induce damage to the inner mitochondrial membrane (IMM) and mitochondrial DNA (mtDNA). In response, cells initiate the activation of antioxidant defense systems, thereby ensuring the preservation of mitochondrial integrity. Furthermore, disruption of the balance between mitochondrial structure and function leads to the release of related apoptotic proteins, thereby driving the apoptosis process (Marchi et al., 2023). MQC, the core mechanism for maintaining mitochondrial quantity and functionality, comprises a dynamic system involving biogenesis, dynamics, mitophagy, oxidative stress response, and apoptosis. Its key is balancing impaired mitochondrial elimination and *de novo* generation of functional mitochondria (Ashrafi and Schwarz, 2013). In the sections that follow, we will explore several critical mechanisms involved in the MQC system (Figure 1) and discuss recent progress in understanding CKD (Figure 2).

### 2.1 Role of mitochondrial biogenesis in the pathogenesis of CKD

Mitochondrial biogenesis refers to the process of generating new mitochondria to enhance mitochondrial quantity and quality, thereby meeting cellular energy demands. It involves intricate steps, including the synthesis of inner/outer mitochondrial membranes (IMM/OMM), mitochondrial-encoded protein synthesis, nuclear-encoded mitochondrial protein import, and mtDNA replication, which requires coordinated regulation of nuclear and mitochondrial genomes (Fontecha-Barriuso et al., 2020; Jamwal et al., 2021; Popov, 2020). Research (Scarpulla, 2011; Scarpulla et al., 2012) indicates that mitochondrial biogenesis is modulated by transcriptional coactivators and co-repressors, among which the PGC-1 family (PGC-1α, PGC-1β, PRC) acts as a pivotal regulator of mitochondrial biogenesis and energy metabolism. Initially identified by Puigserver et al. (Puigserver et al., 1998) as a peroxisome proliferator-activated receptor-γ (PPARγ)-interacting protein, PGC-1α is highly expressed in high-energy-demand tissues (e.g., heart, kidney). It directly targets transcription factors to regulate nuclear genes: it upregulates nuclear respiratory factors 1/2 (Nrf1/2) and strengthens their binding to DNA. Activation of Nrf1/2 promotes mtDNA replication/transcription via mitochondrial transcription factor A (TFAM) and enhances transcription of nuclear-encoded mitochondrial electron transport chain subunits (Chambers and Wingert, 2020; König and McBride, 2024).

The PGC-1 family is abundantly expressed in the kidney, making it a promising therapeutic target for renal diseases (Svensson et al., 2016; Whitaker et al., 2016). A study (Rasbach and Schnellmann, 2007) demonstrated that PGC-1α critically regulates transcriptional programs of OXPHOS, tricarboxylic acid (TCA) cycle, and fatty acid metabolism in the kidney: KEGG-based transcriptome analysis of 4 mouse groups showed reduced expression of the OXPHOS, the TCA cycle, and glycolysis-related transcripts in PGC-1α knockout mice, indicating that renal PGC-1α inactivation impairs mitochondrial function, metabolic activity, and biogenesis. Consistently, low PGC-1α expression is observed in CKD, as validated in experimental CKD models and CKD patients’ kidneys (Platt and Coward, 2017). Conversely, PGC-1α expression alleviates oxidant-induced mitochondrial dysfunction, further confirming its role in maintaining mitochondrial homeostasis (Yuan et al., 2012).

Impaired mitochondrial biogenesis and reduced PGC-1α are common in CKD etiologies, particularly DKD, with DKD progression linked to the PGC-1α signaling pathway (Dugan et al., 2013; Hasegawa et al., 2013; Morigi et al., 2015; Tran et al. 2016) highlighted that mitochondrial dysfunction exacerbates ischemia-reperfusion injury (IRI)-induced renal damage via renal fatty acid accumulation, and replenishing NAD+ (a PGC-1α activation byproduct) improves mitochondrial health and mitigates renal damage progression. Kang et al. (Kang et al., 2015) showed that tubule-specific PGC-1α overexpression enhances renal tissue structure in CKD mice. PGC-1α′s role in mitochondrial biogenesis is regulated by post-translational modifications (acetylation, phosphorylation, methylation, ubiquitination) (Tang, 2016). For example, Sirtuin 1/3 (SIRT1/3) mediates PGC-1α deacetylation, and Yuan et al. (2012) demonstrated that SIRT1-dependent PGC-1α deacetylation alleviates aldosterone-induced podocyte injury, with the SIRT1 activator resveratrol protecting mitochondrial function. Notably, proximal tubule SIRT1 overexpression reduces diabetic glomerular pathology but paradoxically worsens glomerular injury in db/db mice. Despite PGC-1α′s implication in metabolic diseases (e.g., obesity, diabetes), its strong cell specificity poses challenges for therapeutic targeting, driving interest in developing novel biopharmaceuticals to improve mitochondrial function.

### 2.2 Role of mitochondrial dynamics in the pathogenesis of CKD

Mitochondria, being highly adaptable organelles, continuously modify their shape and size via fusion and fission, processes collectively known as mitochondrial dynamics. These changes are responses to metabolic and signaling cues in the cellular environment. Consequently, mitochondrial dynamics represent a pivotal process in the MQC system. Mitochondrial fission splits a single mitochondrion into two suborganelles, while fusion merges the OMM/IMM of two mitochondria to form a larger organelle. Excessive fission causes mitochondrial fragmentation, whereas enhanced fusion leads to hypertrophy (Bhatia et al., 2020). A precise balance between fission and fusion is critical for optimal mitochondrial function; disruptions in this balance trigger mitochondrial failure and cellular damage.

Dynamin-related protein 1 (Drp1), a large dynamic protein-related GTPase, has been shown to mediate mitochondrial fission (Jenner et al., 2022). During fission, Drp1 is recruited to the OMM; Bax/Bak-driven permeabilization signals mitochondrial fragmentation and apoptosis onset, accompanied by increased fission activity (while fusion is preserved) during cellular injury (Jenner et al., 2022; Zhan et al., 2013). Drp1 activity is regulated via post-translational modifications, including phosphorylation, ubiquitination, sumoylation, and S-nitrosylation (Galvan et al., 2017). Under stress, receptor proteins facilitate Drp1 translocation from the cytoplasm to mitochondria, where oligomerization drives mitochondrial constriction and cleavage. Elevated mitochondrial fission promotes CKD onset and progression: Drp1 deletion in renal tubular cells inhibits mitochondrial division and apoptosis (thus suppressing tubular mitosis in mice) (Srivastava et al., 2023), while podocyte-specific Drp1 knockout protects against DKD in mice—evidenced by reduced proteinuria, improved mesangial matrix expansion, and restored podocyte processes (Ayanga et al., 2016). In summary, inhibiting Drp1-mediated fission thus represents a potential strategy to slow CKD progression.

Mitochondrial fusion occurs in two stages: OMM fusion followed by IMM fusion, facilitated by OMM-localized mitofusins (MFN1/2) and IMM-localized optic atrophy 1 (OPA1) (Bhatia et al., 2020). OMM fusion depends on MFN1/2 dimerization and is responsive to stimuli like OS, while IMM fusion is regulated by metabolic changes in OPA1’s proteolytic cleavage sites (Song et al., 2007). In a physiological state, OPA1 exists primarily in a soluble, elongated form; however, ATP-dependent zinc metalloproteinase YME1L or metallopeptidase OMA1 (activated during mitochondrial membrane potential [MMP] loss) cleaves OPA1 into a shorter soluble isoform (Mishra et al., 2014). Both long and short OPA1 isoforms are essential for fusion, promoting minor structural changes under stable conditions. In CKD patients, downregulated MFN1/2 and OPA1 induce mitochondrial fragmentation, triggering apoptosis/necrosis and accelerating tubular atrophy/interstitial fibrosis. Liu et al. (2020b) showed that AMPK signaling and PGAM5 (a mitochondrial fission regulator) contribute to mitochondrial fission during diabetic tubular injury. In diabetic tubular lesions (HK-2 cells and mice), stromal cell-derived factor-1α (SDF-1α), which is a substrate of dipeptidyl peptidase 4 (DPP4), prevents downstream STAT3 (Ser727) phosphorylation and STAT3 mitochondrial translocation by blocking SDF-1α/CXCR4 signaling, resulting in increased mitochondrial fragmentation and disruption of OPA1 function (Zhang Q. et al., 2020). Another study (Liu et al., 2022) found that highly fragmented and dispersed mitochondria in human podocytes were induced by high glucose (HG). Reduced expression of OPA1 and MFN1/2, as well as increased expression of Drp1, in podocytes cultured under HG conditions, is reversed to alleviate excessive mitochondrial fragmentation and cell damage in podocytes. Notably, proximal tubule-specific MFN2 deletion accelerates recovery and improves survival post-renal IRI in animals (Gall et al., 2015). In CKD patients, reduced MFN1/2 impairs fusion, decreasing renal ATP synthesis and triggering cell damage and apoptosis.

### 2.3 Role of mitophagy in the pathogenesis of CKD

Mitophagy is a complex, multifactorial cellular response that depends on energy, stress, and signaling environments. It selectively removes excess or damaged mitochondria and plays a vital role in regulating the number of mitochondria within cells and maintaining normal mitochondrial function (Onishi et al., 2021). Autophagy, in general, is orchestrated by autophagy-related proteins (ATGs) and proceeds through five stages: induction, phagophore nucleation, elongation, autophagosome maturation, and lysosomal fusion (Yao et al., 2021). Classified by trigger conditions and receptor dependence, autophagy includes selective and non-selective subtypes; mitophagy, a key selective autophagy process, is governed by multiple signaling pathways (Gatica et al., 2018). Two classical pathways mediate mitophagy: ubiquitin-dependent and ubiquitin-independent mechanisms, as outlined below.

The ubiquitin-dependent pathway relies on ubiquitination of mitochondrial surface proteins to drive mitophagy, with the PINK1/Parkin axis being the most well-characterized in mammals (Imberechts et al., 2022; Li J. et al., 2023). PINK1, an evolutionarily conserved mitochondrial protein with a kinase domain and mitochondrial targeting sequence, is constitutively imported into the IMM and degraded by proteases under normal conditions, maintaining low expression levels. Parkin, a cytoplasmic E3 ubiquitin ligase, mediates targeted protein ubiquitination (Han H. et al., 2023). Upon mitochondrial depolarization (a hallmark of damage) (Han R. et al., 2023), PINK1 degradation is inhibited, leading to its accumulation on the OMM, where it ubiquitinates OMM proteins and is activated via phosphorylation (Fiesel et al., 2023). Phosphorylated PINK1 further phosphorylates ubiquitin at Ser65, which recruits and activates Parkin; activated Parkin then polyubiquitinates multiple mitochondrial substrates (Dunkerley et al., 2022). Ultimately, under the action of the autophagy-related protein light chain 3 (LC3) adapter protein, autophagosomes are targeted to mitochondria, inducing mitophagy.

Ubiquitin-independent mitophagy involves OMM-localized receptors—including NIX (BNIP3L), BNIP3, and FUNDC1—that directly bind LC3 without ubiquitination. BNIP3, a BH3-only Bcl-2 family protein, interacts with LC3/GABARAP independently of adaptors or ubiquitination (Hanna et al., 2012). As demonstrated in a previous study (Hendgen-Cotta et al., 2017), BNIP3 and NIX form homo/heterodimers to maintain mitochondrial homeostasis and interact with Mieap (mitochondrial engulfment protein) and CDH6 (cadherin 6) to regulate ROS clearance and Drp1-mediated fission (Nakamura et al., 2012). Additionally, a recent study (Dong et al., 2022) indicates that FUNDC1 directly binds to LC3 on the OMM, thereby functioning as an autophagy receptor during mitosis under hypoxic conditions. Its activity is controlled by phosphorylation/dephosphorylation, with UNC-51-like kinase 1 (ULK1)-mediated phosphorylation critical for recruiting damaged mitochondria and initiating mitophagy (Zhu et al., 2022). Therefore, impaired mitophagy leads to the accumulation of dysfunctional mitochondria, accelerating CKD progression.

Recent studies (Aggarwal et al., 2016; Liang and Kobayashi, 2016; Zhu et al., 2013) have emphasized that the kidney exhibits higher basal mitophagy activity than other organs, a key factor in mitochondrial homeostasis. In the early stages of CKD, the body employs autophagy to clear damaged mitochondria and maintain normal cell function. However, with the disease’s advance, progressive mitochondrial damage overwhelms this system in late-stage CKD, triggering apoptosis. DKD, a major cause of end-stage renal failure, is linked to mitophagy dysfunction: DKD models (human and animal) show accumulated mitochondrial fragments, swollen mitochondria, and mitophagosome aggregation (Chen et al., 2018; Higgins and Coughlan, 2014; Xiao et al., 2017). Inhibition of mitophagy reverses PGRN-associated mitochondrial preservation via the PGRN/SIRT1/PGC-1 pathway, which regulates forkhead box protein O1 (FoxO1). In diabetic mice and HG-treated renal tubules, the two key regulatory factors of mitochondrial phagocytosis, PINK1 and Parkin, were sharply reduced, impairing mitochondrial renewal. MitoQ, a mitochondrial-targeted antioxidant, has been shown to impede DKD progression by attenuating activation of the mitochondrial ROS-TXNIP/NLRP3/IL-1 axis. In experimental DKD models, MitoQ has been observed to prevent tubular damage through mitochondrial phagocytosis, a process that is facilitated by nuclear factor Nrf2 and PINK1. CoQ10, a distinct mitochondrial-targeted antioxidant, has been shown to exert positive effects on *in vivo* and *in vitro* DKD models by promoting Nrf2 signaling (Sun et al., 2019). FoxO1 activation prevents HG-induced damage by preventing mitochondrial dysfunction in the rat renal cortex (Li et al., 2016). Specifically, suppressing PTEN activates the PINK1/Parkin pathway, while FoxO1 upregulation restores damaged podocytes in DN mice (Li W. et al., 2017). Regardless of the type of damaged cells, impaired mitochondria release pro-apoptotic factors and increase ROS production to perpetuate the vicious cycle, thereby propagating cellular damage. In summary, these studies reveal a close association between mitophagy and CKD, suggesting that mitigating mitochondrial oxidative damage can prevent tubular injury and offering novel therapeutic strategies for CKD.

### 2.4 Role of mitochondrial oxidative stress in the pathogenesis of CKD

Oxidative stress (OS) is a physiological disorder resulting from an imbalance between excessive ROS production and the antioxidant defenses of the organism (Balaban et al., 2005). ROS are byproducts of cellular aerobic metabolism and play an important role in cellular signaling. More specifically, ROS are a catch-all term for a diverse array of metabolites and free radicals that spring from oxygen molecules, including the superoxide anion (O2-), hydrogen peroxide (H2O2), the hydroxyl radical (OH-), and oxygen (O2) (Sies, 2015). Mitochondrial reactive oxygen species (mtROS) are a result of the respiratory chain, especially from NADH dehydrogenase (complex I) and ubiquinone cytochrome oxidoreductase (complex III) (Brand, 2010). The mitochondrial respiratory chain, situated in the IMM, is primarily made up of complex I, succinate-fumarate dehydrogenase, also known as complex II, complex III, and the enzyme Cyt C oxidase, otherwise termed complex IV. Moreover, ATP synthase, which is Complex V, plays a key role in ATP synthesis during OXPHOS in the mitochondria. This process also involves two electron shuttles, ubiquinone (CoQ) and Cyt C (Vercellino and Sazanov, 2022). The generation of mitochondrial reactive oxygen species (mtROS) is primarily driven by oxidative phosphorylation (OXPHOS) efficiency, the oxidation of NADPH/NADH, and the synthesis of heme and iron-sulfur clusters (Read et al., 2021; Su et al., 2023). When OXPHOS is active, electrons escape from complexes I and III in the mitochondria, reacting with oxygen to form superoxide anions, the most harmful type of mtROS. Research indicates that mitochondrial complex I plays a key role in shuttling electrons from TCA cycle-derived NADPH/NADH to oxygen, thereby promoting NADPH/NADH oxidation and superoxide radical formation. These radicals, in turn, trigger a substantial surge in mtROS production (Albracht et al., 2011). Excessive production of mtROS can induce OS in lipids and proteins, and DNA damage. To prevent further cellular damage, the organelles mitigate the trouble by neutralizing superoxide through the concerted actions of Mn-SOD, catalase (CAT), and glutathione peroxidase (GPx), thereby preserving cellular homeostasis (Ismail et al., 2019). A mounting body of evidence (Ning et al., 2021) indicates that the MQC system exerts a pivotal function in mtROS-mediated redox imbalance. Mitochondria have been shown to limit excessive mtROS production and maintain redox balance by phagocytosing aged and damaged mitochondria. For instance, research indicates that long-term exposure to PM2.5 can drive excessive mitochondrial ROS and undermine the mitochondria’s ability to engage in mitophagy, leading to a redox imbalance. On the other side, adding antioxidants that target mitochondria, like coenzyme Q, can enhance mitochondrial mitophagy activity and curb oxidative harm by limiting ROS buildup. Additionally, agents that inhibit pathways such as AMPK, MAPK, and Nrf2 have been shown to promote ROS clearance by boosting how actively mitochondria participate in phagocytosis. In turn, this supports the upkeep of mitochondrial redox equilibrium (Chen D. et al., 2024; Esteras and Abramov, 2022; Franci et al., 2022; Lu X. et al., 2021). Furthermore, under conditions of hypoxia, mitochondria have been observed to promote widespread ubiquitination through the UPS, thereby activating receptor-dependent mitosis, such as BNIP3/Nix, to alleviate mtROS accumulation and OS levels. A recent study (Ashraf and Kumar, 2022) has reported that increased MFN2 expression promotes mitochondrial fusion and autophagy, reduces reactive oxygen species, and thereby maintains redox balance. *In vitro* experiments confirmed that increasing the GSSG/glutathione ratio led to cis-oligomerization of MFN disulfide bonds and promoted mitochondrial fusion. A study (Lloberas et al., 2020) has confirmed that the C684 residue is essential for Mfn2 disulfide bonds and fusion activity. When C684 is absent, Mfn2 is more susceptible to redox changes, affecting mitochondrial energy output. Moreover, research has substantiated that MFN2 is a crucial player in the induction of mitochondrial respiratory stress and the production of reactive oxygen species. Disrupting MFN2 in macrophages results in subpar ROS synthesis and compromised immune function. Furthermore, as oxidative stress intensifies, the activation of the Nrf2 pathway does not just bolster antioxidant defenses and reinstate the redox equilibrium; it also facilitates the degradation of the mitochondrial fission protein DRP1, which in turn lessens mitochondrial division. To sum it up, when dealing with OS, cells can safeguard against ROS build-up and restore redox balance by fine-tuning the MQC system.

The kidneys are a reservoir of mitochondria and require a large amount of energy, but this enormous energy consumption can sometimes lead to increased OS. CKD, including DKD, glomerulosclerosis, glomerulonephritis, tubulointerstitial fibrosis, and chronic renal transplant dysfunction chronic allograft nephropathy (CAN), is predominantly influenced by OS (Daenen et al., 2019). Mitochondrial dysfunction is a key driver in the onset and advancement of DKD, primarily by interfering with cellular energy balance and exacerbating OS. Research (Sharma, 2015) indicates a significant drop in MMP, which disrupts respiratory control and ultimately diminishes ATP synthesis while increasing ROS generation. Hyperglycemia and ROS production disrupt cellular metabolism, leading to increased production of electron donors (such as NADH and flavin adenine dinucleotide) through the tricarboxylic acid cycle, which may overwhelm the mitochondrial electron transport chain and result in excessive ROS production (Brownlee, 2005). Both lab and clinical studies demonstrate that glucose-triggered ROS plays a direct role in podocyte death and loss, accelerating kidney damage. Mn-SOD, an antioxidant enzyme, plays a regulatory role in the management of ROS within the mitochondria. Research has found that in STZ-induced rat models, Mn-SOD activity is suppressed both in the early stages and throughout the course of DM (Coughlan et al., 2016). These studies show for the first time that inducing high blood sugar levels in mitochondria to produce superoxide triggers specific mitochondrial damage (i.e., complex III) through a mechanism independent of Mn-SOD inactivation. Moreover, there’s a marked increase in the activity of the NAD+ breakdown enzyme, CD38. However, SIRT3 knockout exacerbated the aforementioned pathological phenomena (Ogura et al., 2018). In-depth studies on CD38 and SIRT3-related oxidative stress have revealed that in a rat model of type 2 diabetes mellitus (T2DM), mitochondrial oxidative stress is accompanied by an increase in the levels of the NAD+ -degrading enzyme CD38. Additionally, the intracellular NAD+/NADH ratio and SIRT3 activity are reduced, while the expression of the NAD+ degrading enzyme CD38 is increased in the kidneys (Howard et al., 1993). Moreover, it was found that augmented expression plays a crucial role in the formation of DKD stemming from mitochondrial oxidative stress. The root of this process is tied to a reduction in the NAD+/NADH balance and the stimulation of SIRT3 (Ogura et al., 2020). In summary, the excess of CD38, the lessened function of SIRT3, and the lowered NAD+/NADH ratio in high glucose situations contribute to diabetic tubular damage by boosting mitochondrial oxidative stress.

### 2.5 Role of mitochondrial apoptosis in the pathogenesis of CKD

Mitochondrial apoptosis, the primary intrinsic pathway of programmed cell death, is activated by stimuli including mtDNA damage, endoplasmic reticulum (ER) stress, and cellular OS, and is primarily regulated by Bcl-2 family proteins (Elmore, 2007; Zhang Z. et al., 2024). The anti-apoptotic protein Bcl-2 is typically located on the OMM and primarily functions to inhibit the release of Cyt C into the cytoplasm. Conversely, the pro-apoptotic protein Bax resides in the cytoplasm and acts in direct contrast to Bcl-2. Notably, the Bax/Bcl-2 ratio serves as a key regulatory factor in mitochondrial apoptotic signaling (Kaplan et al., 2020). The initial hallmark of mitochondrial impairment is a decrease in MMP, which precedes the onset of nuclear condensation and DNA fragmentation. Once the mitochondrial apoptosis pathway is activated, it primarily modifies the expression of pro-apoptotic proteins such as Bax, Bak, Bim, Puma, and Noxa, as well as anti-apoptotic proteins like Bcl-2, Bcl-xl, Bcl-w, Mcl-1, and Bcl-G, decreasing MMP, opening the mitochondrial permeability transition pore (MPTP), leading to the release of Cyt C into the cytoplasm, where it binds with apoptosis-inducing factor (AIF), Apaf-1, Smac to form more apoptotic bodies, promoting the activation of caspase-3/7/8/9, activating the caspase cascade reaction, and ultimately inducing the mitochondrial apoptosis pathway in cells (Gizem Özkan et al., 2022; Zhou et al., 2023).

In damaged kidneys, renal tubular epithelial cells and podocytes are the primary cell types undergoing apoptosis (Shankland, 2006). Mitochondrial dysfunction contributes significantly to tubular epithelial damage, exacerbated by conditions such as IRI, rhabdomyolysis, and hyperglycemia. Zhan et al. (2015) demonstrated that high glucose induced mitochondrial fragmentation *in vitro* in human proximal tubular epithelial cells (HK-2) and porcine proximal tubular cells (LLC-PK1), accompanied by increased Drp1/Fis1 expression, decreased MFN2 expression, and unchanged OPA1 levels. In a study by Brooks et al. (2009), it was observed that renal tubular epithelial cells in rats subjected to an acute kidney injury model of IRI exhibited augmented mitochondrial fragmentation and Cyt C release. The inhibition of Drp1 expression and function led to a reduction in mitochondrial fragmentation, the maintenance of mitochondrial morphology, and an improvement in renal lesions. Xiao et al. (2014) found that mitochondrial metalloproteinase OMA1, which can hydrolyze OPA1, inhibits the role of OPA1 in promoting inner mitochondrial membrane fusion, leading to mitochondrial fragmentation. OMA1 deficiency has been demonstrated to exert a protective effect on IRI-induced renal tubular epithelial cell apoptosis. Furthermore, Tang et al. (2013) found that when rhabdomyolysis causes damage to renal tubular epithelial cells, there is an increase in the translocation of Drp1 to mitochondria, an increase in mitochondrial fragmentation, a decrease in ATP production, and an increase in ROS production, ultimately leading to Cyt C release and renal tubular epithelial cell apoptosis.

## 3 Gut microbiota dysbiosis and mitochondrial dysfunction in CKD

**FIGURE 3:**
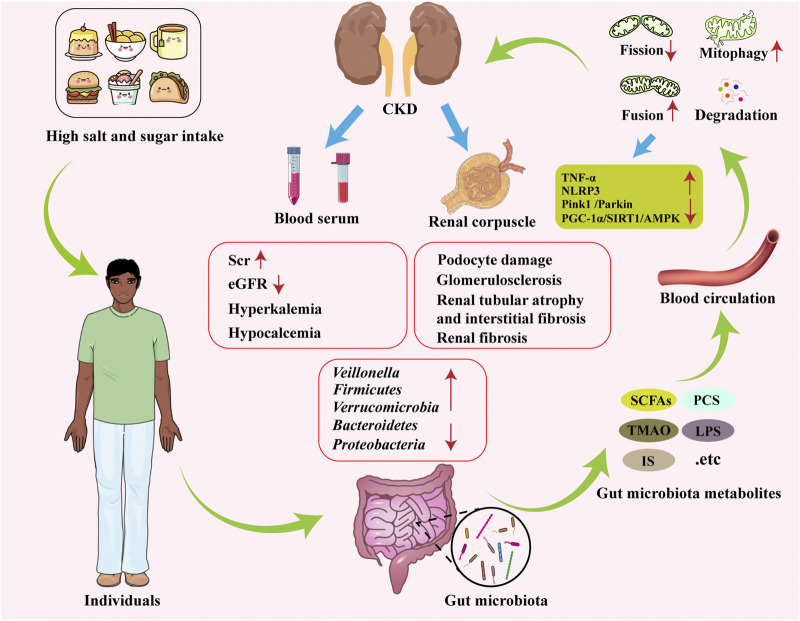
Role of the gut microbiota-microbial metabolites-mitochondrial axis in the pathogenesis of CKD. With increased intake of high-salt and high-sugar foods, the diversity and abundance of the gut microbiota undergo changes, characterized by decreased Proteobacteria and Bacteroidetes, and increased Firmicutes. Various gut microbial metabolites, such as SCFAs, TMAO, and PS, are released into the bloodstream. These metabolites may lead to alterations in mitochondrial function, thereby influencing the progression of CKD. SCFAs: short-chain fatty acids, TMAO: trimethylamine N-oxide, IS: indoxyl sulfate, PS: phenyl sulfate, Scr: serum creatinine, eGFR: estimated glomerular filtration rate.

Recent studies (Lei et al., 2025; Ma et al., 2023) on the interplay between gut microbiota alterations, microbial metabolites, and mitochondrial dysfunction have provided new perspectives for CKD research. Specifically, the common mitochondrial dysfunction and gut dysbiosis observed in CKD patients may jointly promote uremic toxin accumulation and vascular injury through mechanisms such as OS and inflammation. Moreover, existing research increasingly highlights the role of the gut microbiota-microbial metabolites-mitochondria axis in the pathophysiology of CKD progression, which can influence disease progression by regulating the MQC system (Figure 3). The following section briefly elaborates on this crosstalk.

### 3.1 Gut microbiota and their metabolites in CKD

The gut microbiota refers to the normal microorganisms residing in the human gastrointestinal tract, such as *Bifidobacteria* and *Lactobacillus*. These bacteria synthesize various vitamins essential for human growth and development. Based on bacterial abundance, the gut microbiota can be categorized into dominant and subdominant microbial communities. Dominant microbiota include the *Firmicutes, Bacteroidetes, Ruminococcus*, and *Bifidobacterium*, which typically belong to core flora and determine the host’s physiological and pathophysiological status (Sabatino et al., 2015). Numerous studies (Holmes et al., 2020) have demonstrated that gut microbiota dysbiosis is closely associated with the development and progression of CKD. In CKD rat models, bacterial abundance in the family *Veillonella* significantly increases. Patients with CKD exhibit increased proportions of pathogenic bacteria and reduced levels of probiotics (e.g., *Bifidobacteria*), forming abnormal microbial communities dominated by strict anaerobes (increased *Firmicutes* and *Veillonella*; decreased *Proteobacteria* and *Bacteroidetes*).

Numerous studies currently indicate that common gut microbiota metabolites such as short-chain fatty acids (SCFAs), trimethylamine N-oxide (TMAO), p-cresol sulfate (PCS), indole-3-carboxylic acid sulfate (IS), and lipopolysaccharides (LPS) are involved in the pathological progression of CKD. For instance, dysbiosis and dietary imbalances lead to abnormal SCFA production, reducing GPCR41 and GPCR43 activation. This disrupts the release of peptide YY (PYY) and glucagon-like peptide-1 (GLP-1), thereby contributing to CKD pathogenesis (Lafferty et al., 2018; Lu et al., 2016). Plasma IS and PCS levels are significantly elevated in CKD patients, highly correlated with disease progression and proteinuria levels. It is noteworthy that serum IS levels begin to rise in the early stages of CKD compared to healthy individuals (Sabatino et al., 2017). TMAO, a product of gut microbiota metabolism of dietary choline metabolites, has been clinically demonstrated (Zhuang et al., 2019) to promote NF-κB pathway activation in CKD patients, further exacerbating systemic microinflammation and contributing to CKD progression (Al-Obaide et al., 2017). Recent findings (Kikuchi et al., 2019) indicate that elevated plasma phenyl sulfate (PS) levels in rats cause more severe glomerular damage, and plasma PS levels significantly correlate with proteinuria/creatinine ratio and estimated glomerular filtration rate in CKD patients. In summary, CKD-induced gut microbiota dysbiosis accelerates disease progression by promoting the synthesis of toxic metabolites.

### 3.2 The gut microbiota-microbial metabolite-mitochondria axis in CKD

The endosymbiotic theory suggests that mitochondria originated from bacteria and share commonalities with gut microbiota in physiological characteristics, structure, and metabolism (Ghosh et al., 2022; Tomtheelnganbee et al., 2022). First, the endosymbiotic theory posits that mitochondria evolved from bacterial ancestors (Andersson et al., 2003), indicating an intrinsic connection in their origin and differentiation. Moreover, host mitochondria can influence gut microbiota diversity by releasing ROS, suggesting that gut microbiota and mitochondria indeed engage in biological “conversations” affecting health and disease (Yardeni et al., 2019). Second, gut microbiota metabolites may serve as mediators of this interaction. Thiele et al. (2013) demonstrated through metabolomics that mitochondrial and gut microbiota metabolites exhibit high overlap, suggesting these metabolites may be key mediators in their mutual influence.

Existing research confirms that gut microbiota metabolites regulate mitochondrial function and contribute to CKD pathogenesis. For instance, SCFAs (acetic acid, butyric acid, and propionic acid) are primary fermentation products of colonic bacteria, exerting multiple beneficial effects on regulating intestinal barrier integrity, inflammation, and immune responses, as well as glucose and lipid metabolism. During exercise, gut microbiota modulate mitochondrial biogenesis via PGC-1α/SIRT1/AMPK pathways, and their metabolites (e.g., SCFAs, secondary bile acids) mitigate TNF-α-mediated immunity and reduce NLRP3 inflammasome activation, thereby regulating mitochondrial energy production and ROS generation to alleviate intestinal inflammation (Clark and Mach, 2017). Indeed, butyrate supplementation increases mitochondrial respiration and PGC-1α expression in mice (Gao et al., 2009). Under disease conditions, certain pathogenic bacteria degrade sulfur-containing amino acids to produce excessive hydrogen sulfide (H_2_S), which impairs mitochondrial respiratory chains by inhibiting cytochrome oxidase activity. In newborns with Crohn’s disease, increased H_2_S-producing microbiota and downregulation of mitochondrial H_2_S detoxification proteins jointly cause mitochondrial dysfunction, while bismuth-mediated H_2_S clearance alleviates *Clostridium* difficile-induced colitis (Mottawea et al., 2016). Conversely, some studies (Hine et al., 2015; Meng et al., 2018; Pietri et al., 2011) indicate protective effects of H_2_S, such as reducing ROS formation regulated by Nrf2, increasing antioxidant gene expression, and mitigating inflammation, challenging its pro-inflammatory role and necessitating further investigation. The gut microbiota metabolizes amino acids such as tryptophan, tyrosine, and phenylalanine, producing uremic toxins like IS, PCS, and IAA, which promote OS through free radical formation (Borges et al., 2016; Stockler-Pinto et al., 2016). Consequently, IS pretreatment of T3-L1 adipocytes increases ROS production, activates NADPH oxidase, and elevates TNF-α/IL-6 secretion (Stockler-Pinto et al., 2016). In CKD patients, uremic toxins are also associated with elevated IL-6 and monocyte chemotactic protein-1 (Borges et al., 2016). Furthermore, Sato et al. (2016) observed IS accumulation in CKD mouse muscle, inducing mitochondrial dysfunction via Nrf2-mediated oxidative stress, altered metabolic flux, and reduced ATP availability. Collectively, gut dysbiosis leads to abnormal distribution of metabolites, promoting CKD progression by disrupting mitochondrial function, increasing ROS production, and enhancing inflammatory responses. New insights into the gut microbiota-microbial metabolites-mitochondria axis may be crucial for identifying novel therapeutic options for CKD.

## 4 CBD for regulating the MQC system in CKD

In recent years, CBD has been widely applied in clinical settings for various kidney diseases due to its multi-target and multi-pathway characteristics, achieving satisfactory therapeutic outcomes and being regarded as a promising alternative treatment method. CBD employs a nuanced approach to healing, harnessing the intricate pharmacological properties of natural flora. Through meticulous concoction and processing, these plants are transformed into formulation or their active metabolites are extracted and isolated. In the following chapters, we explore how natural chemical metabolites and CBD formulations are utilized to regulate the MQC system in the treatment of CKD.

### 4.1 Natural chemical metabolites for CKD treatment by regulating MQC

#### 4.1.1 Natural chemical metabolites improve mitochondrial biogenesis to alleviate CKD

##### 4.1.1.1 Polyphenols

Research on quercetin, a flavonoid abundant in many plants, including the botanical drug *Cuscuta epithymum (L.) L*., has demonstrated its potential to induce mitochondrial biogenesis. Existing research (Hennino et al., 2016) indicates that quercetin treatment mitigates TGF-β1-induced fibrosis in renal tubular epithelial cells (RTECs) by regulating microRNA-21 (miRNA-21) activity. Liu T. et al. (2020) reported that quercetin attenuated AngII-induced RTECs senescence *in vitro* and unilateral ureteral obstruction (UUO) *in vivo*. They found that AngII-treated RTECs exhibited elevated levels of mtROS, reduced membrane potential, and fragmentation, accompanied by increased mitochondrial mass. The Administration of quercetin alleviated these effects, thereby significantly delaying the aging process of rat RTECs and renal interstitial fibrosis.

Curcumin, a polyphenol derived from turmeric with a favorable safety profile and broad pharmacological activity, shows therapeutic potential in CKD (Ma et al., 2024). Studies across multiple experimental models highlight its role in mitigating mitochondrial dysfunction—a key feature of CKD pathology. Kuo et al. (2012) demonstrated that curcumin treatment in 5/6 nephrectomy (5/6 Nx) rats restored mitochondrial membrane potential, improved renal β-oxidation, and modulated lipid metabolism. Furthermore, curcumin has been shown to reduce hepatic lipogenesis and increase mitochondrial biogenesis markers, such as Nrf1 and TFAM. In a recent study, Wang D. et al. (2020) revealed that curcumin alleviates mitochondrial dysfunction in 5/6Nx-induced wild-type and muscle-specific GSK-3β gene knockout (KO) CKD model mice by increasing mitochondrial biogenesis, improving ATP levels, mitochondrial electron transport chain complex activity, and basal mitochondrial respiration, as well as inhibiting MMP. Additionally, the study also discovered that curcumin’s protective benefits are realized through the inhibition of GSK-3β activity in both *in vitro* and *in vivo* settings. GSK-3β KO helps improve mitochondrial function, reduce mitochondrial oxidative damage, and enhance mitochondrial biogenesis in the muscles of patients with CKD. Furthermore, in a gentamicin (GM)-induced nephrotoxicity model, curcumin activated the Nrf2/PGC-1α signaling pathway, preserving mitochondrial integrity and energy metabolism (Negrette-Guzmán et al., 2015). Collectively, these findings underscore curcumin’s potential to ameliorate CKD-related mitochondrial impairment through multiple mechanisms.

##### 4.1.1.2 Glycosides

Astragaloside IV (AS-IV), a major bioactive compound and quality marker of *Astragalus L.*, exhibits multiple pharmacological properties such as anti-inflammatory, anti-fibrotic, and antioxidant activities, with demonstrated renoprotective potential in CKD (Shen Q. et al., 2023; Zhang Q. et al., 2021). Research (Liu et al., 2024) has found that after stimulating NRK-52E cells with TGF-β1 for 48 h, the expression of mitochondrial biogenesis-related proteins PGC-1α, Nrf1, and TFAM was downregulated. The intervention with AS-IV resulted in a significant enhancement in the expression of mitochondrial biogenesis proteins and a simultaneous reduction in renal fibrosis. These results indicate that AS-IV may counteract renal fibrosis by enhancing mitochondrial biogenesis. As is well established, SIRT1, PGC-1α, Nrf1, and TFAM are critical factors in mitochondrial biosynthesis. Li L. et al. (2024) found that AS-IV could partially reverse the reduction in mitochondrial biogenesis and function-related indicators (SIRT1, PGC-1α, Nrf1, and TFAM) caused by PS *in vitro* and improve mitochondrial health. However, some studies report that AS-IV did not affect mitochondrial fusion proteins (MFN1/2, OPA1) or consistently alter PGC-1α and Nrf1 levels in diabetic models, suggesting context-dependent mechanisms (Liu X et al., 2017). Beyond mitochondrial biogenesis, AS-IV participates in other aspects of MQC. Research has demonstrated (Shen Q. et al., 2023) that AS-IV can enhance the expression of PINK1 and LC3II/I protein levels in the kidneys of DKD rats, while downregulating the expression of Drp1 protein, and effectively lowering blood glucose and proteinuria, thereby protecting renal function. AS-IV can also mitigate the decline of mitochondrial-specific electron transport chain complexes, ATP, and mtDNA in DKD kidney tissue, diminish ROS generation, and lessen kidney damage and podocyte apoptosis. Collectively, AS-IV appears to mitigate CKD progression through multi-faceted regulation of mitochondrial homeostasis.

Salidroside, a phenethyl alcohol glycoside derived from *Rhodiola rosea L.*, is known for its favorable safety profile and diverse pharmacological effects, including anti-hypoxic and anti-inflammatory properties (Zhang X. et al., 2021). Research on the beneficial effects of salidroside on the kidneys is ongoing. In a STZ-induced DKD mouse model, salidroside was shown to restore mitochondrial function by increasing mtDNA copy number and electron transport chain protein expression. It also reversed the downregulation of SIRT1 and PGC-1α, suggesting its action may involve SIRT1/PGC-1α-mediated mitochondrial biogenesis (Xue et al., 2019). Similarly, in glomerular endothelial cells under HG conditions (Wu et al., 2016), salidroside reduced albumin transport by modulating the AMPK/Src/Caveolin-1 signaling pathway and attenuated mitochondrial OS while moderately decreasing mitochondrial membrane potential. These findings indicate that salidroside alleviates DKD through multiple mechanisms, including the enhancement of mitochondrial biogenesis and the reduction of proteinuria via Caveolin-1 phosphorylation inhibition.

##### 4.1.1.3 Other categories

Berberine, an isoquinoline alkaloid of natural origin, has been identified in *Coptis chinensis Franch* (Zhang et al., 2016). As reported by Qin et al. (2020), PGC-1α was significantly downregulated in db/db mice and palmitic acid-induced apoptotic podocytes. Notably, berberine enhanced PGC-1α expression and improved mitochondrial function in both *in vitro* and *in vivo* studies. Leonurine, a natural alkaloid derived from *Leonurus cardiaca L.*, has been identified as a potential therapeutic agent. Leonurine has been demonstrated to enhance the expression of PGC-1α in podocytes treated with doxorubicin, thereby mitigating podocyte damage (Liu et al., 2018). Grape seed proanthocyanidin extracts (GSPE) are water-soluble flavonoid pigments widely found in fruits and vegetables, exhibiting various bioactivities such as anti-inflammatory and antioxidant properties (He et al., 2022). *In vitro* studies have shown (Bao et al., 2014) that GSPE increases the expression of mitochondrial TFAM, PGC-1α, and NRF-2. These elevated levels may influence AMPK phosphorylation and mitochondrial biogenesis. Sorbic acid (SA) is a natural phenolic metabolite with antioxidant, anti-inflammatory, and antibacterial properties, and is widely used in the prevention and treatment of diabetes, cardiovascular diseases, and tumors (Srinivasulu et al., 2018). In the context of DM rats, SA has been demonstrated to reverse the low mRNA expression of PGC-1α and NRF-1, augment the mtDNA/nDNA ratio, and diminish antioxidant enzyme activity, including CAT. These effects are concomitant with the regulation of mitochondrial biogenesis and OS, thereby mitigating mitochondrial damage (Rashedinia et al., 2020). Glycyrrhetic acid (GA) is the main metabolite of *Glycyrrhiza glabra L.* and belongs to the flavonoid metabolite family (Ban et al., 2020). In the aftermath of aluminum poisoning, the expression rate and quality of mitochondrial genes underwent a substantial augmentation in GA. The protective effect of GA against aluminum-induced toxicity has been demonstrated to be closely related to improvements in mitochondrial function and biogenesis (Rashedinia et al., 2019). Furthermore, GA has been shown to enhance the expression of AMPK, SIRT1, and Mn-SOD in renal tubular epithelial cells exposed to high glucose environment, thereby providing a protective effect on these cells (Hou et al., 2014).

The above studies in CKD models have provided compelling preliminary evidence of an association between natural chemical metabolites and enhanced mitochondrial biogenesis. However, several limitations must be acknowledged. Firstly, the concentrations of metabolites like quercetin and curcumin used in cell cultures (often in the µM range) may not be physiologically achievable in human kidneys following oral administration, raising questions about clinical relevance. Secondly, while upregulation of PGC-1α is a common finding, the upstream signaling events triggering this response are often not thoroughly investigated. The reliance on chemical inducers (e.g., TGF-β1) *in vitro* may not fully recapitulate the complex pathophysiology of human CKD. Furthermore, many studies report improved “mitochondrial function” based on a limited set of parameters (e.g., ATP, MMP); a more comprehensive assessment of respiratory chain complex activities, OXPHOS efficiency, and *in vivo* metabolic imaging would strengthen these conclusions. The promising effects of AS-IV and salidroside are notable, but their direct molecular targets within the biogenesis pathway remain largely unidentified.

#### 4.1.2 Natural chemical metabolites improve mitochondrial oxidative stress to alleviate CKD

##### 4.1.2.1 Polyphenols

Resveratrol (RSV) is a natural polyphenolic molecule extracted from *Polygoni Cuspidati Rhizoma et Radix*, and it serves as an excellent scavenger of ROS. In CKD patients, numerous signaling pathways are disrupted or even inactivated, leading to pathological changes, including renal tubular cell apoptosis and disruption of the intracellular environment. RSV has been shown to enhance the antioxidant capacity and self-repair ability of renal tubules by regulating multiple signaling pathways, including Nrf2/Keap1, SIRT1/3, and PGC-1α (Palsamy and Subramanian, 2011). As an activator of Nrf2, RSV facilitates the dissociation of the Keap1/Nrf2 complex, allowing Nrf2 to enter the cell nucleus and enhance the expression of antioxidant genes such as HO-1 and nicotinamide reductase (NQO1) within the cell, thereby maintaining cellular homeostasis and reducing oxidative stress response (Lee and Surh, 2005). Conversely, Nrf2 small interfering RNA transfection has been observed to inhibit the antioxidant effect of RSV, suggesting that the Nrf2 signaling pathway is one of the key pathways through which RSV exerts its antioxidant effect. Furthermore, RSV is currently also the most thoroughly studied natural SIRT1 activator. RSV significantly upregulates the expression of SIRT1 and PGC-1α proteins in the kidney tissue of DKD rats, increases SOD activity, and downregulates malondialdehyde, ROS, and apoptotic factors, thereby reducing podocyte apoptosis. Additionally, RSV inhibits the excessive synthesis of mtROS and can increase the activity of complexes I and III in the renal cortex of DM mice, reversing the low expression of SIRT1, PGC-1α, and their downstream genes Nrf1 and TFAM (Zhang T. et al., 2019). In summary, RSV ameliorates podocyte damage in diabetic mice by regulating mitochondrial biogenesis and mitochondrial oxidative stress through the SIRT1/PGC-1α signaling pathway. These findings indicate the potential for RSV to serve as an adjunctive treatment for DKD. RSV may be a new and safe method for preventing CKD in the future.

##### 4.1.2.2 Flavonoids

Baicalin, a flavonoid metabolite extracted from *Scutellaria baicalensis Georg*, effectively intervenes in mitochondrial electron transport chain damage, excessive ROS production, and excitotoxicity, thereby protecting mitochondrial function and structure. It demonstrates promising therapeutic effects in various diseases, including neurodegenerative disorders, DM, and its complications (De Oliveira et al., 2015). In DKD mouse models and HG-induced podocyte damage models, baicalin has been shown to inhibit the accumulation of ROS, upregulate Nrf2 and its activator SIRT1 expression, enhance HO-1 protein expression levels, and improve oxidative stress. Simultaneously, baicalin also inhibits the levels of inflammatory factors such as IL-1β, IL-6, MCP-1, and TNFα, intervenes in the MAPK inflammatory signaling pathway, and plays a role in delaying the progression of DKD (Ma et al., 2021).

Calycosin, a flavonoid metabolite isolated from *Astragali Radix*, has been shown to possess antioxidant and neuroprotective properties. In HG-induced HK-2 cells, calycosin has been demonstrated to upregulate GPX4, inhibit lipid ROS, and suppress the expression of nuclear coactivator 2 (NCOA2), thereby enhancing HK-2 cell survival and mitigating cellular damage (Huang D. et al., 2022). Furthermore, calycosin has been demonstrated to possess a potential mechanism for regulating ferroptosis and improving CKD. Liu H. et al. (2023) administered calycosin into MCAO/R rat models and confirmed that calycosin can inhibit ferroptosis. In addition, the treatment of PC12 cells from adrenal medullary pheochromocytoma in OGD/R (oxygen-glucose deprivation/reoxygenation) rats with calycosin has been shown to reduce TFR1 expression, increase ferritin heavy chain peptide 1 and GPX4 levels, and these outcomes are positively correlated with the administered dose. Nonetheless, research on the function of calycosin in addressing mitochondrial dysfunction in CKD remains sparse and necessitates further investigation.

##### 4.1.2.3 Polysaccharides

Angelica sinensis polysaccharides (ASPs) are one of the primary active metabolites in *Angelica sinensis (Oliv.) Diels*. Their pharmacological activities have been reported to include antioxidant and antitumor properties (Nai et al., 2021). In the model of DKD rat, the administration of ASPs has been observed to result in a reduction in the expression of inflammatory factors, including NF-κB, MCP-1, TNF-α, and IL-1, in renal tissue, thereby contributing to the alleviation of renal injury. Moreover, ASPs have been shown to regulate Drp1 protein expression, impede mitochondrial fission in renal cells of DKD mice, and suppress AMPK-mediated excessive mitophagy. This, in turn, serves to restore mitophagy to a dynamic equilibrium, thereby reducing mitochondrial damage. Consequently, it can be deduced that ASPs have the capacity to ameliorate mitochondrial dysfunction in DKD.

Astragalus polysaccharides (APs) are polysaccharide metabolites isolated from *Astragali Radix*, exhibiting anti-inflammatory, antioxidant, and regulatory effects on glucose and lipid metabolism. In HG-induced podocyte damage models, APs can markedly diminish ROS and iron ion content, increase podocyte MMP, increase the expression of proteins such as PGC-1α, GPX4, and SLC7A11, and reduce podocyte apoptosis. Furthermore, APs regulate the AMPK/SIRT1/PGC-1α pathway to preserve mitochondrial integrity, offering a defensive mechanism for renal tubular epithelial cells in DKD. Moreover, APs markedly alleviate kidney damage in DN rats by lowering levels of pro-inflammatory markers such as IL-1β, IL-6, and MCP-1 while suppressing the TLR4/NF-κB signaling pathway (Guo et al., 2023).

##### 4.1.2.4 Other categories

The utilization of Diosgenin (DIO) and its derivatives, derived from the *Dioscorea bulbifera L.*, has been a part of CBD since ancient times (Parama et al., 2020). DIO not only alleviates the decline in HK-2 cell viability and renal pathological damage in DN rats but also inhibits NOX-4 expression, restores the expression of mitochondrial respiratory chain complexes I-V, thereby reducing ROS generation. The NOX family of NADPH oxidases serves as a significant generator of reactive oxygen species, with NOX-4 being the most prevalently expressed in the kidney. It is not only referred to as renal NADPH oxidase (Renox) but also a new therapeutic target for diabetic vascular complications (Geiszt et al., 2000; Wang D. et al., 2023). Therefore, from a mechanistic perspective, DIO has been shown to alleviate DKD by inhibiting NOX-4. Atractylenolide III, a bioactive extract from plants, exhibits notable therapeutic qualities, particularly an anti-inflammatory impact. Research (Wang M. et al., 2019) has demonstrated the efficacy of atractylenolide III in mitigating mitochondrial damage, enhancing antioxidant enzyme activity, curtailing ROS production, and ameliorating renal injury in rats subjected to 5/6 Nx. This protective effect is attributed to the regulation of the PI3K/AKT/mTOR pathway, a process facilitated by mitochondrial oxidative stress. Paeoniflorin (PF) is a significant active metabolite that is extracted from the *Paeonia suffruticosa Andr*. It belongs to the monoterpene metabolite class. The substance exhibits significant effects in anti-inflammatory, antioxidant, and anti-apoptotic activities. Research (Li et al., 2022b) has shown that in instances of CKD, PF activates the AMPK/SIRT1/PGC-1α signaling pathway, thereby inhibiting excessive ROS production and mitochondrial oxidative stress. In addition, PF has been shown to improve MMP and mitochondrial dysfunction, as well as to enhance kidney function and interstitial fibrosis in 5/6 Nx rats.

Research on natural chemical metabolites as mitochondrial antioxidants is abundant, yet it often suffers from a lack of specificity. Compounds like RSV and baicalin are known to activate broad-spectrum cytoprotective pathways (e.g., Nrf2, SIRT1), making it difficult to isolate their antioxidant effects specifically on mitochondria from their general cellular actions. A key limitation is the frequent use of non-specific ROS probes (e.g., DCFH-DA) that do not distinguish mtROS from other cellular sources. Future studies should employ mitochondria-targeted ROS sensors (e.g., MitoSOX) to provide more definitive evidence. Additionally, while reductions in markers like MDA are reported, direct evidence of decreased oxidative damage to mitochondrial proteins, lipids, and mtDNA is often lacking. The therapeutic window for these antioxidants is also poorly defined, as excessive ROS scavenging can disrupt redox signaling.

#### 4.1.3 Natural chemical metabolites improve mitophagy to alleviate CKD

##### 4.1.3.1 Glycosides

*Astragali Radix* is a prevalent botanical drug remedy known for its qi-tonifying and surface-strengthening qualities. Its widespread application in the treatment of chronic nephritis with proteinuria and diabetes is well-documented. Astragaloside II (AS-II), the predominant metabolite of *Astragali Radix*, demonstrated protective advantages for the kidneys. Su et al. (2021) found that after AS-II intervention in the kidneys of DKD rats, there was an increase in autophagy bodies of podocytes and PINK1, Parkin, Fist1 (a protein that binds to the Drp1 protein on the outer mitochondrial membrane to jointly regulate mitochondrial division), MFN2, LC3, mitochondrial outer membrane translocase 20 (TOMM20), and Nrf2 protein expression. The results of the study demonstrated that while the expression of p62 and Keap1 (a regulator of cellular oxidative stress response acting on Nrf2 was suppressed, this finding suggests that AS-II may promote mitophagy and enhance the antioxidant stress capacity of podocytes by activating the Nrf2/PINK1 signaling pathway.

*Corni Fructus* is a botanical drug with the effects of tonifying the liver and kidneys, astringing essence, and consolidating essence. Loganin, as the main metabolite of the cycloartenol terpenoid in *Corni Fructus*, has antioxidant properties (Cheng et al., 2020). *Rehmanniae Radix* is the dried root tuber of *Rehmannia glutinosa*, a plant that is classified within the *Scrophulariaceae* family. Its properties are characterized by a cold nature and a sweet taste, and it is associated with the heart, liver, and kidney meridians. The herb’s therapeutic benefits include the ability to clear heat, cool the blood, nourish yin, and generate fluids. It is widely used in clinical practice. Catalpol, a distinctive metabolite of *Rehmanniae Radix*, has been shown to possess significant antioxidant properties. *In vitro* experiments have demonstrated that loganin and catalpol can inhibit the expression of Nrf2, LC3, PINK1, and Parkin proteins in AGE-induced glomerular mesangial cells, thereby inhibiting the nuclear translocation of Nrf2 protein and the phosphorylation of p62. This mechanism functions by suppressing the Nrf2/PINK1 signaling pathway, thereby reducing mitophagy-induced mesangial cell proliferation. This consequently leads to a decrease in extracellular matrix (ECM) deposition and an enhancement in renal fibrosis and DKD lesions (Bhattamisra et al., 2021; Kong et al., 2023). The above two studies indicate that different monomeric metabolites can target the same mitophagy pathway through distinct regulatory mechanisms, yet both exert renal protective effects in DKD.

##### 4.1.3.2 Terpenoids

*Poria cocos (Schw.) Wolf*, a representative botanical drug known for its diuretic and dampness-draining properties, as well as its ability to tonify qi and strengthen the spleen, is widely used in CBD. Poricoic acid A (PAA) is a metabolite isolated from *P. cocos (Schw.) Wolf cocos* that exhibits hypoglycemic and anti-fibrotic effects. Wu et al. (2023) found that PAA can significantly increase the levels of LC3 and autophagy-related protein 5 in podocytes MPC5 and CKD mouse kidney tissue treated with high sugar, while reducing the levels of p62 and FUNDC1, promoting mitophagy activation, thereby exerting a beneficial effect on DKD podocyte damage.

Jujuboside A (JuA) is a triterpene saponin isolated from the mature seeds of the *Ziziphus jujuba Mill.* Modern pharmacological research has shown that it has biological activities such as hypnotic sedation, antidepressant, lipid regulation, and blood pressure reduction. As demonstrated by Zhong et al. (2022), JuA has been found to activate the PINK1/Parkin signaling pathway, thereby increasing the level of mitophagy in the kidney tissue of DKD rats. This activation is achieved through the upregulation of the protein expression of LC3II and Parkin, the colocalization of LC3 and PINK1, and the reduction of the expression of p62 in the kidney tissue of DKD rats. Furthermore, JuA downregulated the expression of p-IRE1, p-ERK, XBP1, p-CHOP, caspase 12, and ATF4 to inhibit endoplasmic reticulum-induced oxidative stress and apoptosis, thereby alleviating renal pathological changes in rats.

Demonstrating a direct and specific enhancement of mitophagy, as opposed to general autophagy, is technically challenging. Many studies infer mitophagy from changes in the protein levels of PINK1, Parkin, and LC3-II, or from the colocalization of mitochondrial and autophagosomal markers by microscopy. However, these are indirect measures. More rigorous methodologies, such as mitophagy reporter assays (e.g., mt-Keima) or quantitative assessment of mitochondrial protein degradation, are needed to confirm true mitophagic flux. Another concern is the potential for (excessive activation) of mitophagy, which could lead to detrimental mitochondrial loss—an aspect rarely explored in these studies. The findings that different metabolites (e.g., AS-II vs. loganin/catalpol) can target the same Nrf2/PINK1 pathway yet have seemingly opposing effects on mitophagy in different cell types underscore the context-dependency of these responses and the complexity of therapeutic targeting.

#### 4.1.4 Natural chemical metabolites improve mitochondrial dynamics to alleviate CKD

##### 4.1.4.1 Polyphenols

Green tea is obtained from the leaves of plants and is a popular beverage among the general public. Green tea comprises a category of natural catechin chemicals, notably epigallocatechin gallate (EGCG), a polyphenol that demonstrates considerable anti-inflammatory and antioxidant effects. Research (Alam et al., 2022; Cao et al., 2016) indicates that EGCG can enhance cognitive function and has inhibitory effects on abnormal modifications in the human body, including antioxidant, anti-inflammatory, anti-cancer, and anti-angiogenic properties, as well as protective activity against diseases caused by oxidative stress and inflammation. Chu et al. (2024) investigated the effects of EGCG on energy metabolism and mitochondrial dynamics in a mouse model of kidney injury. The study revealed that EGCG diminished pathological alterations in renal tissue by reducing SCr and BUN levels. Additionally, EGCG treatment significantly improved changes in ROS, malondialdehyde content, and antioxidant enzyme activity. Furthermore, the EGCG-induced renal protective effects were found to be associated with increased MMP and reduced mitochondrial Drp1. Consequently, EGCG has demonstrated protective effects in kidney injury by curbing oxidative damage, metabolic disturbances, and mitochondrial dysfunction, highlighting its promise as a viable strategy for preventing renal damage.

##### 4.1.4.2 Glycosides

*Epimedii Folium* is among the most often utilized CBD. It is an important metabolite in Chinese patent medicines and health supplements. Modern research (Bi et al., 2022) has shown that *Epimedii Folium* is primarily utilized to tonify kidney yang, strengthen tendons and bones, and dispel wind-dampness, and it has significant therapeutic effects. Its main active metabolite, icariin (ICA), has multiple pharmacological effects, including neuroprotection, anti-inflammation, and antioxidant properties (Zhang X. et al., 2023). Numerous studies (Jia Z. et al., 2021; Wang K. et al., 2020; Zhang et al., 2017) have shown that ICA significantly contributes to the enhancement of DKD and the preservation of renal function. ICA may treat CKD by targeting mitochondrial dynamics. Wang et al. (2022a) employed a rat model of CKD induced by 5/6Nx and infarction (A/I) surgery, followed by 8 weeks of treatment with ICA. The findings indicated that rats with high-dose ICA had notable enhancements in fibrosis, mitochondrial dynamics, and mitochondrial function. Additionally, *in vitro* experiments revealed that ICA inhibits the increase in CTGF and p-Drp1 Ser616 in a dose-dependent manner, while increasing p-Drp1 Ser637, MFN1, MFN2, TFAM, and ATP6. This leads to increased ATP levels and enhanced mitochondrial structure in TGF-β1-stimulated NRK-52E cells. In contrast to ICA monotherapy, the combination of ICA and MFP-M1 further diminished the expression of CTGF in TGF-β1-stimulated NRK-52E cells. In conclusion, ICA attenuates CRF-induced renal interstitial fibrosis (RIF) by improving mitochondrial dynamics.

##### 4.1.4.3 Flavonoids

Formononetin (FMN) is a phytoestrogen and a member of the flavonoid family. It is one of the active metabolites in commonly used CBDs, including *Puerariae Lobatae Radix, Astragali Radix,* and *Callerya reticulata (Benth.) Schot*. FMN exhibits various pharmacological effects, including antioxidant, antihypertensive, anti-tumor, anti-infective, and estrogen-like properties (Jin et al., 2025). Studies have found (Zhu et al., 2023) that FMN can relieve renal tubular damage and improve renal function in CKD models by inhibiting ferroptosis. In DKD, FMN partially addresses mitochondrial kinetics to manage DKD. Huang Q. et al. (2022) performed research utilizing STZ-induced diabetic rats to investigate the therapeutic benefits of FMN on DKD. The treatment with FMN reduced programmed cell death in renal tubular cells and prevented mitochondrial fragmentation, concurrently restoring levels of key proteins involved in mitochondrial dynamics (Drp1, Fis1, MFN2) as well as apoptotic regulators (Bax, Bcl-2, Caspase-3). Additionally, FMN upregulated the renal expression of SIRT1 and PGC-1α in the diabetic model. FMN also reduced mitochondrial superoxide production and alleviated MMP loss. In summary, FMN may alleviate renal tubular damage and mitochondrial damage in DN by regulating the SIRT1/PGC-1α signaling pathway.

Silibinin (SB) is a polyphenolic flavonoid metabolite extracted from the dried fruit of *Silybum marianum (L.) Gaertn.* and has a variety of pharmacological activities, including anti-oxidant, anti-inflammatory, and apoptosis inhibition. From a clinical perspective, the utilization of this substance is prevalent in the therapeutic management of hepatic diseases (Selc et al., 2024). Recent research has increasingly shown that SB is significant in the management of kidney disease. SB can suppress the ROS/MAPK signaling pathway by activating the Nfe2l1-mediated antioxidant response, thereby ameliorating cisplatin-induced AKI (Yang et al., 2022). There are also studies (Li Y. et al., 2017) showing that SB improves mitochondrial function by regulating SIRT3 expression, thereby preventing cisplatin-induced AKI. In addition, in HFD-induced renal fibrosis, SB significantly reversed TGF-β1-induced increases in the expression of type I collagen, fibronectin, α-SMA, p-IκB, and p-p65, and reduced NF-κB levels. Furthermore, the study by You et al. (2020) demonstrated that SB promotes G1/S transition by activating Drp1, thereby mediating mitochondrial fission in these normal cells and significantly promoting cell proliferation. SB dose-dependently increases mitochondrial mass, mtDNA copy number, cellular ATP production, MMP, and ROS in normal cells. Moreover, SB has been observed to increase Drp1 expression in a dose-dependent manner, and the inhibition of Drp1 has been shown to result in the elimination of SB-induced mitochondrial fission.

##### 4.1.4.4 Terpenoids

Asiatic acid (AA), a terpenoid metabolite extracted from *Centella asiatica (L.) Urban*, has antioxidant and immunomodulatory properties (Liang et al., 2025). Despite the evidence that AA exerts renoprotective effects in DKD, the therapeutic effects of AA on tubular damage remain to be elucidated, a fact that has generated considerable interest among researchers. To elucidate the effects of AA on DKD tubular injury and its mechanisms, Ji et al. (2023) treated STZ-induced diabetic rat tubules and AGEs-stimulated HK-2 cells with AA. The results demonstrated that AA exhibited excellent protective effects on the tubules of STZ-induced diabetic rats and of HK-2 cells stimulated with AGEs. A significant reduction in albumin, urinary KIM-1, SCr, and BUN levels was observed. In addition, the impact of Asiatic acid on mitochondrial dynamics and tubular protection was negated following treatment with ML385 (Nrf2 inhibitor), indicating that AA could be a promising pharmaceutical agent for the management of DKD tubular damage. Its mechanism of action may involve the modulation of the Nrf2 pathway and mitochondrial dynamics.

##### 4.1.4.5 Other categories

*Chuanxiong Rhizoma* is a typical CBD for promoting blood circulation and removing blood stasis. Tetramethylpyrazine (TMP), as the most important alkaloid active substance in *Chuanxiong Rhizoma*, is a new calcium ion antagonist that has the effects of improving microcirculation, preventing thrombosis, improving renal ischemia, and protecting renal function (Chen et al., 2025; Sue et al., 2009). Gong et al. (2019) investigated the therapeutic effects of TMP on a rat model of contrast-induced nephropathy (CIN) and its underlying mechanisms. They measured renal function, urinary AKI biomarkers, and renal ROS production in the rats. The study showed that CIN rats experienced higher levels of mitophagy, mitochondrial fragmentation, ROS generation, autophagy, and apoptosis within renal tubular cells. Notably, TMP largely blocked contrast-enhanced kidney injury by reversing these detrimental changes. Mechanistically, TMP suppressed CM-triggered activation of the CCL2/CCR2 axis, mitigated renal OS and irregular mitochondrial dynamics, and modulated mitophagy in renal tubular cells. Tetramethylpyrazine nitrone (TBN) represents a novel nitrone derivative derived from TMP. Jing et al. (2021) found that TBN can improve mitochondrial function in a DN rodent model by activating the AMPK/PGC-1α signaling pathway, reducing mitochondrial oxidative stress, and improving renal tissue pathological changes. The above study indicates that TMP is a monomeric metabolite that protects kidney function by targeting MQC. *Fructus Schisandrae Chinensis* is a CBD used to tonify qi and nourish the kidneys. Modern pharmacological research has shown that the efficacy of *Fructus Schisandrae Chinensis* from lignan metabolites, with Schisandrin B (Sch B) being the most abundant. Sch B plays a significant role in liver protection, anti-oxidation, anti-aging, and other aspects (Yan et al., 2022). Numerous studies (Jiang et al., 2015) have shown that Sch B can elevate intracellular SOD levels, while inhibiting lipid peroxidation (LPO), reducing the release of LDH, MDA, and ROS, and directly scavenging free radicals to exert an antioxidant effect. Several studies have shown that Sch B plays an important role in the treatment of CKD. Liu W. et al. (2023) revealed that Sch B attenuated renal tubular EMT and mitochondrial dysfunction in db/db mice, accompanied by the downregulation of TGF-β1 and upregulation of PGC-1α. Similarly, Sch B exhibited a protective effect by reducing the expression of TGF-β1, α-SMA, fibronectin, and Col-I, while increasing the expression of E-cadherin in high-glucose-stimulated HK-2 cells. Furthermore, Sch B was demonstrated to enhance MMP, reduce ROS production, and elevate ATP levels in HK-2 cells subjected to high glucose conditions, alongside the overexpression of PGC-1α, TFAM, MFN1, and MFN2. These findings indicate that Sch B can enhance KCP expression by inhibiting the Akt pathway and activating the AMPK pathway in DKD, consequently mitigating EMT and mitochondrial dysfunction in HK-2 cells.

Studies in this section convincingly show that natural chemical metabolites can shift the balance of mitochondrial dynamics proteins (e.g., reducing Drp1, increasing MFN2). A significant limitation, however, is the primary reliance on protein expression data. Functional assessments of mitochondrial networking, such as live-cell imaging to quantify fission and fusion rates, are less common and would provide more dynamic and physiologically relevant evidence. Furthermore, it is often unclear whether the observed changes in dynamics are a primary effect of the metabolite or a secondary consequence of improved overall mitochondrial health and bioenergetics. The use of genetic models (e.g., Drp1 knockdown/overexpression) in conjunction with metabolite treatment could help establish causal relationships.

#### 4.1.5 Natural chemical metabolites improve mitochondrial apoptosis to alleviate CKD

##### 4.1.5.1 Glycosides

*Panax notoginseng (Burkill) F.H.Chen*, a CBD, has been shown to promote blood circulation and alleviate pain. The primary function is to address symptoms, including hematemesis, hemoptysis, and external hemorrhage. Panax notoginsenosides are the main active metabolites of *P. notoginseng (Burkill) F.H.Chen*, among which Notoginsenoside R1 (NGR1), as a phytoestrogen, can regulate the AKT/Nrf2 or TNF-α pathway through estrogen receptors (Zhong et al., 2015). NGR1 has a good regulatory and protective effect on multiple systems, organs, and tissues, and can prevent and treat a variety of diseases, so it has broad prospects for clinical application. It has been reported that NGR1 has renoprotective, antidiabetic, and hepatoprotective effects (Feng et al., 2024). In DKD, Zhang B. et al. (2019) demonstrated that NGR1 alleviates histological abnormalities in diabetic kidneys, such as reduced renal fibrosis. *In vitro*, NGR1 also reduces AGE-induced mitochondrial damage, limits the increase in ROS, and reduces apoptosis in HK-2 cells. Mechanistically, NGR1 promotes the expression of Nrf2 and HO-1 in the cell nucleus, thereby eliminating ROS that induce apoptosis and TGF-β signaling. In addition, several studies have explored the effects of NGR1 on DKD-induced podocyte damage. Huang et al. (2016) found that NGR1 improved renal function by improving histological changes, increasing renin and podocin expression, reducing desmin expression, and suppressing inflammatory responses and podocyte apoptosis. In their further study (Huang et al., 2017), they revealed that NGR1 protects podocytes from HG-induced damage by reducing apoptosis, increasing autophagy, and promoting cytoskeletal recovery, and can protect podocytes via activation of the PI3K/Akt/mTOR pathway. These findings highlight the multi-targeted renal protective role of NGR1, modulating oxidative stress, mitochondrial function, and podocyte homeostasis in DKD.

*Corni Fructus* was first recorded in Shennong Bencao Jing (The Divine Farmer’s Classic of Materia Medica) and is commonly used in clinical practice for its ability to nourish the liver and kidneys and astringe and consolidate. Cornuside (Cor) is a cycloartenol glycoside metabolite extracted from the fruit of Corni Fructus. It is a warm tonic that has anti-inflammatory, antioxidant, and anti-apoptotic effects (Gao et al., 2021). Numerous studies (Ma et al., 2014; Xiang et al., 2025) highlight the substantial involvement of Cor in managing CKD. Voltage-dependent anion channel 1 (VDAC1) is an OMM protein that acts as a mitochondrial regulator, controlling the transport of metabolites in and out of mitochondria and energy production, while coordinating glycolysis and OXPHOS. VDAC1 is crucial in mitochondria-driven apoptosis, functioning by releasing apoptotic proteins from the intermembrane space and engaging with both pro-apoptotic and anti-apoptotic proteins. Gao et al. (2025) found that Cor significantly improved kidney function and reduced kidney pathology in db/db mice. Its effects arise from dampening the PERK/ATF4/CHOP signaling axis and reducing VDAC1 expression, which in turn mitigates mitochondrial calcium overload and limits cellular apoptosis. Existing research indicates that Cor holds therapeutic potential for CKD, primarily by protecting mitochondrial function and inhibiting the PERK/ATF4/CHOP-VDAC1 apoptotic pathway. However, further validation is required to determine its precise molecular targets, long-term efficacy, and clinical translation feasibility.

##### 4.1.5.2 Flavonoids

Mangiferin (MGF), a flavonoid present in many medicinal plants, has a range of pharmacological actions, including lowering lipids and blood glucose, protecting the liver, and exerting anti-apoptotic and anti-inflammatory effects. Due to its high safety profile, it is widely used in clinical settings (Chen et al., 2021). It has been found (Pal et al., 2014) that MGF plays a crucial role in reducing the production of mitochondrial reactive oxygen species (mtROS). Hexokinase 2 (HK-2) mainly resides on the OMM, and its detachment from the mitochondrial surface can prompt the generation of mtROS. MGF helps maintain HK-2’s connection to the membrane, reducing its likelihood of dissociation. Both *in vitro* and *in vivo* studies indicate that MGF can suppress NOX-4 expression in the kidneys of diabetic mice. Additionally, Research revealed that MGF effectively reduces ROS and O^2-^ levels in kidney tissue while boosting the diminished activity of key antioxidant enzymes, including CAT, SOD, and GPx. When it comes to the origins of OS, AGEs, and xanthine oxidase play a major role as primary drivers of ROS production in diabetic states. In this study, MGF inhibited AGEs formation and xanthine oxidase activity. However, mtROS was not directly measured in this experiment, so a more specific method is needed to determine the source of ROS. Building on this, Guang-Kai et al. (2017) further revealed that MGF effectively reduces oxidative stress, caspase-3 expression, and apoptosis by limiting the collapse of the MMP. This indicates that MGF has a protective effect against diabetes-induced kidney damage and prevents OS in interstitial cells by regulating the binding of hexokinase II and the signaling of NOX-4 oxidase.

##### 4.1.5.3 Other categories

*Salvia miltiorrhiza Bunge* has many effects, such as improving vascular endothelial function, maintaining endothelial integrity, and reducing the production of vasoactive factors. Its protective effect on the kidneys is currently being confirmed by research (Shen Z. et al., 2023). Salvianolic acid B (Sal B) is the most potent pharmacologically active metabolite of *S. miltiorrhiza Bunge*. It has been found to have cardioprotective and renoprotective effects, as well as antioxidant, anti-inflammatory, and anti-apoptotic properties. RIF is a central pathological hallmark of end-stage renal disease (ESRD) arising from various CKD and is closely associated with diminished renal function and patient prognosis. PDGF-C is a pro-inflammatory factor that mediates renal interstitial fibrosis, acting through binding to PDGFR-α. In an UUO rat models, the PDGF-C/PDGFR-α signaling pathway is activated, and PDGF-C is overexpressed in fibrotic regions. Antagonizing PDGF-C or knocking out the PDGF-C gene in UUO rats significantly reduces renal fibrotic damage (Eitner et al., 2008; LeBleu and Kalluri, 2011). Thus, inhibiting the PDGF-C/PDGFR-α signaling pathway represents a novel approach for preventing and treating renal fibrosis. Yao et al. (2022) investigated the precise molecular mechanisms underlying the renal protective effects of Sal B *in vivo* and *in vitro*. The results showed that *in vivo*, Sal B partially improved renal dysfunction, increased Par-3 expression, and reduced CTGF, PDGF-C, and PDGFR-α expression. *In vitro*, Sal-B reversed HSA-induced apoptosis and ERS in HK-2 cells by regulating the PDGF-C/PDGFR-α signaling pathway. Additionally, Sal-B is crucial in the management of DKD. Wen et al. (2023) assessed renal tissue pathological changes using TUNEL staining and evaluated renal function through biochemical testing. Additionally, Sal B also alleviated renal tissue pathological changes, function, and apoptosis in CKD rats, partially achieved through activation of the SIRT3/FOXO1 signaling pathway. These studies demonstrate that Sal B improves CKD by inhibiting apoptosis.

The anti-apoptotic effects of natural chemical metabolites are frequently demonstrated through reductions in caspase activity and shifts in the Bax/Bcl-2 ratio. While these are standard assays, a common shortcoming is the failure to definitively link these changes to the specific intrinsic (mitochondrial) apoptotic pathway. Measurements of key events like mitochondrial outer membrane permeabilization (MOMP), precise cytochrome c release kinetics from mitochondria (not just total cellular levels), and the assembly of the apoptosome would provide more mechanistic depth. Additionally, many studies do not adequately distinguish between anti-apoptotic effects and general pro-survival or cytoprotective effects, which could be mediated through unrelated mechanisms.

#### 4.1.6 Natural chemical metabolites improve the gut-kidney axis to alleviate CKD

The *phylum Proteobacteria*, which includes many important opportunistic pathogens, exhibits low abundance in the normal gut (below 4%), and its expansion is considered a marker of gut microbiota dysbiosis (Shin et al., 2015). In rats with CKD, a significant increase in the abundance of *Enterobacteriaceae*—a family responsible for generating uremic toxins such as IS and PCS—has been observed (Chen et al., 2020). Colonic infusion of berberine (Zeng et al., 2016) and APs (Hui et al., 2020) markedly reduced *Escherichia coli* counts, a species positively correlated with urea and IS levels. Furthermore, in kidney-injured mice, the relative abundances of *E. coli* and *Shigella* within the *Enterobacteriaceae* family were significantly elevated, while curcumin intervention restored these levels to normal (Xu et al., 2021).

In CKD animal models, the relative abundance of bacteria producing SCFAs is significantly reduced, including *Lactobacillus*, *Akkermansia, Bacteroides,* and *Ruminococcus. Lactobacillus*, a lactic acid-producing probiotic, has been observed to decrease in CKD patients and various CKD models (Nagayama et al., 2020). Increasing their abundance can protect renal function by reducing the accumulation of uremic toxins (e.g., IS), repairing the intestinal barrier, and improving inflammation and OS (Tungsanga et al., 2022). Existing studies (Zhang et al., 2022; Yang et al., 2020) have demonstrated that Moutan Cortex polysaccharide and Cordyceps cicadae polysaccharide not only restore *Lactobacillus* levels but also significantly enhance the abundance of *Akkermansia*, a beneficial gut microorganism crucial for maintaining intestinal integrity, in chronic kidney toxicity models. Furthermore, *Pseudomonas mucinosa* delays the onset of diabetes, obesity, and inflammatory bowel disease in mice, while total flavones from *Abelmoschus manihot* similarly enrich this bacterial species in CRF rat models (Tu et al., 2020).

Several studies (Davar et al., 2021; Zhao et al., 2019) have demonstrated that the *Bacteroides genus* is associated with enhanced immune responses, improved renal function, and primary bile acid synthesis. In CKD models, reduced *Bacteroides* were significantly increased following treatment with emodin nanoparticles and administration of Dioscorea polysaccharides (Lu Z. et al., 2021). The acetic acid-producing taxa *Lachnospiraceae* UCG-001 and the *Lachnospiraceae* NK4A136 group were significantly reduced in CKD rats but significantly elevated after intervention with docosahexaenoic acid-acylated curcumin diester or curcumin (Shi et al., 2022). Moreover, studies (Tu et al., 2020) have observed that treatment with total flavones from *A. manihot* significantly increased *Lactobacillus* species in potassium oxide-induced CRF rat models. Collectively, these findings suggest that these monomeric metabolites enhance the dominance of beneficial gut bacterial species.

Gut microbiota dysbiosis in CKD leads to increased intestinal barrier permeability, allowing pathogenic bacteria or endotoxins (such as LPS) to traverse intestinal epithelial cells and enter the bloodstream and lymphatic system. Therefore, maintaining the integrity and function of the intestinal epithelial barrier holds therapeutic significance for CKD. Tight junction proteins (including claudin-1, desmoglein-1, and occludin) are indispensable for maintaining intestinal epithelial barrier permeability. Decreased expression of these proteins leads to increased paracellular permeability, disruption of tight junction structures, and ultimately impaired intestinal barrier function (Paone and Cani, 2020). Reduced expression of tight junction proteins has been detected across multiple renal disease models. Notably, treatment with astragalus polysaccharides (Yang et al., 2021) restored intestinal epithelial tight junctions, reduced intestinal permeability, and upregulated ZO-1, occludin, and claudin-1 expression in the 5/6 Nx rat model. Furthermore, studies demonstrate that resveratrol (Cai et al., 2020) and curcumin supplementation (Xu et al., 2021) significantly restore tight junction protein-1/2 expression in CKD models, effectively protecting the intestinal epithelial barrier. Thus, utilizing plant-derived natural products to improve intestinal barrier function can effectively alleviate intestinal inflammation and renal fibrosis.

The evidence that natural chemical metabolites can modulate gut microbiota and barrier function is intriguing but largely associative. A major limitation is that most studies demonstrate correlation rather than causation. It remains to be proven whether the renal benefits of, for example, curcumin or astragalus polysaccharides, are directly mediated by the observed shifts in gut microbial populations. Fecal microbiota transplantation (FMT) experiments from treated to untreated animals would be a critical next step to establish causality. Furthermore, the field is hampered by a focus on relative abundance changes in microbiota, which can be misleading, and a lack of integrated multi-omics approaches to link specific bacterial changes to metabolite production and subsequent host mitochondrial phenotypes.

In summary, while the aforementioned studies suggest promising effects of natural metabolites on MQC in CKD, several limitations must be acknowledged. First, many experiments use high-dose treatments that may not reflect physiologically achievable concentrations. Second, the specificity of these compounds for mitochondrial targets is often inferred rather than demonstrated. For example, the effects of curcumin and resveratrol are pleiotropic, involving multiple signaling pathways beyond MQC. Furthermore, the lack of standardized extraction methods and metabolite characterization across studies complicates cross-study comparisons. Future research should prioritize dose-response studies, target validation using genetic or pharmacological inhibitors, and assessments of bioavailability and tissue distribution.

**FIGURE 4:**
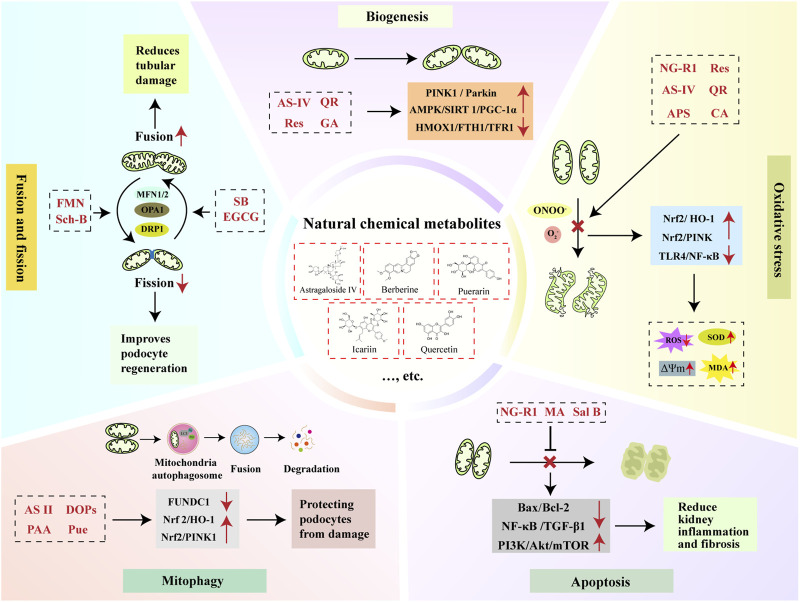
Schematic diagram of natural chemical metabolites improving CKD by regulating MQC MQC-targeted natural chemical metabolites include RES, QR, and GA, which improve mitochondrial function through multiple pathways, thereby alleviating CKD. Abbreviations: RES: resveratrol; QR: quercetin; GA: glycyrrhizic acid; CA: chicoric acid; NG-R1: notoginsenoside R1; APS: astragalus polysaccharide, Pue: puerarin; AS-II: astragaloside II; PAA: poricoic acid A; SB: silybin; AA: asiatic acid; FMN: formononetin; MA: mangiferin; Sal B: salvianolic acid B.

The mechanisms of action and results of the relevant natural chemical metabolites are summarized in Supplementary Table S1 and shown in Figure 4.

### 4.2 CBD formulations improve MQC to alleviate CKD

In contrast to isolated metabolites, CBD formulations represent a cornerstone of TCM therapy. These formulations, comprising multiple herbs based on TCM theory to achieve synergistic effects and reduce toxicity, offer a unique approach. The following section will explore how CBD formulations improve CKD by regulating the MQC system.

#### 4.2.1 CBD formulations improve mitochondrial oxidative stress to alleviate CKD

Nourishing yin and promoting blood flow recipe (NYPBR) is a decoction of CBD consisting of *Rehmannia glutinosa (Gaetn.) Libosch. ex Fisch. et Mey., Yam, Corni Fructus*, and *Moutan Cortex*. Succinate dehydrogenase (SDH) sits in the IMM and participates in the tricarboxylic acid cycle. Researchers have employed SDH staining techniques to gauge the oxidative power of these energy-packed organelles (Jang et al., 2022). Li et al. (2009) reported that in an STZ-induced DKD rat model, NYPBR significantly reduced mitochondrial oxidative stress in the kidneys of DKD rats, reversed the decline in SDH, Na^+^-K^+^-ATPase, Ca^2+^-Mg^2+^-ATPase, and SOD activity, and improved mitochondrial cristae and membrane disorganization. Additionally, NYPBR reduced iNOS mRNA expression and protein levels, decreased excessive ONOO- formation, and protected the aortic endothelium of diabetic rats from damage. In summary, current evidence suggests NYPBR may slow and mitigate DKD progression by enhancing mitochondrial oxidative stress resistance.

Liu Wei Di Huang Wan (LWDHW), formulated with *Rehmanniaglutinosa (Gaertn.) Libosch. ex Fisch. and C. A. Mey., Moutan Cortex, Yam*, etc., is a representative prescription for the treatment of early DKD with liver and kidney yin deficiency syndrome. In HG-induced HK-2 cells, LWDHW markedly reduces mitochondrial ROS generation, enhances MMP and mtDNA copy number, prevents apoptosis in HK-2 cells, diminishes OS, and slows the advancement of interstitial fibrosis in DKD. In addition, LWDHW can also downregulate the expression of NF-κB, MCP-1, and TGF-β in DKD rats, and reduce inflammatory damage in DKD kidneys by mediating NF-κB and other signaling pathways. These studies (Xie et al., 2023; Xu et al., 2017; Zhu et al., 2024) indicate that the important mechanism of LWDHW in the prevention and treatment of DKD is closely related to improving mitochondrial dysfunction by enhancing antioxidant stress capacity and inhibiting inflammation.

Shenqi Dihuang Decoction (SQDHD) is a classical formula comprising *Astragali Radix* and *R. glutinosa (Gaetn.) Libosch. ex Fisch. et Mey.* etc., with properties that include tonifying qi, nourishing yin, and clearing heat. It is a representative prescription for the mid-term differentiation and treatment of DKD deficiency syndrome of both qi and yin. Wang Z.-B. et al. (2023) revealed that SQDHD can significantly reduce the levels of Fe^2+^, MDA, GSH, and ROS in DKD mice and HG-damaged HK-2 cells, increase the expression of key iron death proteins such as GPX4 and SLC7A11 in the kidneys of DKD mice, and increase the expression of Nrf2 and HO-1 proteins in HK-2 cells, thereby changing glomerular and tubular lesions, reducing urine protein, and alleviating DKD renal fibrosis. The important mechanism of SQDHD in preventing and treating DKD is related to its antioxidant and anti-oxidant effects and activation of the Nrf2/HO-1/GPX4 signal axis to inhibit ferroptosis. However, there are few studies on its intervention in DKD mitochondrial dysfunction, which urgently needs to be explored in depth.

Zhenwu Decoction (ZWD) is composed of *Panax notoginseng (Burkill) F.H.Chen, Aconiti Lateralis Radix Praeparata, Poria cocos (Schw.) Wolf, and Paeonia lactiflora Pall.* It has the effects of warming and replenishing kidney yang, transforming qi, and activating water. It is a representative prescription for the differentiation and treatment of kidney yang weakening syndrome in advanced DKD. In a mouse model of DKD with spleen and kidney deficiency, ZWD has been shown to regulate the Nrf2/HO-1/GPX4 signaling pathway, thereby improving oxidative damage and pathological changes in the kidneys. In addition, ZWD has been demonstrated to reduce the expression of NF-κB in the kidneys and to downregulate the levels of serum inflammatory factors such as IL-1β, IL-6, IL-8, and TNF-α, thus protecting kidney function (Jin et al., 2023; Zhao Z. et al., 2024). Furthermore, ZWD significantly reduced ROS levels in HK-2 cells co-treated with hypoxia and lipopolysaccharide, increased the expression of PGC-1α, Nrf1, and Nrf2 proteins, and downregulated the expression of hypoxia-inducible factor-1α, resulting in increased mitochondrial numbers, reduced mitochondrial pathological changes, and decreased apoptosis. These results suggest that ZWD can improve mitochondrial biogenesis by inhibiting oxidative stress responses, hypoxia responses, and inflammatory responses, thereby slowing the progression of advanced DKD.

Dang Gui Bu Xue Decoction (DGBXD) is a traditional Chinese herbal formula composed of *Astragali Radix, Angelica archangelica L., Cinnamomum cassia (L.) D. Don*, and other herbs are an excellent treatment for DKD characterized by both qi and blood deficiency. DGBXD has been demonstrated to significantly increase the SOD activity and MFN2 and PCG-1α expression of podocytes in DKD rats, reduce ROS production and the expression of inflammatory factors such as IL-1β and Drp1, and significantly improve mitochondrial swelling, mitochondrial cristae disruption, and vacuolation (Qiang et al., 2022; Sun L. et al., 2022). In addition, after DGBXD intervention, HG-induced autophagy marker protein LC3II expression increased, mTOR expression decreased, and apoptosis decreased in podocytes. These studies suggest that the mechanism of DGBXD in the prevention and treatment of DKD is closely related to the inhibition of podocytes’ mitochondrial division and apoptosis, the alleviation of podocytes’ OS, and the reduction of inflammatory responses.

#### 4.2.2 CBD formulations improve mitochondrial biosynthesis to alleviate CKD

Xiaoyu Xiezhuo Decoction (XYXZD) is a combination of various botanical metabolites, including *Astragali Radix* and *Codonopsis pilosula (Franch.) Nannf.*, primarily used for kidney qi deficiency with stagnation of blood and turbidity obstructing the flow of qi and blood, accompanied by mild damp-heat. A mounting body of research (Ji et al., 2024) has indicated the efficacy of XYXZD in the management of kidney diseases. Research has demonstrated that XYXZD effectively lowers urinary protein levels in DKD mouse models while enhancing renal function, mitigating structural kidney damage, and curbing podocyte cell death through modulation of the HIF-1α signaling pathway. Clinical trial data further support these findings, revealing that DN patients treated with XYXZD exhibited decreased serum leptin concentrations alongside noticeable improvements in both symptoms and kidney performance. Additionally, HIF-1α has been shown to ameliorate mitochondrial dysfunction in DN tubular cells by activating HO-1, thereby reducing apoptosis triggered by mitochondrial pathways. It further improves tubular damage in DN by controlling mitochondrial dynamics through HO-1-mediated mechanisms. Additionally, HIF-1α alleviates high-glucose-induced tubular cell damage by promoting mitosis mediated by PINK1/Parkin (Jiang et al., 2020).

#### 4.2.3 CBD formulations improve mitochondrial dynamics to alleviate CKD

Shenshuai II Recipe (SSR) mainly consists of *Codonopsis Radix, Epimedii Folium*, etc. The whole recipe nourishes both the spleen and kidneys, treats both cold and heat, nourishes and drains, and regulates qi and blood. While nourishing, it also transforms stasis and drains turbidity, treating both the symptoms and the root cause with remarkable efficacy. Therefore, the formula demonstrates notable clinical efficacy in the treatment of CKD. Wang et al. (Wang et al., 2022b) investigated the renal protective mechanisms of SSR *in vivo* and *in vitro* by establishing an A/I surgery-induced CKD rat model and using HK-2 cells *in vitro*. They administered SSR treatment to the 5/6 A/I surgery-induced CKD model and treated HK-2 cells in a hypoxic environment with SSR under the condition of the mitochondrial fusion promoter M1. The results showed that SSR treatment significantly improved the mitochondrial morphology and function of HK-2 cells subjected to 5/6 Nx, upregulated PGC-1α protein expression, and inhibited mtROS production, which may be closely related to its anti-RIF effects. In HK-2 cells with PGC-1α knockdown, the effects of SSR on improving mitochondrial dynamics and energy metabolism were significantly weakened. Therefore, SSR may improve mitochondrial dynamics under hypoxic conditions by activating PGC-1α, thereby exerting a renal protective effect. Additionally, studies have shown (Wang L. et al., 2023) that after 8 weeks of SSR treatment in a model of 5/6 Nx rats and SSR treatment in NRK-52E cells subjected to hypoxia *in vitro*, SSR significantly alleviated abnormal glycolysis both *in vivo* and *in vitro*, which is associated with its renal protective effects. Further studies suggest that improving mitochondrial dynamics may be one of the mechanisms by which SSR inhibits glycolysis to achieve its anti-renal fibrotic effects.

Jian-Pi-Yi-Shen formula (JPYSF) is a classic formula containing *Astragali Radix, Yam,* and *Atractylodes macrocephala Koidz.*, etc. It has the effects of tonifying the kidney and supporting the spleen, nourishing blood, and replenishing qi. JPYSF exhibits significant therapeutic effects on kidney diseases, effectively alleviating edema symptoms in patients with CKD and reducing sodium and water retention (Gao et al., 2023; Li C. et al., 2023; Li et al., 2025). Liu et al. (2021) established a CKD rat model using the 5/6 Nx and an adenine-containing diet to investigate the efficacy of JPYSF in treating CKD and to explore its potential mechanisms. They found that in both CKD rat models, administration of JPYSF significantly reduced Scr and BUN levels, improved tubular atrophy and interstitial fibrosis, and reduced extracellular matrix deposition in the kidneys. Additionally, CKD rats exhibited inhibition of the QPRT/NAD/SIRT3 signaling pathway, increased mitochondrial fission, and reduced mitochondrial fusion. JPYSF treatment promoted QPRT/NAD/SIRT3 signaling pathway and restored mitochondrial fission/fusion balance. Furthermore, Gao et al. (Gao et al., 2023) investigated the effects of JPYSF on the progression of renal fibrosis in CKD and found that JPYSF restored the expression of key enzymes involved in NAD^+^ biosynthesis, including pyrimidine phosphate ribosyltransferase and nicotinamide nucleotide adenylyltransferase 1 (NMNAT1), thereby rescuing the decline in NAD^+^ levels in CKD mice and TGF-β1-induced HK-2 cells. These studies collectively highlight the important role of JPYSF in targeting mitochondrial dynamics for the treatment of CKD.

Jiangya Tongluo Decoction (JYTLD) consists of *Margaritifera Concha, Chrysanthemum L.,* and *Scutellariae Radix,* etc. It is usually used to calm the liver and reduce yang qi, while also promoting blood flow to relieve meridian obstruction. Previous studies (Han et al., 2015) have suggested that JYTLD may help alleviate kidney damage caused by hypertension; however, its potential mechanisms have not been thoroughly evaluated. Therefore, Zhao Y. et al. (2024) used spontaneously hypertensive rats (SHRs) and Wistar-Kyoto rats (WKYs) to evaluate the efficacy of JYTLD on hypertensive nephropathy (HN). The results showed that JYTLD improved renal function by inhibiting ROS production and regulating mitochondrial dynamics, alleviating renal tubulointerstitial fibrosis (TIF), and enhancing mitochondrial function. JYTLD treatment also increased the expression of SIRT1, PGC-1α, Nrf1, and TFAM, and activated mitosis mediated by PINK1/Parkin. This indicates that JYTLD is an important CBD formula targeting mitochondrial dynamics for the treatment of HN.

Hu-lu-ba-wan is a CBD formula for treating kidney yang deficiency, which is made up of *Trigonella L.* and *Psoraleae Fructus*. The Modified Hu-lu-ba-wan (MHLBW) is an improved version of the “Jiaotai Wan” and “Hu-lu-ba-wan,” with main metabolites including *Trigonella L., Achyranthis Bidentatae Radix, Coptis chinensis Franch.,* and *C. cassia (L.) D. Don*. The MHLBW demonstrates significant clinical efficacy for patients with DKD, which is attributed to its antioxidant stress-regulating effects. Gong et al. (Gong et al., 2024) used db/db mice as a DKD model to evaluate the therapeutic effects of MHLBW on mice. They found that MHLBW significantly improved glucose metabolism, as well as reducing basement membrane thickening, mesangial expansion, glomerular fibrosis, and podocyte damage. MHLBW also promotes mitochondrial homeostasis and reverses podocyte apoptosis, which is associated with the regulation of the PKM2/PGC-1α/OPAL pathway. These results may provide a potential strategy for combating DKD.

Tongluo Yishen Decoction (TLYSD) is primarily composed of A*stragali Radix, Rehmanniae Radix Praeparata,* and *Cornus officinalis Sieb. et Zucc.* Its biological activities include inhibiting EMT and anti-fibrotic effects (Cai et al., 2018; Wang et al., 2010; Wang, S. et al., 2020). According to CBD theory, TLYSD possesses therapeutic effects of strengthening the spleen and kidneys, and promoting blood circulation to remove blood stasis. As a result, this formula has been clinically applied in the treatment of CKD for several decades. To investigate its specific mechanisms, Jia Q. et al. (2021) used rats with UUO as an animal model and administered TLYSD orally for 14 days. They then measured renal function indicators such as Scr, BUN, and renal pathological changes. The results showed that the UUO rats exhibited significant mitochondrial dysfunction, including reduced MMP and mitochondrial dynamics imbalance, excessive OS, and PINK1/Parkin-mediated mitochondrial phagocytosis activation. TLYSD treatment markedly elevated MMP, restored balanced mitochondrial dynamics, and mitigated renal injury. It also attenuated mitophagy impairments. In short, the study shows that TLYSD can enhance mitochondrial dynamics by lowering OS and modulating mitophagy, which in turn eases kidney injury, preserves renal function, and curtails renal fibrosis.

Gegen Qinlian Decoction (GGQLD) originates from the Treatise on Exogenous Febrile Disease, composed of four herbal metabolites: *Puerariae Lobatae Radix, Scutellariae Radix, C. chinensis Franch.,* and *Glycyrrhizae Radix et Rhizoma*. The Supplemented Gegen Qinlian Decoction Formula (SGQDF) is derived from the original formula, with the functions of dispersing exterior pathogens, clearing heat, eliminating dampness, and stopping diarrhea. It is primarily used for patients with T2DM and its microvascular complications (Lu J.-Z. et al., 2021; Tian and Huang, 2017). Current research (Wang et al., 2021) indicates that SGQDF can protect podocyte pyroptosis and insulin resistance in an improved DKD rat model. However, it remains unclear whether SGQDF can alleviate podocyte mitochondrial dysfunction and RF in DKD. Therefore, they investigated the therapeutic effects of SGQDF on podocyte mitochondrial dysfunction and RF in DKD, as well as its necrosis-related mechanisms, in subsequent studies. Their results showed (Wang et al., 2024) that SGQDF enhanced renal injury markers in a dose-dependent manner, including serum creatinine, urinary albumin, blood glucose, and blood urea nitrogen. In summary, SGQDF’s beneficial effects *in vivo* and *in vitro* are closely linked to improved mitochondrial function and the inhibition of TNF-α–induced podocyte necrosis.

#### 4.2.4 CBD formulations improve mitophagy to alleviate CKD

Huangqi Danshen Decoction (HQDSD) is a commonly used CBD formula composed of *Astragali Radix* and *Salviae Miltiorrhizae Radix et Rhizoma*, which are frequently prescribed for the clinical treatment of DKD. Research has shown that HQDSD significantly reduces urinary albumin excretion in mice and improves kidney damage, while protecting kidney tissue by activating mitophagy mediated by the PINK1/Parkin pathway. Liu et al. (2019) found that HQDSD significantly reduced urinary albumin excretion and improved kidney damage in DKD mice, while PINK1/Parkin-mediated mitophagy was activated, with increased protein expression and distinct autophagosomes enveloping mitochondria. Additionally, mitochondrial fission increased in the kidneys of DKD mice, suggesting that HQDSD can prevent kidney damage caused by T2DM by inhibiting Pink1/Parkin-mediated mitophagy, thereby exerting a protective effect on the kidneys.

Sanhuang Yishen Capsules (SHYS) are a formula used for tonifying qi and promoting blood circulation. In a study, SHYS was administered to rats with DKD for 8 weeks, and high doses of SHYS significantly reduced the expression levels of VDAC1, Tom20, and COX IV in the kidney tissue of DKD rats. VDAC1 and Tom20 are proteins in the OMM, while COX IV is an essential enzyme in the mitochondrial oxidative phosphorylation reaction (Han et al., 2021). All three are typically strongly associated with mitophagy: when this process is inhibited, the expression of VDAC1, Tom20, and COX IV increases; conversely, when mitophagy increases, their expression gradually decreases. SHYS has also been found to increase the expression of PINK1 and Parkin in the kidney tissue of DKD rats. All these results imply that SHYS promotes PINK1/Parkin-mediated mitophagy in kidney tissue and inhibits NLRP3 inflammasome activation, thereby alleviating mitochondrial damage and inflammatory responses (Li H. et al., 2022).

#### 4.2.5 CBD formulations improve mitochondrial apoptosis to alleviate CKD

Jinchan Yisheng Tongluo Formula (JCYSTLF) is a commonly used formula for treating DN, with the efficacy of nourishing the kidneys and unblocking meridians. JCYSTLF has been clinically applied for the treatment of DKD for decades. However, its specific mechanisms remain unclear. Zhang X. et al. (2024) found that the herbal metabolites in the formula, such as Astragali Radix and Hirudo, can regulate HIF-1α, autophagy, and mitochondrial function, suggesting that JCYSTLF’s treatment of DN may be related to its effects on cellular mitochondrial function. Based on this, they administered JCYSTLF treatment to a DN rat model and found that JCYSTLF treatment significantly reduced proteinuria, serum creatinine, blood urea nitrogen, and uric acid levels in DN rats, while increasing creatinine clearance levels. *In vitro*, the drug serum containing the JCYSTLF formulation increased MMP, improved the activity of mitochondrial respiratory chain complexes I, III, and IV, reduced the percentage of apoptotic cells and the expression of the apoptotic protein Bax, and increased the expression of the anti-apoptotic protein Bcl-2 in HG/hypoxia-induced HK-2 cells. Their research suggests that the JCYSTLF protects renal tubules from mitochondrial dysfunction and apoptosis under diabetic conditions by stabilizing mitophagy, offering a promising therapeutic approach for DN.

Huaiqihuang Granules (HQH) are a CBD formulation composed of *Trametes robiniophia Murr*.*, Lycii Fructus,* and *Polygonati Rhizoma*. In recent years, HQH has shown significant effectiveness in managing CKD. Through analysis of existing clinical data, it has been found that HQH intervenes in the progression of CKD through multiple mechanisms, such as protecting glomerular podocytes, inhibiting the proliferation of glomerular mesangial cells, and preventing renal interstitial fibrosis (Zhang X. et al., 2020). Nuclear factor inhibitors regulate transcription of the NF-κB pathway, thereby regulating apoptosis (An et al., 2019). Guo et al. (2018) found that in cisplatin-induced nephropathy, HQH can reduce the TLR4/NF-κB signaling pathway and protect the kidneys. When HQH was added to cisplatin (CP)-induced nephropathy, it was found that HQH downregulated the PI3K/Akt/mTOR/NF-κB signaling pathway in cisplatin-induced nephrotoxic cells, reducing the expression of p-NF-κB (Zhou Z. et al., 2024). These findings further indicate that HQH suppresses apoptosis and enhances renal cell survival through the NF-κB signaling pathway (Fang et al., 2019). The delicate balance between Bcl-2 and Bax is pivotal in the intrinsic apoptosis process. Upon the activation of apoptosis signals, the pro-apoptotic protein Bax is expressed in the cytoplasm, prompting the discharge of Cyt C from the mitochondria into the cytoplasm. This event creates an apoptosis complex, kick-starting the caspase cascade reaction and culminating in cell apoptosis. Fang et al. (2019) found that, compared with the control group, the ratio of pro-apoptotic proteins to anti-apoptotic proteins was relatively balanced in the Huaier polysaccharide (HP)-treated group, resulting in reduced cell apoptosis. Additionally, GRP 78 can bind to and inhibit the activity of the pro-apoptotic protein BIK on the endoplasmic reticulum, thereby promoting the expression of Bcl-2 and inhibiting cell apoptosis. Li T. X. et al. (2017) treated hyperglycemic MPC-5 podocytes with HQH and observed that HQH markedly counteracted the elevated GRP78 levels and its regulation of the Bcl-2/Bax signaling pathway, while also suppressing caspase-3 expression, thereby mitigating podocyte injury. These findings suggest HQH mitigates CKD progression by targeting mitochondrial apoptosis.

### 4.3 CBD formulations improve the gut-kidney axis to alleviate CKD

The renal protective effects of CBD hinge on its core mechanism of targeting the gut microbiota-microbial metabolites-mitochondrial axis. Research indicates that CBD formulations can reshape gut microbial community structure by enriching the relative abundance of SCFA-producing functional bacteria, such as those belonging to the *Bacteroidetes phylum*, thereby significantly elevating levels of SCFAs, including acetate, propionate, and butyrate (Shi et al., 2023). In the pathological state of CKD, the functional restoration of this microbial metabolic axis represents a key pathway through which CBD exerts its nephroprotective effects. This mechanism partially relies on the selective enrichment of SCFA-associated microbial taxa (Zhou X. et al., 2024). By promoting SCFA production and enhancing mitochondrial functional integrity, CBD significantly reduces systemic inflammatory burden and improves glomerular filtration function, ultimately achieving comprehensive protection and regulation of renal function.

Hibiscus Capsules (HC) primarily contain hibiscus flower extract, rich in various flavonoid metabolites. These bioactive metabolites undergo biotransformation *in vivo* into glucuronic acid-sulfate conjugates, exhibiting multifaceted pharmacological properties including metabolic enhancement, antihypertensive effects, improved antioxidant stress, and antioxidant activity in DKD, HN, and glomerulonephritis (Cao et al., 2022; Chen et al., 2016). HC demonstrates significant anti-proteinuria efficacy and has been approved by China’s National Medical Products Administration for treating chronic nephritis (Li et al., 2021). In adenine-induced chronic renal failure models, Hibiscus Capsules effectively prevent tubulointerstitial fibrosis by inhibiting the NADPH/ROS/ERK signaling pathway (Cai et al., 2017). Furthermore, in a 5/6 Nx model, they reduced uremic toxin accumulation by alleviating renal burden through tryptophan transport disruption and microbial metabolism regulation (Lu P.-H. et al., 2021).

Jiangtang decoction (JTD), a patented Chinese herbal remedy (Patent Number: 20141002188.3), has gained widespread recognition in clinical practice for its utility in managing DKD (Hong et al., 2017). The formulation comprises *Euphorbia humifusa Willd*, *Salvia miltiorrhiza Bunge*, *Astragali Radix*, *Asphodelus asphodeloides Bunge*, and *Cistanche chinensis Franch*. Research by Hong et al. (Hong et al., 2023) indicates that JTD possessed the ability to modulate the abundance of *Rikenella, Lachnoclostridium, unclassified_c_Bacilli,* and *norank_f_Lachnospiraceae* within the gut microbiota. This modulation subsequently exerted an impact on metabolic processes, kidney function, uremic toxin accumulation, and inflammation. As a result, these multifaceted effects collectively contributed to the amelioration of DKD.

Yishen Qingli Heluo Granules (YSQLHLG) is the flagship CBD formula for treating CKD. Research (Sun X. et al., 2022) indicates that YSQLHLG treatment significantly increased the relative abundance of SCFA-producing *Lactobacillaceae*, *Lactobacillus*, and *Lactobacillus gasseri* in experimental CKD models, while elevating total intestinal SCFA concentrations. Microbiome transplantation studies further elucidated that YSQLHLG’s renal protective mechanism is partially mediated through gut microbiota regulation, particularly via bacterial populations involved in SCFA biosynthesis.

Yishen Huashi Granules (YSHSG) exhibit significant renal protective effects. Research indicates (Dong et al., 2024) that YSHSG treatment significantly reduces 24-h proteinuria in non-dialysis CKD patients while promoting the growth of beneficial gut microbiota (e.g., *Faecalibacterium, Spirillaceae, Fusobacterium, Sutterella*) and suppressing potentially pathogenic bacterial species. Subsequent analysis revealed that increased levels of *Clostridium* and *Fusobacterium* were negatively correlated with reduced 24-h proteinuria. Steroid hormone biosynthesis and biotin metabolism. Notably, microbial alterations associated with steroid hormone synthesis regulation exhibited gender-specific differences: male patients showed increased *Clostridium* and *Fusobacterium*, while female patients demonstrated a significant rise in *Prevotella*.

Other commonly used CBD, including Moshen Granules (MSG), Qiwei Granules (QWG), Tongxinluo Granules (TXLG), Liuwei Dihuang Wan (LWDHW), and Chaihuang Yishen Granules (CHYSG), have demonstrated the ability to mitigate renal fibrosis and tissue structural damage under the pathologic conditions of CKD primary pathogenesis by regulating multiple cytokines (Li J. et al., 2024; Wang S. et al., 2023).

Research on CBD formulations faces even greater challenges than studies on isolated metabolites. A fundamental issue is the lack of standardization; the chemical composition of formulation can vary significantly between batches and manufacturers, threatening reproducibility. The observed effects are attributed to the whole formulation, but the specific active constituents responsible for MQC modulation are almost never identified. Claims of “multi-target” actions are common, but without a clear understanding of the pharmacokinetics and tissue distribution of the key components, it is difficult to distinguish primary targets from secondary effects. The majority of formulation studies are phenomenological, showing improved outcomes and associated changes in MQC-related proteins, but they fall short of providing deep mechanistic insights. There is a pressing need to deconstruct these formulations, identify key active combinations, and employ network pharmacology and omics technologies to build testable hypotheses about their mechanisms of action.

**FIGURE 5:**
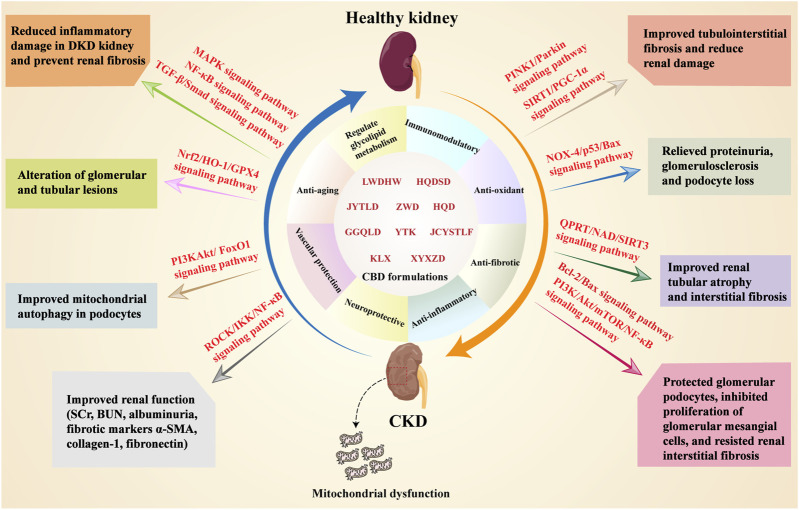
Schematic diagram of CBD formulation improving CKD by regulating mitochondrial function Renal mitochondrial dysfunction is one of the important causes of CKD. CBD formulation relieves various clinical manifestations of CKD through the MQC pathway, including proteinuria, decreased GFR, and renal fibrosis, involving NF-κB, PINK/Parkin, and Nrf2/HO-1 signaling pathways. CBD formulation alleviates the development of CKD by alleviating inflammatory damage to the kidney of DKD, alleviating oxidative stress in podocytes, reducing renal extracellular matrix deposition, and increasing mitophagy levels. Abbreviations: LWDHW: Liuwei Dihuang Wan; HQDSD: Huangqi Danshen Decoction; JYTLD: Jiangya Tongluo Decoction; ZWD: Zhenwu Decoction; HQD: Huangqi Decoction; GGQLD: Gegen Qinlian Decoction; YTK: Yitangkang; JCYSTLF: Jinchan Yishen Tongluo Formula; KLX: Keluoxin; XYZD: Xiaoyu Xiezhuo Decoction.

Details of the relevant experiments and the mechanism of action of the CBD formulations are given in Table 1; Figure 5 below.

**TABLE 1:** Mechanism of MQC-targeted CBD formulations therapy for CKD.

Targets	CBD formulations	Formulation composition	Cellular models	Animal models	Dose	Duration time	Negative/positive control	Outcomes	Mechanisms	References
Mitochondrial oxidative stress	Nourishing yin and promoting blood flow recipe (NYPBR)	*Rehmannia glutinosa (Gaetn.) Libosch. ex Fisch. et Mey., Yam, Corni Fructus, and Moutan Cortex*	-	STZ (rats)	3 g/day (oral gavage)	13 weeks	NC: -; PC: -	Improved mitochondrial OS to delay and alleviated the progression of DN	Not yet explored	Li et al. (2009)
	Liuwei Dihuang Wan (LWDHW)	*Rehmannia glutinosa (Gaetn.) Libosch. ex Fisch. et Mey., Paeonia suffruticosa Andr.,*and *yams, etc.*	-	STZ (rats)	6.75 g/kg/day (oral gavage)	12 weeks	NC: -; PC: -	Alleviated inflammatory damage to the kidneys, prevented renal fibrosis, and protected glomerular mesangial cells	SMADS, MAPK, and NF-κB signaling pathways	Pengyu and Yue (2019)
	Shenqi Dihuang Decoction (SQDHD)	*Panax notoginseng (Burkill) F.H.Chen, Astragali Radix, Rehmanniae Radix Praeparata, and Poria cocos (Schw.) Wolf*	HK-2 (HG-induced)	-	Low-dose group (2.5% drug-containing serum), medium-dose group (5% drug-containing serum), and high-dose group (10% drug-containing serum)	-	NC: -; PC: -	Improved glomerular and tubular lesions	Nrf2/HO-1/GPX4 signaling pathway	Wang Z.-B. et al. (2023)
	Zhen Wu Decoction (ZWD)	*Panax notoginseng (Burkill) F.H.Chen, Aconiti Lateralis Radix Praeparata, Poria cocos (Schw.) Wolf, and Paeoniae Radix Alba*	-	db/db (mice); UUO (mice)	33. 8, 16. 9, and 8. 45 mg/kg/day (oral gavage)	8 weeks	NC: -; PC: Irbesartan (25 mg/kg/day) (oral gavage)	Enhanced kidney function (SCr, BUN, albuminuria, fibrotic markers α-SMA, collagen-1, fibronectin); Enhanced mitochondrial DNA quantity, increased ATP synthesis, decreased mtDNA leakage; Suppressed TGF-β1 production; Reduced Nrf2 and TFAM expression, inhibiting STING signaling pathway and improving mitochondrial OXPHOS	ROCK/IKK/NF-κB signaling pathway; Nrf2/HO-1/GPX4 signaling pathway; Nrf2/STING/TFAM signaling pathway	Jin et al. (2023), Zhao Z. et al. (2024)
Mitochondrial biosynthesis	Danggui Buxue Decoction (DGBXD)	*Astragali Radix, Angelica sinensis (Oliv.) Diels, Cinnamon, and Rehmanniae Radix Praeparata*	-	STZ (rats)	4.7, and 9.4 mg/kg/day (oral gavage)	4 weeks	NC: -; PC: Glimepiride (0.4 mg/kg/day) (oral gavage)	Inhibited mitochondrial division and apoptosis in podocytes, alleviated oxidative stress in podocytes, and reduced inflammatory responses	MFN2 and PCG-1α	Sun, L. et al. (2022), Zhang Y. et al. (2023)
	YiTangKang (YTK)	*Astragali Radix, Polygonati Rhizoma, and Salvia miltiorrhiza Bunge, etc.*	-	STZ (rats)	10, 20, and 40 mg/kg/day (oral gavage)	8 weeks	NC: -; PC: Irbesartan (25 mg/kg/day) (oral gavage)	Improved mitophagy in podocytes of DKD rats and alleviated kidney damage	PI3KAkt/FoxO1 signaling pathway; JAK2/STAT3 signaling pathway	CHENG et al. (2024)
	Huangqi decoction (HQD)	*Astragali Radix, Poria cocos (Schw.) Wolf, Plantago asiatica L., Ophiopogon japonicus (L. f.) Ker Gawl., and Rehmanniae Radix Praeparata*	Podocytes	STZ (mice)	Cellular Model: 0, 10, 30, 100, 300, 1000, 3000 10,000 and 30,000 μg/mL; Animal Model: 1.08 g/kg/day	Cellular Model: 24 h; Animal Model: 8 weeks	NC: -; PC: Irbesartan (13.5 mg/kg/day)	Reduced podocyte apoptosis; alleviated progressive proteinuria, glomerulosclerosis, and cell loss in DN mice	Nox4/p53/Bax and AMPK signaling pathway	Li et al. (2019)
Mitochondrial dynamics	Shenshuai II Recipe (SSR)	*Codonopsis Radix, Epimedii Folium, Salvia miltiorrhiza Bunge,* etc.	NRK-52 E (HG-induced)	5/6Nx (rats)	Cellular Model: 1%, 2% and 5% SSR-medicated serum; Animal Model: -	Cellular Model: -; Animal Model: -	NC: -; PC: Fenofibrate	Anti-renal fibrosis	PGC-1α	Wang L. et al. (2023), Wang et al. (2022b)
	Huangqi Dansheng Decoction (HQDSD)	*Astragali Radix, Angelicae Sinensis Radix, Salvia miltiorrhiza Bunge, and other metabolites*	-	Adenine-induced (mice)	6.8 g/kg/day (oral gavage)	12 weeks	NC: -; PC: -	Improved tubular atrophy and interstitial fibrosis in CKD rats	Drp1 and Mid 49/51; OPA1	Liu et al. (2020a), Lu et al. (2024)
	Jian-Pi-Yi-Shen formula (JPYSF)	*Astragali Radix, Yam, and Atractylodes macrocephala Koidz., etc.*	-	5/6Nx (rats)	10.89 g/kg/day (oral gavage)	12 weeks	NC: -; PC: -	Attenuation of renal tubular atrophy and interstitial fibrosis with decreased extracellular matrix deposition in the kidneys	QPRT/NAD/SIRT3 signaling pathway	Gao et al. (2023), Li C. et al. (2023), Li et al. (2025), Liu et al. (2021)
​	Jiangya Tongluo Decotion (JYTLD)	*Margaritifera Concha, Chrysanthemum L., Scutellariae Radix, and Salvia miltiorrhiza Bunge, etc.*	-	Spontaneous hypertensive rat (SHR)	14.2 g/kg/day (oral gavage)	12 weeks	NC: -; PC: Valsartan (30 mg/kg/d) (oral gavage)	Improved renal tubular interstitial fibrosis	PINK1/Parkin and SIRT1/PGC-1α signaling pathway	Zhao Y. et al. (2024)
	Modified Hu-lu-ba-wan (MHLBW)	*Trigonella L., Achyranthis Bidentatae Radix, Coptis chinensis Franch., and Cinnamomum cassia (L.) D. Don, etc.*	-	db/db (mice)	8.9 and 17.8 g/kg/day (oral gavage)	7 weeks	NC: -; PC: -	Enhanced glucose metabolism, thickening of the basement membrane, mesangial expansion, glomerular fibrosis, and podocyte damage	PKM2/PGC-1α/OPA1 signaling pathway	Gong et al. (2024)
	Tongluo yishen decoction (TLYSD)	*Astragali Radix, Rehmanniae Radix Praeparata, and Cornus officinalis Sieb. et Zucc., etc.*	-	UUO (Rats)	7.8 g/kg/day (oral gavage)	14 days	NC: -; PC: Valsartan (30 mg/kg/d) (oral gavage)	Reduced kidney damage, protects kidney function, and reduces kidney fibrosis	Pink1/Parkin signaling pathway	Jia Q. et al. (2021)
	Gegen Qinlian Decoction (GGQLD)	*Puerariae Lobatae Radix, Scutellariae Radix, Coptis chinensis Franch., and Glycyrrhizae Radix et Rhizoma*., etc.	Podocytes	STZ/HFD (Rats)	Cellular Model: 7.5% SGQDF -medicated serum; Animal Model: 17.7 and 8.85 g/kg/day (oral gavage)	Cellular Model: 24 h; Animal Model: 4 weeks	NC: -; PC: EMPA (1.042 mg/kg/d) (oral gavage)	Amelioration in renal injury markers, including body weight, blood glucose, serum creatinine, blood urea nitrogen, and urinary albumin	RIPK1/RIPK3/MLKL axis	Wang et al. (2024)
Mitophagy	San-Huang-Yi-Shen capsule (SHYSC)	*Astragali Radix, Rehmanniae Radix, and Cornus officinalis Sieb. et Zucc., etc.*	-	STZ (rats)	0.81 g/kg and 1.62 g/kg/day (oral gavage)	8 weeks	NC: -; PC: -	Reduced proteinuria and protected kidney function	PINK1/Parkin signaling pathway	Li et al. (2022a)
	Huangqi-Danshen decoction (HQDSD)	*Astragali Radix and Salvia miltiorrhiza Bunge, etc.*	-	db/db (mice)	4.7 g/kg/day (oral gavage)	4 weeks	NC: -; PC: -	Reduced urinary albumin in mice and improved kidney damage	PINK1/Parkin signaling pathway	Liu et al. (2020b)
​	QiDiTangShen granules (QDTSG)	*Astragali Radix, Sophora flavescens Aiton, Rehmanniae Radix, Sophora japonica L., Euryales Semen, Rheum rhaponticum L., and Herba Hedyotidis Diffusae, etc.*	-	db/db (mice)	-	12 weeks	NC: -; PC: Valsartan	Anti-renal fibrosis	AMPK/mTOR signaling pathway	Wang X. et al. (2019)
	Keluoxin (KLX)	*Astragali Radix, Ligustri Lucidi Fructus, Rheum rhaponticum L., and Lycii Fructus, etc*.	CBDK-1	Radiation nephropathy (mice)	Cellular Model: Keluoxin -medicated serum; Animal Model: 900 mg/kg/day (oral gavage)	Cellular Model: 48 h; Animal Model: 4 months	NC: -; PC: -	Reduced kidney damage and inflammation	JAK/STAT signaling pathway	Deng et al. (2023)
	Yiqi Jiedu Huayu Decoction (YQHYD)	*Astragali Radix, Angelicae Sinensis Radix, Salvia miltiorrhiza Bunge, and Chuanxiong Rhizoma, etc*.	-	STZ/HFD (rats)	High-dose, medium-dose and medium-dose (oral gavage)	12 weeks	NC: -; PC: Irbesartan (oral gavage)	Improved podocyte damage and reduced renal fibrosis	AMPK and PI3K/Akt signaling pathway	Xuan et al. (2021)
Mitochondrial apoptosis	JinChan YiShen TongLuo Formula (JCYSTLF)	*Cordyceps sobolifera (Hill) Berk. et Br., Scrophularia ningpoensis Hemsl., Astragali Radix, Curcuma zedoaria (Christm.) Rosc., and Cyathula officinalis Kuan, etc*.	HK-2 (HG-induced)	Unilateral nephrectomy (rats)	Cellular Model: JCYSTL formula containing serum (10%); Animal Model: 15 g/kg/day (oral gavage)	Cellular Model: -; Animal Model: 12 weeks	NC: -; PC: -	Preserved renal tubules by preventing mitochondrial damage and cell death in diabetic conditions	HIF-1α/PINK1/Parkin signaling pathway	Zhang X. et al. (2024)
	Huaiqihuang Granule (HQH)	*Trametes robiniophia Murr*., *Lycii Fructus, and Polygonati Rhizoma, etc.*	Podocytes	AKI (mice)	Cellular Model: 0/6/12/18 mg/mL; Animal Model: 6 g/kg/day (oral gavage)	Cellular Model: 12 h; Animal Model: 33 days	NC: -; PC: -	Reduced podocyte apoptosis	PI3K/Akt/mTOR/NF-κB signaling pathway; Bcl-2/Bax signaling pathway	Fang et al. (2019), Guo et al. (2018), Li, T. X. et al. (2017)
	Qufeng Tongluo Decoction (QFTLD)	*Dioscorea nipponica Makino, Fructus Arctii, and Bombyx Batryticatus, etc.*	MPC-5 (HG-induced)	-	10 μg/mL	48 h	NC: -; PC: -	Inhibited autophagic flux in podocytes	PI3K/Akt signaling pathway	Ruan et al. (2024)
Gut-kidney axis	Jiangtang decoction (JTD)	*Euphorbia humifusa Willd*, *Salvia miltiorrhiza Bunge*, *Astragali Radix*, *Asphodelus asphodeloides Bunge*, and *Cistanche chinensis Franch*	-	KK-Ay mice	4 g/kg/day (oral gavage)	4, 8, and 12 weeks	NC: -; PC: Irbesartan (30 mg/kg/day) (oral gavage)	Improved metabolism, kidney function, uremic toxins, and inflammatory responses while regulating the gut microbiota	Kidney injury molecule-1 (KIM-1), TMAO, pCS, NLRP3 and IL-17A	Hong et al. (2023)
	Yishen Qingli Heluo Granules (YSQLHLG)	*Astragali Radix, Cornus officinalis Siebold and Zucc. And Eucommia ulmoides, etc.*	-	5/6 Nx (rats)	5.6 g/kg/day (oral gavage)	8 weeks	NC: -; PC: -	Reduced renal fibrosis and inflammation, n, reestablished bacterial communities, and improved the intestinal barrier	SCFA-producing bacteria (i.e., *Lactobacillaceae, Lactobacillus,* and *Lactobacillus_gasseri*)	Sun X. et al. (2022)
	Yishen Huashi Granules (YSHSG)	*Astragali Radix, Panax ginseng C. A. Mey., Atractylodes macrocephala Koidz., and Poria cocos (Schw.) Wolf cocos (Schw.) Wolf, etc.*	-	STZ (rats)	2.27 and 5.54 g/kg/day	6 weeks	NC: -; PC: Valsartan (7.38 mg/kg/day)	Improved glycerolphospholipid metabolism in DKD rats	*Lactobacillus* and *Lactobacillus*_murinus	Han, C. et al. (2023)

## 5 Summary and outlook

As modern society has developed, the prevalence of CKD has been on the rise globally, not only significantly increasing the economic burden on society and families but also becoming a very serious public health issue. The pathogenesis of CKD is complex, involving mitochondrial dysfunction (Fontecha-Barriuso et al., 2020), inflammatory responses (Saurav et al., 2023), and metabolic disorders (Mitrofanova et al., 2023), ultimately leading to a decrease in GFR. In recent years, a significant rise has been observed in studies targeting mitochondrial impairments as a possible therapeutic avenue for multiple diseases. The primary domains of mitochondrial dysfunction include mitochondrial oxidative stress, mitochondrial dynamics, mitochondrial biogenesis, mitophagy, and mitochondrial apoptosis. These processes of mitochondrial dysfunction are closely associated with the dysregulation of the MQC, and collectively contribute to the progression of kidney disease. The pervasive consequences of OS on mitochondrial dynamics, mitochondrial biogenesis, and mitochondrial mitophagy processes elucidate this relationship. Excessive OS, in synergy with mitochondrial dynamics disruption, results in mitochondrial fragmentation and the subsequent activation of inflammatory pathways. In the event of impaired mitochondrial dynamics, the capacity of mitochondria to adapt to fluctuating cellular energy demands is compromised, thereby giving rise to inadequate energy production. The process of mitochondrial fragmentation and the subsequent cell death processes require adequate mitophagy to eliminate damaged mitochondria and maintain mitochondrial homeostasis.

Despite the growing recognition of the importance of MQC in kidney disease, there is a paucity of influential studies that specifically target MQC, particularly in the context of CKD. Understanding MQC’s role in CKD is crucial for accurate diagnosis and targeted therapy. In consideration of the aforementioned points, this review outlines the pathways through which MQC drives the development of CKD (Figure 2). Its mechanisms of action can be summarized as follows: participation in the regulation of upstream and downstream factors, such as PGC-1α, AMPK, and SIRT, is conducive to the enhancement of mitochondrial biogenesis. Participation in the regulation of dynamic proteins such as MFN1, MFN2, and Drp1 is conducive to the promotion of mitochondrial fusion, the inhibition of mitochondrial fission, and the maintenance of stable mitochondrial morphology. Participation in the regulation of ROS production, the inhibition of mitochondrial oxidative stress, the increase of ATP production, and the restoration of mitochondrial energy metabolism function are also beneficial. The activation of the PINK1/Parkin signaling pathway is achieved through the upregulation of PINK1, Parkin, and LC3-II expression. This process promotes mitophagy while simultaneously inhibiting excessive mitophagy. The purpose of this is to maintain a steady-state balance of mitochondrial self-clearance. It has been demonstrated to participate in the regulation of the expression of pro-apoptotic proteins, including Bax, Bak, Bim, Puma, and Noxa, as well as anti-apoptotic proteins, such as Bcl-2, Bcl-xl, Bcl-w, Mcl-1, and Bcl-G. The result of this process is a reduction in MMP and increased mitochondrial membrane permeability. Currently, few primary medications targeting these pathways are accessible for CKD treatment. These include sodium-glucose cotransporter 2 (SGLT2) inhibitors, antioxidants, and CD38 inhibitors (Ogura et al., 2020; Upadhyay, 2024; Xiao et al., 2017). SGLT2 is predominantly found in the proximal tubules. During clinical trials with individuals suffering from CKD-related damage, SGLT2 inhibitors have been shown to not only soothe the issues with mitochondria but also to stimulate the process of mitochondrial phagocytosis. Therefore, the effects of SGLT2 inhibitors are encouraging. However, SGLT2 inhibitors still have certain adverse reactions in clinical application, the most common of which is ketoacidosis, which can pose a serious threat to patient safety in severe cases (Chow et al., 2023). Mitoquinone mesylate (MitoQ) is a targeted antioxidant that not only inhibits oxidative stress and improves abnormal mitochondrial dynamics in CKD but also plays a key role in mitophagy (Xiao et al., 2017). However, studies have shown (Gottwald et al., 2018) that MitoQ may induce rapid swelling and depolarization of mitochondria in renal proximal tubule cells, which, to some extent, limits its use in CKD. Alongside the previously listed medications, there are specific MQC-targeted therapies for CKD, with their mechanisms of action and usage limits detailed in Table 2.

**TABLE 2:** Current metabolites targeting MQC for CKD treatment and their limitations.

Targets	Compounds	Mechanisms	Outcomes	Limitations	References
Antioxidant	MitoQ	mtROS	Improved macrovascular function and microvascular function	Induced mitochondrial swelling in proximal tubule cells of the kidney	Gottwald et al. (2018), Kirkman et al. (2023)
	L-carnitine	SOD2, TLR9/TNF-α	Reduced mtROS production and circulating mtDNA content, reduced albuminuria	Increased risk of atherosclerosis	Ito et al. (2022), Koeth et al. (2013)
	N-acetylcysteine	Drp1/Fis1, Opa1/Mfn1, SIRT3/SOD2/GPx4	Reduced ROS	Allergic reaction	Cepaityte et al. (2023), Li et al. (2022c)
	Ulinastatin (urinary trypsin inhibitor)	Gut-kidney axis Drp1, Fis1	Improved SCr, urine creatinine, urine volume/24 h, CrCl, BUN, urinary albumin, glomerular morphology, renal NF-κB; Reduced mtROS, OS markers (renal H_2_O_2_, 8-OHdG levels)	Anaphylactic shock, significantly reduced white blood cell count, and poor compliance	Rizk et al. (2023)
Biogenesis activators	Melatonin	AMPK/SIRT1/PGC-1α, Nrf2, TFAM	Improved renal function (urine creatinine and urea, kidney weight/body weight ratio, albuminuria) and renal injury; Enhanced expression of AMPK, SIRT1/3, PGC-1α, and TFAM; Reduced renal OS, increased renal antioxidant levels (GSH, GPx) Improved mitochondrial dysfunction (enhanced complex I, II, and ATP synthase activity, prevented loss of MMP)	Long-term use of melatonin may be associated with an increased risk of fractures	Frisher et al. (2016), Siddhi et al. (2022)
	Exendin-4 (GLP-1 receptor agonist)	AMPK-fatty acid	Enhanced AMPK signaling to regulate mitochondrial respiration and glycolysis; Restored ATP production and baseline oxygen consumption rate	Inducing acute pancreatitis	Ayoub et al. (2010), Shen et al. (2024)
	Nicotinamide riboside	SIRT3/cGAS-STING, PGC-1α, Nrf1, TFAM1, electron transport chain complexes I, IV	Improved renal function (albuminuria, urinary kidney injury marker-1 excretion, pathological changes, profibrotic markers, e.g., α-SMA)	Long-term use increases the burden on the gastrointestinal tract and damages the liver and kidneys	Myakala et al. (2023)
	Sodium butyrate	AMPK/PGC-1α, Nrf1, Mfn2 and p-Drp1	Improved renal function (BUN, urine creatinine, pathological changes); Reduced apoptosis (decreased cleaved-caspase3 and Bax, increased Bcl-2 expression); Increased ATP content, decreased ROS, increased PGC-1α and AMPK expression, increased expression of mtTFA, Nrf1, Mfn2, decreased p-Drp1	Long-term, high intake of sodium butyrate may alter the composition of microorganisms in the intestine, adversely affecting intestinal health	Yu et al. (2023)
Fission inhibitors/Fusion activators	Finerenone (nonsteroidal mineralocorticoid receptor antagonist)	Mineralocorticoid receptor/PI3K/Akt/eNOS, Drp1, Fis1, Mfn2, OPA, LC3-II, Atg5, Beclin-1	Improved renal function (urine ACR, SCr) and morphological changes; Reduced mitochondrial fragmentation and fission (Drp1, Fis1), recovered Mfn2, OPA, LC3-II, Atg5, Beclin-1 protein levels; Reduced apoptosis (Bax, Cyt C, overall activity); Reduced mtROS	Elevated blood creatinine or decreased eGFR	Yao et al. (2023)
	Alpha lipoamide	RXRα/CDX2, CFTR/β-catenin, Drp1, Mfn1	Improved renal fibrosis; Decreased Drp1, increased Mfn1, decreased ROS, increased ATP content Decreased apoptosis (reduced Bax, increased Bcl-2); Upregulated and activated RXRα	Kidney damage, hypoglycemia (when used in combination with hypoglycemic drugs)	Zhang, H.-F. et al. (2023)
	Formoterol	Drp1, Mfn1	Restored electron transport chain proteins, ATP production, and oxygen consumption; Restored Drp1 and Mfn1 levels	Arrhythmia	Cleveland et al. (2020)
Mitophagy regulators	Paricalcitol	VDR, PINK1, Parkin BNIP3	Improved renal function (SCr, BUN ACR, proteinuria, reduced kidney fibrosis histologically and fibrosis markers α-SMA, COL1, fibronectin); Reversed abnormal mitochondrial morphology, restored mitophagy defects (restored PINK1, Parkin, BNIP3, TOM20, LCE-II, SQSTM1 protein expression)	Hypercalcemia, osteoporosis, etc.	Yang et al. (2024)
	Calcitriol	VDR, Mfn2, Fis1, PINK1 Parkin, Mfn2/MAMs/FUNDC1, Mfn2/SERCA2	Reduced ROS, increased MMP and ATP production, restored MAM integrity	Hypercalcemia and hypercalciuria	Chen H. et al. (2024)

The gut microbiota is a single molecular entity capable of producing crucial molecules or substances that play a pivotal role in various diseases, particularly including CKD. Increasing evidence (Aquilani et al., 2022; Han et al., 2025; Yang et al., 2025) from gut-related research indicates that mitochondrial dysfunction and alterations in mitochondrial structure represent potential intervention strategies for CKD. The development of CKD is closely linked to the gut microbiota, metabolites, and mitochondria, with microbial metabolites serving as a crucial bridge in the progression of CKD. This paper primarily explores the mechanisms of the gut microbiota-microbial metabolites-mitochondrial axis in CKD development, though fundamental questions remain regarding its role. Specifically, which gut bacteria or microbial metabolites can regulate mitochondrial function? Second, whether supplementation with different probiotics, prebiotics, or microbial metabolites can slow CKD progression by mitigating mitochondrial damage. Finally, the molecular targets and mechanisms by which microbial metabolites improve or impair mitochondrial function remain unclear. Despite these limitations, a growing body of recent research (Dong et al., 2024; Hong et al., 2017; Sun X. et al., 2022) has confirmed the pivotal role of the gut microbiota-microbial metabolite-mitochondrial axis in CKD. For instance, JTD modulates the abundance of *Rikenella, Lachnoclostridium, unclassified_c_Bacilli,* and *norank_f_Lachnospiraceae* within the gut microbiota to improve DKD. Besides, YSQLHLG treatment significantly increased the relative abundance of SCFA-producing *Lactobacillaceae, Lactobacillus*, and *Lactobacillus gasseri* in an experimental CKD model, while also elevating total intestinal SCFA concentration. Furthermore, a recent study (Tao et al., 2019) involving 14 biopsy-confirmed cases demonstrated a direct association between gut microbiota and CKD: Compared to healthy controls and patients with diabetes alone, CKD patients exhibited reduced gut microbiota diversity, increased *Proteobacteria*, significantly elevated Escherichia-Shigella, and markedly decreased *Prevotella_9* (*Escherichia-Shigella* and *Prevotella_9* effectively distinguish the presence of diabetic nephropathy). Therefore, modulating gut microbiota via the gut-kidney axis may represent a potential therapeutic strategy for CKD patients, particularly those with gut dysfunction complications.

**FIGURE 6:**
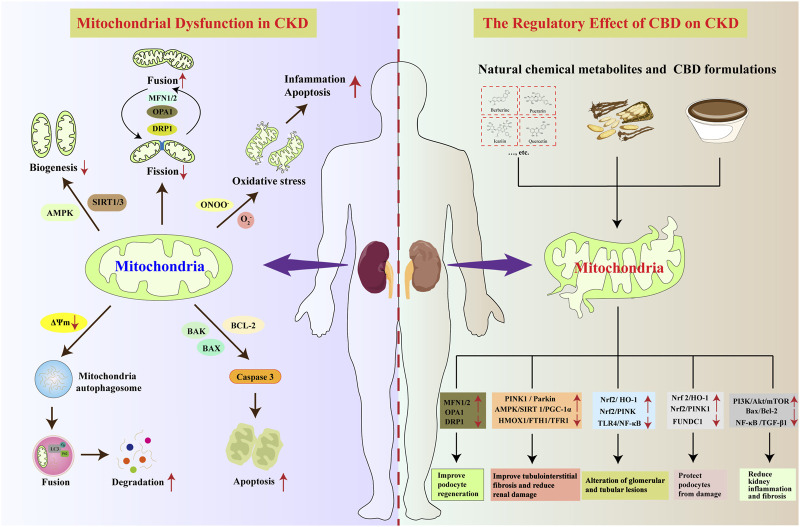
The natural chemical metabolites and formulations improve CKD through the mitochondrial pathway. The natural chemical metabolites and formulation alleviate symptoms of CKD by improving mitochondrial dysfunction through multiple mechanisms. These primarily include promoting biogenesis, enhancing mitochondrial dynamics, exerting antioxidant effects, boosting mitophagy, and counteracting mitochondrial apoptosis. Collectively, these effects promote mitochondrial health, thereby reducing inflammation and tissue damage.

CBD represents a significant cultural asset of the Chinese nation. The advent of modernization in the field of CBD has resulted in a new methodology for the regulation of MQC in the management of CKD. This development has demonstrated considerable promise and potential for further exploration. With regard to safety, CBD has been shown to generally exhibit higher safety and tolerability in comparison with certain drug therapies. For instance, Exendin-4 has been observed to induce acute pancreatitis (Ayoub et al., 2010), thus limiting its application in the treatment of CKD. Conversely, CBD rarely reports adverse reactions, making it appropriate for long-term management of disease. Regarding the issue of treatment stability, CBD is typically concerned with the regulation of overall balance, with a view to modulating the immune system and improving the body’s overall condition. With regard to individualized treatment, CBD emphasizes pattern differentiation and treatment. Classification of CKD generally includes various phenotypes, such as spleen-kidney qi deficiency syndrome, liver-kidney yin deficiency syndrome, and spleen-kidney yang deficiency syndrome. Treatment plans are tailored to the patient’s constitution and medical characteristics with a view to enhancing treatment specificity and efficacy. For instance, in the case of spleen-kidney yang deficiency syndrome, the modified Zhenwu Decoction has been employed as a therapeutic agent. Furthermore, CBD can exert its effects through multiple targets, thereby enhancing therapeutic outcomes. For example, the CBD metabolite AS-IV can act on the mitochondrial OS pathway, promote mitochondrial biogenesis, and reduce mitochondrial apoptosis, thereby improving the pathological symptoms of CKD from multiple aspects (Gui et al., 2023; Shen Q. et al., 2023). Zhenwu Decoction influences mitochondrial biogenesis and regulates mitochondria through oxidative stress to achieve renal protective effects (Zheng et al., 2023). The natural chemical metabolites and formulations targeting the MQC system for treating CKD are summarized in Figure 6. In conclusion, CBD presents multiple benefits in the management of CKD, and targeting mitochondria to explore treatment methods for CKD is a promising direction.

The natural chemical metabolites and formulation alleviate symptoms of CKD by improving mitochondrial dysfunction through multiple mechanisms. These primarily include promoting biogenesis, enhancing mitochondrial dynamics, exerting antioxidant effects, boosting mitophagy, and counteracting mitochondrial apoptosis. Collectively, these effects promote mitochondrial health, thereby reducing inflammation and tissue damage.

Despite the significant potential of CBD in targeting the MQC system to manage CKD, research in this field remains in its infancy. The mechanisms by which CBD metabolites and formulations alleviate mitochondrial damage remain unclear, and many questions remain to be addressed. Firstly, the progression of CKD is a multifaceted and ever-changing pathological process, with various cytokines and signaling pathways likely playing a role in driving the disease forward in addition to mitochondrial dysfunction. Secondly, there is no gold standard for mitochondrial dysfunction, and current assessments primarily rely on references such as mitochondrial morphology under electron microscopy, mitochondrial energy metabolism indicators, mitochondrial dynamics indicators, and mitochondrial biogenesis indicators. Mitochondrial dysfunction is a multifaceted condition involving multiple signaling pathways. The potential for synergistic interactions among these pathways requires further elucidation through rigorous investigation. Additionally, CBD formulations are characterized by their complexity and diversity, and the specific metabolites within them have not been thoroughly analyzed or discussed in existing studies. Consequently, future research should employ contemporary biotechnology to undertake in-depth, multidimensional explorations of the composition, pharmacological effects, and target pathways of specific drugs. These studies should analyze the pharmacological effects and target pathways of individual metabolites, and examine the pharmacological effects and interactions of these metabolites. This will facilitate the development of new pharmaceutical combinations, thereby rendering treatment more precise and facilitating better clinical application. This, in turn, will promote broader acceptance of CBD among CKD patients. Moreover, research on the use of CBD to regulate mitochondrial dysfunction in the treatment of CKD is largely confined to animal or cell experiments, with a paucity of high-level, multi-center clinical trials. Consequently, there is a clear requirement for extensive clinical validation. Although the probability of adverse effects from the use of CBD is quite low, there have been recorded cases of renal toxicity (Lee et al., 2011). Finally, in terms of treatment strategies, guidance is lacking on how to integrate CBD with modern pharmaceuticals, making it essential to develop standardized protocols for managing CKD. The integration of CBD with nanomedicine to target the kidneys with precision and address the issue of low oral bioavailability may represent the optimal approach for treating CKD through the integration of traditional and modern medicine. Addressing the aforementioned issues will facilitate a deeper understanding of the role of MQC in CKD, thereby providing effective and feasible treatment strategies and intervention methods for CKD.

While this review offers a comprehensive overview of research progress in targeting the MQC system for CKD treatment, several critical limitations persist. Firstly, clinical evidence is notably insufficient. Owing to the current constraints of existing research, investigations into both individual CBD metabolites and formulation remain confined to preclinical stages (e.g., *in vitro* cell models and *in vivo* animal studies). Thus, well-designed, multicenter clinical trials are urgently needed to validate their translational potential moving forward. Secondly, despite the complex compositions of herbal formulations (e.g., Liuwei Dihuang Wan, Zhenwu Decoction), the review merely summarizes their overall therapeutic effects and associated signaling pathways. It does not delve into the synergistic mechanisms of core bioactive metabolites (e.g., polysaccharides, saponins, alkaloids) nor elucidate inter-metabolite interactions—areas where techniques such as network pharmacology and metabolomics could provide critical mechanistic insights. Future work must address these gaps to fully unravel the therapeutic potential of herbal formulations. Lastly, pharmacokinetic profiles and bioavailability of CBD metabolites remain inadequately characterized. Key challenges—including low oral bioavailability, rapid metabolic clearance, and poor renal targeting of CBD metabolites (particularly polyphenols and polysaccharides)—pose major bottlenecks to successful clinical translation. To overcome these barriers, future efforts should focus on three priority areas: (1) Synthesizing existing pharmacokinetic research on CBD metabolites, including detailed absorption, distribution, metabolism, and excretion (ADME) profiles; (2) Analyzing critical factors influencing bioavailability, such as metabolic modification by gut microbiota; (3) Exploring advancements in delivery system optimization, including nanocarriers, liposomes, and kidney-specific targeting vectors. Addressing these issues will elevate the management of CKD through CBD to a higher level.

In conclusion, mitochondria are essential organelles that regulate the health of the body. In recent years, with the continuous deepening of research on the structure and function of cellular mitochondria, including the communication mechanisms between mitochondria and the cell nucleus, and the mechanism of action between the programmed cell death pathway and mitochondrial damage, future in-depth exploration utilizing MQC as a starting point will be conducive to revealing the pathological mechanism of CKD, providing new targets and new strategies for the prevention and treatment of CKD and the advancement of CBD.

## Glossary

AGE: advanced glycation end product
AHR: Aryl hydrocarbon receptor
AIF: apoptosis-inducing factor
AKI: acute kidney injury
AMPK: adenosine 5′-monophosphate-activated protein kinase
Apaf-1: apoptotic protease activating factor-1
ATG5: autophagy related 5
Bcl-2: B-cell lymphoma-2
BNIP3: Bcl-2 interacting protein 3
BUN: blood urea nitrogen
CAN: chronic allograft nephropathy
CAT: catalase
Caspase-3/7/8/9: cysteine aspartate-specific protease-3/7/8/9
CBD: Chinese botanical drugs
CDH6: cadherin 6
CIN: contrast-associated nephropathy
CKD: chronic kidney disease
CRF: chronic renal failure
CXCR4: CXC chemokine receptor 4
Cyt C: cytochrome C
DKD: diabetic kidney disease
DN: diabetic nephropathy
Drp1: dynamin-related protein 1
ECM: extracellular matrix
GEC: glomerular endothelial cel
ERS: endoplasmic reticulum stress
FoXO1: forkhead box O1
FUNDC1: FUN14 domain containing 1
GFR: glomerular filtration rate
GPx: glutathione peroxidase
GSK-3β: glycogen synthase kinase-3
HFD: high fat diet
HIF-1: hypoxia inducible factor-1
HK-2: hexokinase 2
HMOX-1: heme oxygenase
HN: hypertensive nephropathy
IRI: ischemia-reperfusion injury
IS: indole-3-carboxylic acid sulfate
Keap1: Kelch-1ike ECH-associated protein
LPS: lipopolysaccharides
MAMs: mitochondrial associated membranes
MAPK: mitogen-activated protein kinase
Mdivi-1: mitochondrial division inhibitor-1
MFN1: mitofusin 1
MFN2: mitofusin 2
Mn-SOD: Mn-Superoxide dismutase
MMP: mitochondrial membrane potential
MQC: mitochondrial quality control
mtDNA: mitochondrial DNA
mtROS: mitochondrial reactive oxygen species
Nfe2l1: nuclear factor erythroid-2 like 1
NF-κB: nuclear factor kappa-B
NLRP3: nucleotide-binding oligomerization domain
NOX4: NADPH oxidase 4
NQO1: quinone acceptor oxidoreductase
Nrf1: nuclear respiratory factor 1
Nrf2: nuclear respiratory factor 2
OPA1: optic atrophy type 1
OMM: outer mitochondrial membrane
OS: oxidative stress
OXPHOS: oxidative phosphorylation
PCS: p-cresol sulfate
PDGF-C: platelet derived growth factor
PGAM5: phosphoglycerate mutase 5
PGC-1: peroxisome proliferator activated receptor γ coactivator-1
PGRN: progranulin
PINK1: PTEN induced putative kinase 1
PKB: protein kinase B
PKC: protein kinase C
PPARγ: peroxisome proliferator-activated receptor γ
PRC: polycomb repressive complex
RIF: renal interstitial fibrosis
ROS: reactive oxygen species
SCFAs: short-chain fatty acids
Scr: serum creatinine
SDF-1α: stromal cell-derived factor 1α
SDH: succinate dehydrogenase
SHR: spontaneous hypertension rat
SOD: superoxide dismutase
STAT3: signal transducer and activator of transcription 3
TFAM: mitochondrial transcription factor A
TLR4: Toll-like receptor 4
TMAO: trimethylamine N-oxide
TNF-α: tumor necrosis factor-α
TOMM20: translocase of outer mitochondrial membrane 2
TPP: thiamine pyrophosphate
T2DM: type 2 diabetes
UACR: urinary albumin to creatinine ratio
UCP2: uncoupling protein
ULK1: UNC-51-like kinase 1
UUO: unilateral ureteral obstruction
5/6 Nx: 5/6 nephrectomy
