# Supplementary material for: Pharmacological potential of Chinese botanical drugs in managing chronic kidney disease by targeting mitochondrial quality control
Source: Front Pharmacol. 2026 Feb 19;16:1725842. doi: 10.3389/fphar.2025.1725842 (PMC12960602; doi:10.3389/fphar.2025.1725842)
Supplement: Supplementary file 1 [file Supplementaryfile1.docx]

**Identification of studies via database**

Additional records identified through other sources(n=0)

Records identified from Databases (n = 6957)

**Identification**

Records after duplicates removed(n=6869)

Records excluded (n=6194)

Records screened (n = 6869)

**Screening**

Full-text articles excluded, with reasons(n=564)

Full-text articles assessed for eligibility(n=675)

Articles included in review (n=129)

**Included**

The prospective protocol for this review was pre-established, with search terms and inclusion/exclusion criteria carefully selected to ensure comprehensive coverage of all relevant literature. On November 26, 2025, computer-assisted database searches were conducted across the following databases: PubMed, Web of Science, Embase, Scopus, and China National Knowledge Infrastructure (CNKI). The search strategy utilized free-text terms, employing keywords such as ‘chronic kidney disease' OR 'chronic kidney disorder' OR 'chronic kidney insufficiency,' AND 'mitochondria' OR 'mitochondrion',” AND 'Chinese herbal medicine'. External limitations were applied to each database, though specific constraints varied due to differences in search options. Additionally, reference lists of relevant studies were cross-examined to identify additional studies potentially missed by the initial database search.

This search strategy aims to cover literature from the past decade on the use of Chinese herbal medicine targeting mitochondria for the treatment of chronic kidney disease (CKD), followed by article screening based on relevance to the research topic. Initial inclusion criteria were: (i) inclusion of Chinese herbal medicine targeting mitochondria for CKD treatment; (ii) reporting at least one outcome related to: mitochondrial dynamics (fission, fragmentation, fusion, mitophagy, autophagy, biogenesis), mitochondrial uncoupling, mitochondrial membrane potential, respiratory capacity, oxidative stress/damage, or antioxidant capacity. Exclusion criteria were: (i) studies lacking sufficient evidence of mitochondrial dysfunction; (ii) review articles; (iii) meta-analyses; (iv) unpublished studies, abstracts, or case reports; (v) non-peer-reviewed articles, book chapters, conference abstracts, or research protocols. After applying these criteria, a final selection of 104 articles was made (Figure 1).
