# Supplementary material for: Pharmacological potential of Chinese botanical drugs in managing chronic kidney disease by targeting mitochondrial quality control
Source: Front Pharmacol. 2026 Feb 19;16:1725842. doi: 10.3389/fphar.2025.1725842 (PMC12960602; doi:10.3389/fphar.2025.1725842)
Supplement: Supplementary file 2 [file Table1.docx]

**Supplementary Table 1: Mechanism of MQC-targeted natural chemical metabolites for CKD**

| **Targets** | **Categories** | **Natural chemical metabolites** | **Resource** | **Cellular Models** | **Animal Models** | **Dose** | **Duration time** | **Negative/positive control** | **Outcomes** | **Mechanism** | **References** |
| --- | --- | --- | --- | --- | --- | --- | --- | --- | --- | --- | --- |
| Mitochondrial biogenesis | Polyphenols | Resveratrol | *Polygoni Cuspidati Rhizoma et Radix* | SV40 MES 13 | 5/6Nx (rats | Cellular Model: 10 μM; Animal Model:20 mg/kg/day (Intraperitoneal injection) | Cellular Model: 12h; Animal Model: 4 weeks | NC: saline solution; PC: - | Restored mitochondrial function in the kidney and reduced oxidative damage | SIRT1/PGC-1α signaling pathway | (Hui et al., 2017; Wang et al., 2015) |
|  |  | Quercetin | *Cuscuta epithymum (L.) L* | NRK-52E cells (AngII-treated) | UUO (rats) | Cellular Model: 20 μM; Animal Model:100 mg/kg/day | Cellular Model: 24h; Animal Model:6 weeks | NC: -; PC: - | Reduced renal tubular epithelial cell aging and interstitial fibrosis in rats | SIRT1/PINK1/Parkin signaling pathway | (Liu et al., 2020) |
|  |  | Curcumin | *Curcuma L. and Curcuma zedoaria (Christm.) Roscoe.* | C2C12 cells | 5/6Nx (rats | Cellular Model: 5 μM; Animal Model: 20 mg/kg/day | Cellular Model: -; Animal Model: - | NC: -; PC: Mdivi-1（1 μM） | Reduced renal mitochondrial oxidative damage and mitochondrial dysfunction | GSK-3β, Nrf2 and PGC-1α | (Negrette-Guzmán et al., 2015; Wang, D. et al., 2020) |
|  | Glycosides | Astragaloside IV | *Astragalus L.* | HK-2 cells (HG-induced) | STZ (rats) | Cellular Model: 0, 12.5, 25, 50, 100, 200, and 400 μM; Animal Model: 20 mg/kg/day | Cellular Model: 24 h; Animal Model: 6 weeks | NC: -; PC: - | Reduced renal fibrosis | HMOX1/FTH1/TFR1 signaling pathway; SIRT1/PGC-1α/Nrf1 signaling pathway | (Li et al., 2024; Liu et al., 2024) |
|  |  | Ginsenoside Rg2 | *Panax ginseng C. A. Mey.* | - | SAMP8 mice | - | 8 weeks | NC: 0.05 % CMC-Na; PC: - | Alleviated age-related kidney damage | IGF-1/mTOR and PI3K / AKT signaling pathway | (Tsopmejio et al., 2025) |
|  |  | Salidroside | *Rhodiola rosea L.* | - | STZ (mice) | 50 and 100 mg/kg/day | 10 weeks | NC: 0.05 % CMC-Na; PC: Metformin (0.3 mg/ml or 0.6 mg/ml) | Reduced proteinuria formation and alleviated renal fibrosis | SIRT1/PGC-1α signaling pathway | (Wu et al., 2016; Xue et al., 2019) |
|  | Other categories | Berberine | *Coptis chinensis Franch.* | Immortalized mouse podocytes | db/db (mice) | Cellular Model: 0.4 μM; Animal Model: 200 and 300 mg/kg/day | Cellular Model: 12 h; Animal Model: 8 weeks | NC: -; PC: - | Reversed metabolic disorders, podocyte damage, and glomerulosclerosis | AMPK and PGC-1α | (Qin et al., 2020) |
|  |  | Grape seed proanthocyanidin extracts | Widely found in fruits and vegetables | - | HFD (rats) | 125,250, and 500 mg/kg/day (oral gavage) | 16 weeks | NC: -; PC: - | Improved podocell damage in DN | AMPK/SIRT 1/PGC-1α signaling pathway | (Bao et al., 2014) |
|  |  | Glycyrrhizic acid | *Glycyrrhiza glabra L.* | NRK-52E cells (HG-induced) | - | - | 12 or 48 h | NC: -; PC: - | Protected renal tubular epithelial cells | AMPK and SIRT1 | (Hou et al., 2014) |
|  |  | Morroniside | *Corni Fructus* | mRTECs (HG-induced) | - | 6.25, 12.5, 25,  50, and 100 μM | 24 h | NC: -; PC: - | Improved damage to renal tubular epithelial cells (mRTECs) in mice | PGC-1/LXR signaling pathway | (Gao et al., 2021) |
|  |  | Echinochrome A | *Echinoidea* | - | db/db (mice) | 3 mg/kg/day | 12 weeks | NC: Saline solution; PC: - | Enhanced glycemic control, improved kidney health (lower BUN, SCr levels, decreased renal fibrosis); Reduced OS, increased ATP production; Inhibited PKC-iota/p38 MAP, and upregulated AMPK/Nrf2/HO-1, promoted PGC-1α expression | PKC-iota/p38 MAPK signaling pathway,  NOX4, TGF-β1 signaling pathway, and AMPK/  Nrf2/HO-1, PGC-1α signaling pathway | (Pham et al., 2023) |
| Mitochondrial oxidative stress | Polyphenols | Curcumin | *Curcuma L. and Curcuma zedoaria (Christm.) Roscoe.* | NRK-52E cells (HG-induced) | - | 0, 5, 10, or 20 μM | 24 h | NC: -; PC: - | Inhibiting DKD renal fibrosis and protecting renal function | TLR4/NF-κB signaling pathway | (Liu, X. et al., 2021) |
|  |  | Resveratrol | *Polygoni Cuspidati Rhizoma et Radix* | Podocytes (HG-induced) | STZ (mice) | Cellular Model: -; Animal Model: 30 mg/kg/day | Cellular Model: -; Animal Model: 4, 8, and 12 weeks | NC: -; PC: - | Inhibited renal fibrosis, protected renal function, and improved podocyte damage in diabetic mice | Nrf2 /Keap1 signaling pathway and SIRT1/PGC-1α and SIRT3 signaling pathway | (Zhang, T. et al., 2019) |
|  |  | Chicoric acid | *Cichorium intybus L.* | - | HFD (mice) | 15 and 30 mg/kg/day (oral gavage) | - | NC: -; PC: - | Reduced mtROS; Increased MMP | p62, Mfn2 and Fis1; Nrf2/PINK signaling pathway and Parkin/LC3 signaling pathway | (Ding, X.-q. et al., 2022) |
|  | Glycosides | Astragaloside IV | *Astragalus L.* | Podocytes (HG-induced) | - | Cellular Model: 0, 0.3, 1.0, 3.0, 10, 20, 40, 80, 100 μM; Animal Model: 6 mg/kg/day (oral gavage) | Cellular Model:24 h; Animal Model:10 weeks | NC: -; PC: Trigonelline 25 mg/kg/d (oral gavage) | Reduced renal injury and podocyte apoptosis | Nrf2-ARE/TFAM signaling pathway | (Shen et al., 2023) |
|  |  | Acteoside-containing caffeic acid and isoacteoside | *Cistanche deserticola Ma* | NRK-52E cells | Adenine-induced (rats) | Cellular Model: 0, 1, 10, 20, 40, 80, 100,200 μM; Animal Model: 20 mg/kg/day (oral gavage) | Cellular Model:24 h; Animal Model:3 weeks | NC: -; PC: - | Mitigated tubulointerstitial fibrosis | AHR signaling; NF-κB/Nrf2 signaling axis | (Wang et al., 2025) |
|  |  | Geniposidic Acid | *Ixora chinensis Lam.* | NRK-52E cells | Adenine-induced (rats) | Cellular Model: 0, 1, 10, 20, 40, 80, 100,  and 200μM; Animal Model: 20 mg/kg/day (oral gavage) | Cellular Model:24 h; Animal Model:3 weeks | NC: -; PC: - | Mitigated renal fibrosis | AHR signaling; NF-ƙB and Nrf2 pathways | (Wang, Y.N. et al., 2024) |
|  |  | Notoginsenoside R1 | *Panax notoginseng (Burkill) F.H.Chen* | HK-2 cells (AGE-induced) | db/db (mice) | Cellular Model: -; Animal Model: 30 mg/kg/day (oral gavage) | Cellular Model:24 h; Animal Model:20 weeks | NC: -; PC: Metformin 200 mg/kg/day (oral gavage) | Relieved HK-2 cell apoptosis and renal fibrosis | Nrf2/ HO-1 signaling pathway | (Zhang, B. et al., 2019) |
|  |  | Schisandrin B | *Fructus Schisandrae Chinensis* | HK-2 cells (HG-induced) | db/db (mice) | Cellular Model: 40 μM; Animal Model: 20 mg/kg/day (oral gavage) | Cellular Model:48 h; Animal Model:6 weeks | NC: CMC-Na; PC: - | Alleviated epithelial-mesenchymal transformation of renal tubular cells in DKD mice | Akt /AMPK signaling pathway | (Liu et al., 2023) |
|  |  | Naringin | *Citrus paradisi Macfad* | - | STZ (rats) | 40 mg/kg/day (oral gavage) | 30 days | NC: -; PC: - | Prevented renal injury (SCr, urine protein/creatinine ratio, serum albumin, serum total proteins) with partial renal morphological recovery; Prevented OS and preserved ATP synthase activity. | mtROS | (Pérez et al., 2023) |
|  | Flavonoids | Quercetin | *Cuscuta epithymum (L.) L* | HK-2 (HG-induced) | db/db (mice) | Cellular Model: 0, 5, 10, 15, 25, and 50µM; Animal Model: 100 mg/kg/day (oral gavage) | Cellular Model:48 h; Animal Model:12 weeks | NC: Saline solution; PC: Ferrostain-1 (10μM or 5mg/  kg) (oral gavage) | Relieved podocyte dedifferentiation and reduced urine protein in DKD mice | Notch and Nrf2/PINK1 signaling pathway | (Zhang et al., 2024; Zhu et al., 2024) |
|  |  | Astragalin | *Astragalus L.* | HK-2 cells (HG-induced, PA-induced) | STZ  (mice) | Cellular Model: 20 µM; Animal Model: 5 and 10 mg/kg/day (Intraperitoneal injection) | Cellular Model:12 or h; Animal Model:4 weeks | NC: 0.5% CMC-Na (oral gavage); PC: MitoQ (1 μM) or Epalrestat group (40 mg/kg) (Intraperitoneal injection) | Improved renal function (SCr, urine ACR, proteinuria, renal pathological changes) | AMPK/PGC-1α signaling pathway | (Sun et al., 2023) |
|  |  | Baicalin | *Scutellaria baicalensis Georg* | - | STZ (rats) | 120 mg/kg/day | 30 days | NC: -; PC: Metformin 200 mg/kg/day | Improved OS and inhibited inflammatory responses | SIRT1 /HO-1 and MAPK signaling pathway | (De Oliveira et al., 2015) |
|  | Polysaccharides | Astragalus polysaccharides | *Angelica sinensis (Oliv.) Diels.* | - | STZ (rats) | 200, 400, and 800 mg/kg/day (oral gavage) | 4 weeks | NC: Citrate buffer; PC: Metformin (oral gavage) | Increased the MMP of podocytes and reduced the apoptosis rate of podocytes | TLR 4/NF-κB signaling pathway | (Guo et al., 2023) |
|  |  | Fucoidan | *Sporophylls* | - | HFD-induced  (mice) | 50, 100, and 200 mg/kg/day (oral gavage) | 24 weeks | NC: 0.5% CMC-Na  solution; PC: Metformin (150 mg/kg/day) (oral gavage) | Improved renal function (GFR,  urine creatinine, reduced interstitial fibrosis) | MAPK signaling pathway | (Zhong et al., 2024) |
|  | Terpenoids | Ginsenoside Rb1 | *Panax notoginseng (Burkill) F.H.Chen* | MPCs (HG-induced) | STZ (mice) | Cellular Model: -; Animal Model: 5 and 40 mg/kg/day (oral gavage) | Cellular Model:72 h; Animal Model:7 weeks | NC: 0.1 M Citrate buffer; PC: - | Reduce glomerular damage and reduce expression of apoptotic proteins | Nrf2/HO-1 signaling pathway | (He et al., 2022) |
|  |  | Poricoic acid A | *Poria cocos (Schw.) Wolf* | MPC-5(HG-induced) | STZ (mice) | Cellular Model: 3.125, 6.25, 12.5, 25, 50, 100, 200 µM; Animal Model: 10 and 20 mg/kg/day (oral gavage) | Cellular Model:24 h; Animal Model:4 weeks | NC: 0.5% CMC-Na; PC: - | Inhibited renal fibrosis | LC3 and FUNDC1 | (Wu et al., 2023) |
|  |  | Triptolide | *Tripterygium wilfordii Hook.f.* | HK-2 cells (HG-induced) | HFD (Rats) | Cellular Model: 5 ng/mL; Animal Model: 10 and 200 μg/kg/day (oral gavage) | Cellular Model: 48 h; Animal Model:12 weeks | NC: Saline solution; PC: - | Reduced proteinuria, improved kidney structure, and inhibited renal interstitial fibrosis | GPX4, SLC7A11 and Nrf; PI3K/AKT signaling pathway | (Wang, H.-Q. et al., 2024; Xue et al., 2018) |
|  | Other categories | Paeoniflorin | *Paeonia suffruticosa Andr.* | C2C12 cells | 5/6Nx  (rats) | Cellular Model: 25 and 50 μM; Animal Model: 25 and 50 mg/kg/day (oral gavage) | Cellular Model: 48 h; Animal Model:7 weeks | NC: -; PC: - | Improved renal function and interstitial fibrosis in 5/6Nx rats | AMPK/SIRT1/PGC-1α signaling pathway | (Li, Q. et al., 2022) |
|  |  | Tetramethylpyrazine Nitrone | *A novel nitroxone derivative of TMP* | HK-2 cells | STZ (mice) | Cellular Model: 30 μM; Animal Model: 10, 30, or  60 mg/kg/day | Cellular Model: 24 h; Animal Model:6 weeks (twice daily) | NC: 0.1 M Citrate buffer; PC: losartan (10 mg/kg/day) | Improved renal tissue pathology changes | AMPK/PGC-1α signaling pathway | (Jing et al., 2021) |
|  |  | Jujuboside A | *Ziziphus jujuba Mill.* | - | STZ (rats) | 20 mg/kg/day (oral gavage) | 8 weeks | NC: -; PC: Metformin (300 mg/kg/day) (oral gavage) | Improved renal function in a rat model of T2 DM | NOX4; CaMKK 2-AMPK/p-mTOR and PINK1/Parkin signaling pathway | (Zhong et al., 2022b) |
|  |  | Taurine | *Bos taurus domesticus Gmelin* | MPC 5 (HG-induced) | STZ (mice) | Cellular Model: -; Animal Model: - | Cellular Model: -; Animal Model:12 weeks | NC: -; PC: - | Reduced HG-induced damage to podocytes | CSE/TRPC6 axis | (Zhang et al., 2020) |
|  |  | Astaxanthin | *H. pluvialis* | NHMCs (HG-induced) | - | 10^-4^ or 10^-5^ M | 48 h | NC: -; PC: - | Inhibited the progression of DKD | Nrf2/ARE signaling pathway | (Manabe et al., 2008) |
| Mitophagy | Flavonoids | Icariin | *Epimedium brevicornu Maxim.* | MPC 5(HG-induced) | STZ (rats) | Cellular Model: 1, 3,  10 μM); Animal Model: 20, 40, and 80 mg/kg/day | Cellular Model: 48 h; Animal Model:8 weeks | NC: -; PC: Losartan (13.5 mg/kg/day) | Inhibition of NLRP3 inflammasome activation in rats with DN | Keap1/Nrf2/HO-1 signaling pathway | (Ding, X. et al., 2022) |
|  |  | Puerarin | *Puerariae Lobatae Radix* | Podocytes (HG-induced) | STZ (mice) | Cellular Model: -; Animal Model: 5, 10, 20, or 40 mg/kg/day (oral gavage) | Cellular Model: -; Animal Model:12 weeks | NC: -; PC: - | Protected podocytes from damage | HMOX-1 and SIRT1 | (Li et al., 2020) |
|  | Polysaccharides | Dendrobium officinale polysaccharides | *Dendrobium nobile Lindl.* | MPC 5(HG-induced) | - | 0, 5, 10, 25, 50, 100, and  250 μg/ml | 24 h | NC: -; PC: - | Prevented damage to podocytes | IRS-1/AKT signaling pathway | (Li et al., 2023) |
|  | Glycosides | Astragaloside IV | *Astragalus L.* | - | db/db (mice) | 1 g/kg/day (oral gavage) | 12 weeks | NC: -; PC: - | Protection of damaged kidney cells | PINK1/Parkin signaling pathway | (Liu et al., 2017) |
|  |  | Astragaloside II | *Astragali Radix* | - | STZ (rats) | 3.2 or 6.4 mg/kg/day (oral gavage) | 9 weeks | NC: -; PC: Losartan (10 mg/kg/day | Improved the antioxidant stress resistance of podocytes | Nrf2/PINK1 signaling pathway | (Su et al., 2021) |
|  |  | Loganin | *Corni Fructus* | HK-2 cells (PPVI treated) | HFD/STZ (mice) | Cellular Model: 20 and 5 μM; Animal Model: 5, 10, 50, and 100 mg/kg/day (Intraperitoneal injection) | Cellular Model: 48 h; Animal Model:8 weeks | NC: -; PC: Irbesartan (20 mg/kg/day) (Intraperitoneal injection) | Improved kidney fibrosis and DKD lesions | NLRP3/Caspase-1//Gasdermin D (GSDMD) signaling pathway | (Kong et al., 2023) |
|  |  | Catalpol | *Rehmanniae Radix* | C2C12 cells | STZ (mice) | 100 and 200 mg/kg/day (oral gavage) | 4 weeks | NC: -; PC: - | Improved kidney fibrosis | Nrf2/Pink1 signaling pathway | (Bhattamisra et al., 2021) |
|  |  | Dioscin | *Trigonella foenum-graecum L.* | - | STZ (rats) | 20 mg/kg/day (oral gavage) | 8 weeks | NC: -; PC: Metformin (300 mg/kg/day) (oral gavage) | Enhanced autophagy via AMPKmTOR signaling pathway; Enhanced mitophagy and mitochondrial dynamics; Reduced OS,  enhanced antioxidant enzymes (SOD, CAT), reduced inflammatory markers (IL-1β, IL-6, TNF-α, and NF-κB) | Drp1, p-Drp1 and Mfn2; AMPK/mTOR and Parkin/ PINK1 signaling pathway | (Zhong et al., 2022a) |
|  | Other categories | Emodin | *Rheum rhaponticum L.* | - | STZ (rats) | 20 mg/kg and 40 mg/ kg/day (oral gavage) | 8 weeks | NC: -; PC: - | Reduced proteinuria and relieved renal fibrosis | AMPK/mTOR signaling pathway | (Liu, H. et al., 2021) |
|  |  | Germacrone | *Curcuma aromatica Salisb.* | HK-2 (HG-induced) | db/db  (mice) | Cellular Model: 50 μM; Animal Model: 5, 10, 30, or 60 mg/kg/day (oral gavage) | Cellular Model: 72 h; Animal Model:13 weeks | NC: Saline solution; PC: Ferrostatin-1 (1 μM) | Reversed mitophagy inhibition (restored p62, TOM20, Tim23, PINK1, Parkin, LC3II/I, COX IV, FTH1 expression levels); Prevented mtDNA release, 8-OHdG, and cGAMP increases | p62, TOM20, Tim23,  PINK1, Parkin, LC3II/I, COX IV and FTH1 | (Wang et al., 2023) |
| Mitochondrial dynamics | Polyphenols | Epigallocatechin gallate | *Green Tea* | - | Kidney injury mouse model | Day 1: -6.25, 12.5and 25 mg/kg/day; Days 2 to 3: 25 mg/kg/day | 3 days | NC: -; PC: - | Protected against kidney damage | Drp1 | (Chu et al., 2024) |
|  | Glycosides | Icariin | *Epimedii Folium* | NRK-52 E cells (HG-induced) | STZ (rats)；5/6Nx  (rats) | Cellular Model: 1, 3, and 10 μM; Animal Model: 20, 40, 80 mg/kg/day (Intraperitoneal injection) | Cellular Model: 48 h; Animal Model:9 weeks | NC: Saline solution; PC: Irbesartan (13.5 mg/kg/day) (Intraperitoneal injection) | Relieved renal fibrosis | Drp1; Nrf2/Keap 1 signaling pathway | (Jia et al., 2021; Wang, K. et al., 2020; Wang et al., 2022; Zhang et al., 2017) |
|  | Flavonoids | Formononetin | *Puerariae Lobatae Radix, Astragali Radix, and Callerya reticulata (Benth.) Schot* | HK-2 (HG-induced) | FA (rats)； STZ (rats) | Cellular Model: 10 and 30 μM; Animal Model: 20 mg/kg/day (oral gavage) | Cellular Model: 48 h; Animal Model:8 weeks | NC: 0.5% Methylcellulose solution; PC: losartan (10 mg/kg/day) (oral gavage) | Alleviated renal tubular damage and mitochondrial damage in DN | Smad 3/ATF 3/SLC 7A 11 signaling pathway; SIRT 1/PGC-1α signaling pathway | (Huang et al., 2022; Zhu et al., 2023) |
|  |  | Silybin | *Silybum marianum (L.) Gaertn.* | HK 2 (HG-induced) | Cisplatin-induced AKI (mice) | Cellular Model: 50 μM; Animal Model: 200 mg/kg/day (oral gavage) | Cellular Model: 24 h; Animal Model:10 days | NC: -; PC: - | Reduced renal tubular cell apoptosis and improved cell regeneration | SIRT3 and Drp1; MAPK signaling pathway | (Li et al., 2017; Yang et al., 2022; You et al., 2020) |
|  |  | Genistein | *Cytisus scoparius (L.) Link* | - | STZ (rats) | 30 and 50 mg/kg/day (oral gavage) | 6 weeks | NC: -; PC: - | Improved renal function (SCr, BUN, 24 h urine protein, renal pathological lesions) | Mfn2; NF-κB/p53 MAPK signaling pathway | (Li, Y. et al., 2022) |
|  | Terpenoids | Asiatic acid | *Centella asiatica (L.) Urb.* | HK-2 (HG-induced) | STZ (rats) | Cellular Model: 10 and 20 μM; Animal Model: 10 and 30 mg/kg/day (oral gavage) | Cellular Model: 24 h; Animal Model:10 weeks | NC: 0.5% sodium carboxyl methylcellulose; PC: Bardoxolone methyl (200 μg/ml) | Prevented renal tubular damage and mitochondrial damage | Nrf2 | (Ji et al., 2023) |
|  | Other categories | Tetramethylpyrazine | *Chuanxiong Rhizoma* | NRK-52 E (HO-1 overexpression) | Gentamicin-induced AKI (mice) | Cellular Model: 1, 10, 50, and 100μM; Animal Model: 80 mg/kg/day (Intraperitoneal injection) | Cellular Model: 12 h; Animal Model:4 days | NC: Saline solution; PC: - | Improved renal oxidative stress and abnormal mitochondrial dynamics, and regulated mitophagy in renal tubular cells | HO-1/HAX-1 signaling pathway; CCL 2/CCR 2 signaling pathway | (Gong et al., 2019; Sue et al., 2009) |
| Mitochondrial apoptosis | Glycosides | Notoginsenoside R1 | *Panax notoginseng (Burkill) F.H.Chen* | - | STZ (rats) | 5, 10, and 20 mg/kg/day (oral gavage) | 16 weeks | NC: -; PC: - | Protected podocytes from damage | PI3K/Akt/mTOR signaling pathway | (Huang et al., 2016) |
|  |  | Cornuside | *Corni Fructus* | Rat podocytes (HG-induced) | STZ (rats) | Cellular Model: 10μM; Animal Model: 100 mg/kg/day (oral gavage) | Cellular Model: 24 h; Animal Model:8 weeks | NC: -; PC: - | Alleviated renal damage in DN rats | pERK/ATF4/CHOP signaling pathway | (Gao et al., 2025; Ma et al., 2014; Xiang et al., 2025) |
|  | Flavonoids | Mangiferin | *Mangifera indica* | - | STZ (mice and rats) | 40 mg/kg/day (oral gavage) | 30 days | NC: -; PC: - | Protected kidney cells from apoptosis | NF-κB /TGF-β1 signaling pathway; Bax and Bcl-2 signaling pathway; Nox 4 oxidase signaling pathway | (Guang-Kai et al., 2017; Pal et al., 2014) |
|  | Other categories | Salvianolic acid B | *Salvia miltiorrhiza Bunge* | HBZY- 1 and iCell-r013 (HG-induced) | SZT (rats); UUO (mice); STZ (rats) | Cellular Model: 0.1, 1, and 10 μM; Animal Model: 50, 100, and 200 mg/kg/day (oral gavage) | Cellular Model: 48 h; Animal Model:6 weeks | NC: -; PC: - | Reduced the incidence of kidney inflammation and fibrosis | PDGF-C/PDGFR-α signaling pathway; SIRT 3/FOXO 1 signaling pathway | (Wen et al., 2023; Yao et al., 2022) |
| **Gut-kidney axis** | Polysaccharides | Moutan Cortex polysaccharide | *Paeonia suffruticosa Andr* | - | STZ (rats) | 80 and 160 mg/kg/day (oral gavage) | 12 weeks | NC: -; PC: Aminoguanidine (100 mg/kg/day) (oral gavage) | Regulated gut microbiota, improved intestinal barrier function, corrected metabolic imbalances, and reduced systemic inflammation. | *Akkermansia*, *Muribaculaceae,* and *Lactobacillus* | (Zhang et al., 2022) |
|  | Quinones | Nanoparticle-Mediated Delivery of Emodin | *Rheum rhaponticum L.* | - | 5/6 Nx (rats) | 1.15 and 4.6mg/kg/2 days | 8 weeks | NC: -; PC: - | Modulated gut microbiota and delays  CKD progression | IL-1β, IL-6, and LPS | (Lu et al., 2021) |
|  | Flavonoids | Total Flavones of *Abelmoschus manihot* | *Abelmoschus manihot (L.)Medic* | RAW264.7 cells | 5/6 Nx (rats) | Cellular Model: 10, 20, and 40 μg/ml; Animal Model:136 mg/kg/day | Cellular Model: -; Animal Model: 2 to 11 weeks | NC: -; PC: Febuxostat (4.11 mg/kg/ day) | Restored gut microbiota dysbiosis and suppressing gut metabolism-derived microinflammation to exert its renal protective effects | AMPK/SIRT1 signaling pathway | (Tu et al., 2020) |
|  |  | Fisetin | *Rhus succedanea L* | Mouserenal tubular epithelial cells | Adenine-induced (mice) | Cellular Model: -; Animal Model: 50 and 100 mg/kg/day | Cellular Model: -; Animal Model:28 days | NC: -; PC: Allopurinol and Benzbromarone | Alleviated kidney injury and renal fibrosis in mice of  hyperuricemia-induced CKD | Aryl hydrocarbon receptor (AHR) | (Ren et al., 2021) |
|  | Polyphenols | Resveratrol | *Polygoni Cuspidati Rhizoma et Radix* | IEC-6 intestinal cell line | 5/6 Nx (mice) | Cellular Model: 10 μM; Animal Model: 30 mg/kg/day | Cellular Model: -; Animal Model:4 weeks | NC: -; PC: - | Ameliorate gut-renal axis disturbances in CKD | PI3K/Akt/HO-1 signaling pathway and mTOR | (Zheng et al., 2024) |
|  |  | Curcumin | *Curcuma L. and Curcuma zedoaria (Christm.) Roscoe.* | - | Adenine-induced (rats) | 200 mg/kg/day | 8 weeks | NC: -; PC: - | Reduced uremic toxin levels and improved renal inflammation and fibrosis | Beneficial bacteria (e.g., *Lactobacillus* and *Ruminococcaceae*) and pathogenic bacteria (e.g., *Bacteroides*, *Lachnospiraceae*, and *Escherichia-Shigella*) | (Xu et al., 2021) |
|  |  | Docosahexaenoic acid-acylated curcumin diester | *Curcuma L.* and *Curcuma zedoaria (Christm.) Roscoe*. | - | AKI (mice) | 350 mg/kg/day | 7 days | NC: -; PC: - | Reduced the degree of renal tubular injury | PI3K/Akt/NF-κB signaling pathway |  |
|  | Saponin | Panax notoginseng saponins | *Panax notoginseng* | - | Adenine-induced (mice) | 40, 80, and 160 mg/kg/day(oral gavage) | 28 days | NC: -; PC: Valsartan (13 mg/kg/d) (oral gavage) | Inhibited the development of inflammation and fibrosis in the kidney tissue | α-SMA, SMAD3, Occludin and ZO-1 | (Xie et al., 2022) |

**References**

Bao, L., Cai, X., Dai, X., Ding, Y., Jiang, Y., Li, Y., et al.(2014). Grape seed proanthocyanidin extracts ameliorate podocyte injury by activating peroxisome proliferator-activated receptor-γ coactivator 1α in low-dose streptozotocin-and high-carbohydrate/high-fat diet-induced diabetic rats [J]. *Food Funct*, 5(8): 1872-1880.<http://dx.doi.org/10.1039/c4fo00340c>

Bhattamisra, S.K., Koh, H.M., Lim, S.Y., Choudhury, H., Pandey, M.(2021). Molecular and biochemical pathways of catalpol in alleviating diabetes mellitus and its complications [J]. *Biomolecules*, 11(2): 323.<http://dx.doi.org/10.3390/biom11020323>

Chu, Y.-L., Pi, J.-C., Yao, Y.-F., Chen, X.-Y., Peng, X.-P., Li, W.-J.(2024). Polyphenol (−)-Epigallocatechin Gallate (EGCG) mitigated kidney injury by regulating metabolic homeostasis and mitochondrial dynamics involvement with Drp1-mediated mitochondrial fission in mice [J]. *Food Chem Toxicol*, 191: 114906.<http://dx.doi.org/10.1016/j.fct.2024.114906>

De Oliveira, M.R., Nabavi, S.F., Habtemariam, S., Orhan, I.E., Daglia, M., Nabavi, S.M.(2015). The effects of baicalein and baicalin on mitochondrial function and dynamics: A review [J]. *Pharmacol Res*, 100: 296-308.<http://dx.doi.org/10.1016/j.phrs.2015.08.021>

Ding, X.-q., Jian, T.-y., Gai, Y.-n., Niu, G.-t., Liu, Y., Meng, X.-h., et al.(2022). Chicoric acid attenuated renal tubular injury in HFD-induced chronic kidney disease mice through the promotion of mitophagy via the Nrf2/PINK/Parkin pathway [J]. *J Agric Food Chem*, 70(9): 2923-2935.<http://dx.doi.org/10.1021/acs.jafc.1c07795>

Ding, X., Zhao, H., Qiao, C.(2022). Icariin protects podocytes from NLRP3 activation by Sesn2-induced mitophagy through the Keap1-Nrf2/HO-1 axis in diabetic nephropathy [J]. *Phytomedicine*, 99: 154005.<http://dx.doi.org/10.1016/j.phymed.2022.154005>

Gao, G., Su, X., Liu, S., Wang, P., Chen, J.J., Liu, T., et al.(2025). Cornuside as a promising therapeutic agent for diabetic kidney disease: Targeting regulation of Ca2+ disorder-mediated renal tubular epithelial cells apoptosis [J]. *Int Immunopharmacol*, 149: 114190.<http://dx.doi.org/10.1016/j.intimp.2025.114190>

Gao, J., Liu, P., Shen, Z., Xu, K., Wu, C., Tian, F., et al.(2021). Morroniside Promotes PGC‐1α‐Mediated Cholesterol Efflux in Sodium Palmitate or High Glucose‐Induced Mouse Renal Tubular Epithelial Cells [J]. *Biomed Res Int*, 2021(1): 9942152.<http://dx.doi.org/10.1155/2021/9942152>

Gong, X., Duan, Y., Zheng, J., Ye, Z., Hei, T.K.(2019). Tetramethylpyrazine prevents contrast‐induced nephropathy via modulating tubular cell mitophagy and suppressing mitochondrial fragmentation, CCL2/CCR2‐mediated inflammation, and intestinal injury [J]. *Oxid Med Cell Longevity*, 2019(1): 7096912.<http://dx.doi.org/10.1155/2019/7096912>

Guang-Kai, X., Chen-Yu, S., Xiao-Ying, Q., Yu, H., Yi, L., Guo-Yong, X., et al.(2017). Effects of ethanol extract of Bombax ceiba leaves and its main constituent mangiferin on diabetic nephropathy in mice [J]. *Chin J Nat Med*, 15(8): 597-605.<http://dx.doi.org/10.1016/S1875-5364(17)30087-0>

Guo, M., Gao, J., Jiang, L., Dai, Y.(2023). Astragalus polysaccharide ameliorates renal inflammatory responses in a diabetic nephropathy by suppressing the TLR4/NF-κB pathway [J]. *Drug Des Devel Ther*: 2107-2118.<http://dx.doi.org/10.2147/DDDT.S411211>

He, J.-y., Hong, Q., Chen, B.-x., Cui, S.-y., Liu, R., Cai, G.-y., et al.(2022). Ginsenoside Rb1 alleviates diabetic kidney podocyte injury by inhibiting aldose reductase activity [J]. *Acta Pharmacol Sin*, 43(2): 342-353.<http://dx.doi.org/10.1038/s41401-021-00788-0>

Hou, S., Zheng, F., Li, Y., Gao, L., Zhang, J.(2014). The protective effect of glycyrrhizic acid on renal tubular epithelial cell injury induced by high glucose [J]. *Int J Mol Sci*, 15(9): 15026-15043.<http://dx.doi.org/10.3390/ijms150915026>

Huang, G., Lv, J., Li, T., Huai, G., Li, X., Xiang, S., et al.(2016). Notoginsenoside R1 ameliorates podocyte injury in rats with diabetic nephropathy by activating the PI3K/Akt signaling pathway [J]. *Int J Mol Med*, 38(4): 1179-1189.<http://dx.doi.org/10.3892/ijmm.2016.2713>

Huang, Q., Chen, H., Yin, K., Shen, Y., Lin, K., Guo, X., et al.(2022). Formononetin attenuates renal tubular injury and mitochondrial damage in diabetic nephropathy partly via regulating Sirt1/PGC-1α pathway [J]. *Front Pharmacol*, 13: 901234.<http://dx.doi.org/10.3389/fphar.2022.901234>

Hui, Y., Lu, M., Han, Y., Zhou, H., Liu, W., Li, L., et al.(2017). Resveratrol improves mitochondrial function in the remnant kidney from 5/6 nephrectomized rats [J]. *Acta Histochem*, 119(4): 392-399.<http://dx.doi.org/10.1016/j.acthis.2017.04.002>

Ji, Y., Zhang, X., Chen, J., Song, S., Fang, S., Wang, Z., et al.(2023). Asiatic acid attenuates tubular injury in diabetic kidney disease by regulating mitochondrial dynamics via the Nrf-2 pathway [J]. *Phytomedicine*, 109: 154552.<http://dx.doi.org/10.1016/j.phymed.2022.154552>

Jia, Z., Wang, K., Zhang, Y., Duan, Y., Xiao, K., Liu, S., et al.(2021). Icariin ameliorates diabetic renal tubulointerstitial fibrosis by restoring autophagy via regulation of the miR-192-5p/GLP-1R pathway [J]. *Front Pharmacol*, 12: 720387.<http://dx.doi.org/10.3389/fphar.2021.720387>

Jing, M., Cen, Y., Gao, F., Wang, T., Jiang, J., Jian, Q., et al.(2021). Nephroprotective effects of tetramethylpyrazine nitrone TBN in diabetic kidney disease [J]. *Front Pharmacol*, 12: 680336.<http://dx.doi.org/10.3389/fphar.2021.680336>

Kong, X., Zhao, Y., Wang, X., Yu, Y., Meng, Y., Yan, G., et al.(2023). Loganin reduces diabetic kidney injury by inhibiting the activation of NLRP3 inflammasome-mediated pyroptosis [J]. *Chem Biol Interact*, 382: 110640.<http://dx.doi.org/10.1016/j.cbi.2023.110640>

Li, H., Zheng, J., Wu, Y., Zhou, H., Zeng, S., Li, Q.(2023). Dendrobium officinale polysaccharide decreases podocyte injury in diabetic nephropathy by regulating IRS-1/AKT signal and promoting mitophagy [J]. *Aging*, 15(19): 10291.<http://dx.doi.org/10.18632/aging.205075>

Li, L., Zou, J., Zhou, M., Li, H., Zhou, T., Liu, X., et al.(2024). Phenylsulfate-induced oxidative stress and mitochondrial dysfunction in podocytes are ameliorated by Astragaloside IV activation of the SIRT1/PGC1α/Nrf1 signaling pathway [J]. *Biomed Pharmacother*, 177: 117008.<http://dx.doi.org/10.1016/j.biopha.2024.117008>

Li, Q., Wu, J., Huang, J., Hu, R., You, H., Liu, L., et al.(2022). Paeoniflorin ameliorates skeletal muscle atrophy in chronic kidney disease via AMPK/SIRT1/PGC-1α-mediated oxidative stress and mitochondrial dysfunction [J]. *Front Pharmacol*, 13: 859723.<http://dx.doi.org/10.3389/fphar.2022.859723>

Li, X., Zhu, Q., Zheng, R., Yan, J., Wei, M., Fan, Y., et al.(2020). Puerarin attenuates diabetic nephropathy by promoting autophagy in podocytes [J]. *Front Physiol*, 11: 73.<http://dx.doi.org/10.3389/fphys.2020.00073>

Li, Y., Ou, S., Liu, Q., Gan, L., Zhang, L., Wang, Y., et al.(2022). Genistein improves mitochondrial function and inflammatory in rats with diabetic nephropathy via inhibiting MAPK/NF-κB pathway [J]. *Acta Cir Bras*, 37: e370601.<http://dx.doi.org/10.1590/acb370601>

Li, Y., Ye, Z., Lai, W., Rao, J., Huang, W., Zhang, X., et al.(2017). Activation of sirtuin 3 by silybin attenuates mitochondrial dysfunction in cisplatin-induced acute kidney injury [J]. *Front Pharmacol*, 8: 178.<http://dx.doi.org/10.3389/fphar.2017.00178>

Liu, H., Wang, Q., Shi, G., Yang, W., Zhang, Y., Chen, W., et al.(2021). Emodin ameliorates renal damage and podocyte injury in a rat model of diabetic nephropathy via regulating AMPK/mTOR-mediated autophagy signaling pathway [J]. *Diabetes Metab Syndr Obes*: 1253-1266.<http://dx.doi.org/10.2147/DMSO.S299375>

Liu, J., Yang, K., Zhou, L., Deng, J., Rong, G., Shi, L., et al.(2024). A new strategy for Astragaloside IV in the treatment of diabetic kidney disease: Analyzing the regulation of ferroptosis and mitochondrial function of renal tubular epithelial cells [J]. *Int Immunopharmacol*, 141: 112794.<http://dx.doi.org/10.1016/j.intimp.2024.112794>

Liu, T., Yang, Q., Zhang, X., Qin, R., Shan, W., Zhang, H., et al.(2020). Quercetin alleviates kidney fibrosis by reducing renal tubular epithelial cell senescence through the SIRT1/PINK1/mitophagy axis [J]. *Life Sci*, 257: 118116.<http://dx.doi.org/10.1016/j.lfs.2020.118116>

Liu, W., Li, F., Guo, D., Du, C., Zhao, S., Li, J., et al.(2023). Schisandrin B Alleviates Renal Tubular Cell Epithelial–Mesenchymal Transition and Mitochondrial Dysfunction by Kielin/Chordin-like Protein Upregulation via Akt Pathway Inactivation and Adenosine 5′-Monophosphate (AMP)-Activated Protein Kinase Pathway Activation in Diabetic Kidney Disease [J]. *Molecules*, 28(23): 7851.<http://dx.doi.org/10.3390/molecules28237851>

Liu, X., Wang, W., Song, G., Wei, X., Zeng, Y., Han, P., et al.(2017). Astragaloside IV ameliorates diabetic nephropathy by modulating the mitochondrial quality control network [J]. *PloS one*, 12(8): e0182558.<http://dx.doi.org/10.1371/journal.pone.0182558>

Liu, X., Zhang, X., Cai, X., Dong, J., Chi, Y., Chi, Z., et al.(2021). Effects of curcumin on high glucose-induced epithelial-to-mesenchymal transition in renal tubular epithelial cells through the TLR4-NF-κB signaling pathway [J]. *Diabetes Metab Syndr Obes*: 929-940.<http://dx.doi.org/10.2147/DMSO.S296990>

Lu, Z., Ji, C., Luo, X., Lan, Y., Han, L., Chen, Y., et al.(2021). Nanoparticle-mediated delivery of emodin via colonic irrigation attenuates renal injury in 5/6 nephrectomized rats [J]. *Front Pharmacol*, 11: 606227.<http://dx.doi.org/10.3389/fphar.2020.606227>

Ma, W., Wang, K.-J., Cheng, C.-S., Yan, G.-q., Lu, W.-L., Ge, J.-F., et al.(2014). Bioactive compounds from Cornus officinalis fruits and their effects on diabetic nephropathy [J]. *J Ethnopharmacol*, 153(3): 840-845.<http://dx.doi.org/10.1016/j.jep.2014.03.051>

Manabe, E., Handa, O., Naito, Y., Mizushima, K., Akagiri, S., Adachi, S., et al.(2008). Astaxanthin protects mesangial cells from hyperglycemia‐induced oxidative signaling [J]. *J Cell Biochem*, 103(6): 1925-1937.<http://dx.doi.org/10.1002/jcb.21583>

Negrette-Guzmán, M., García-Niño, W.R., Tapia, E., Zazueta, C., Huerta-Yepez, S., León-Contreras, J.C., et al.(2015). Curcumin attenuates gentamicin‐induced kidney mitochondrial alterations: possible role of a mitochondrial biogenesis mechanism [J]. *Evid Based Complement Alternat Med*, 2015(1): 917435.<http://dx.doi.org/10.1155/2015/917435>

Pal, P.B., Sinha, K., Sil, P.C.(2014). Mangiferin attenuates diabetic nephropathy by inhibiting oxidative stress mediated signaling cascade, TNFα related and mitochondrial dependent apoptotic pathways in streptozotocin-induced diabetic rats [J]. *PLoS One*, 9(9): e107220.<http://dx.doi.org/10.1371/journal.pone.0107220>

Pérez, A., Mukdsi, J., Valdez, L., Rukavina-Mikusic, I., Díaz de Barboza, G., Tolosa de Talamoni, N., et al.(2023). Naringin prevents diabetic nephropathy in rats through blockage of oxidative stress and attenuation of the mitochondrial dysfunction [J]. *Can J Physiol Pharmacol*, 101(7): 349-360.<http://dx.doi.org/10.1139/cjpp-2022-0449>

Pham, T.K., Nguyen, T.H.T., Yun, H.R., Vasileva, E.A., Mishchenko, N.P., Fedoreyev, S.A., et al.(2023). Echinochrome A prevents diabetic nephropathy by inhibiting the PKC-iota pathway and enhancing renal mitochondrial function in db/db mice [J]. *Mar Drugs*, 21(4): 222.<http://dx.doi.org/10.3390/md21040222>

Qin, X., Jiang, M., Zhao, Y., Gong, J., Su, H., Yuan, F., et al.(2020). Berberine protects against diabetic kidney disease via promoting PGC‐1α‐regulated mitochondrial energy homeostasis [J]. *Brit J Pharmacol*, 177(16): 3646-3661.<http://dx.doi.org/10.1111/bph.14935>

Ren, Q., Cheng, L., Guo, F., Tao, S., Zhang, C., Ma, L., et al.(2021). Fisetin improves hyperuricemia-induced chronic kidney disease via regulating gut microbiota-mediated tryptophan metabolism and aryl hydrocarbon receptor activation [J]. 69(37): 10932-10942.<http://dx.doi.org/10.1021/acs.jafc.1c03449>

Shen, Q., Fang, J., Guo, H., Su, X., Zhu, B., Yao, X., et al.(2023). Astragaloside IV attenuates podocyte apoptosis through ameliorating mitochondrial dysfunction by up-regulated Nrf2-ARE/TFAM signaling in diabetic kidney disease [J]. *Free Radic Biol Med*, 203: 45-57.<http://dx.doi.org/10.1016/j.freeradbiomed.2023.03.022>

Su, J., Gao, C., Xie, L., Fan, Y., Shen, Y., Huang, Q., et al.(2021). Astragaloside II ameliorated podocyte injury and mitochondrial dysfunction in streptozotocin-induced diabetic rats [J]. *Front Pharmacol*, 12: 638422.<http://dx.doi.org/10.3389/fphar.2021.638422>

Sue, Y.-M., Cheng, C.-F., Chang, C.-C., Chou, Y., Chen, C.-H., Juan, S.-H.(2009). Antioxidation and anti-inflammation by haem oxygenase-1 contribute to protection by tetramethylpyrazine against gentamicin-induced apoptosis in murine renal tubular cells [J]. *Nephrology Dialysis Transplantation*, 24(3): 769-777.<http://dx.doi.org/10.1093/ndt/gfn545>

Sun, M.-y., Ye, H.-j., Zheng, C., Jin, Z.-j., Yuan, Y., Weng, H.-b.(2023). Astragalin ameliorates renal injury in diabetic mice by modulating mitochondrial quality control via AMPK-dependent PGC1α pathway [J]. *Acta Pharmacol Sin*, 44(8): 1676-1686.<http://dx.doi.org/10.1038/s41401-023-01064-z>

Tsopmejio, I.S.N., Zhang, J.-t., Wang, Z., Tian, Z.-f., Zhu, H.-y., Zhang, J., et al.(2025). Comparative study of ginsenoside Rg2, 20 (S)-protopanaxatriol, and AFG from ginseng on aging-related kidney injury in SAMP8 mice [J]. *J Ethnopharmacol*, 348: 119807.<http://dx.doi.org/10.1016/j.jep.2025.119807>

Tu, Y., Fang, Q.-J., Sun, W., Liu, B.-H., Liu, Y.-L., Wu, W., et al.(2020). Total flavones of Abelmoschus manihot remodels gut microbiota and inhibits microinflammation in chronic renal failure progression by targeting autophagy-mediated macrophage polarization [J]. *Front Pharmacol*, 11: 566611.<http://dx.doi.org/10.3389/fphar.2020.566611>

Wang, D., Yang, Y., Zou, X., Zheng, Z., Zhang, J.(2020). Curcumin ameliorates CKD-induced mitochondrial dysfunction and oxidative stress through inhibiting GSK-3β activity [J]. *J Nutr Biochem*, 83: 108404.<http://dx.doi.org/10.1016/j.jnutbio.2020.108404>

Wang, H.-Q., Wu, H.-X., Shi, W.-Q., Yang, Y., Lin, M., Wang, K., et al.(2024). Triptolide Attenuates Renal Slit Diagram to Tight Junction Transition in Diabetic Kidney Disease by Regulating Nrf2–Ferroptosis Pathway [J]. *Am J Chin Med*, 52(07): 2161-2185.<http://dx.doi.org/10.1142/S0192415X24500836>

Wang, H., Guan, Y., Karamercan, M.A., Ye, L., Bhatti, T., Becker, L.B., et al.(2015). Resveratrol rescues kidney mitochondrial function following hemorrhagic shock [J]. *Shock Vib*, 44(2): 173-180.<http://dx.doi.org/10.1097/SHK.0000000000000390>

Wang, K., Zheng, X., Pan, Z., Yao, W., Gao, X., Wang, X., et al.(2020). Icariin prevents extracellular matrix accumulation and ameliorates experimental diabetic kidney disease by inhibiting oxidative stress via GPER mediated p62-dependent Keap1 degradation and Nrf2 activation [J]. *Front Cell Dev Biol*, 8: 559.<http://dx.doi.org/10.3389/fcell.2020.00559>

Wang, M., Wang, L.-C., Feng, X.-X., Zhou, Y., Ye, C.-Y., Wang, C.(2022). Icariin improves renal interstitial fibrosis in a rat model of chronic renal failure by regulating mitochondrial dynamics [J]. *Zhongguo Zhong Yao Za Zhi*, 47(8): 2170-2177.<http://dx.doi.org/10.19540/j.cnki.cjcmm.20211104.401>

Wang, Y.-n., Wu, X., Shan, Q.-y., Yang, Q., Yu, X.-y., Yang, J.-h., et al.(2025). Acteoside-containing caffeic acid is bioactive functional group of antifibrotic effect by suppressing inflammation via inhibiting AHR nuclear translocation in chronic kidney disease [J]. *Acta Pharmacol Sin*: 1-14.<http://dx.doi.org/10.1038/s41401-025-01598-4>

Wang, Y., He, X., Xue, M., Sun, W., He, Q., Jin, J.(2023). Germacrone protects renal tubular cells against ferroptotic death and ROS release by re-activating mitophagy in diabetic nephropathy [J]. *Free Radical Res*, 57(6-12): 413-429.<http://dx.doi.org/10.1080/10715762.2023.2277143>

Wang, Y.N., Li, X.J., Wang, W.F., Zou, L., Miao, H., Zhao, Y.Y.(2024). Geniposidic Acid Attenuates Chronic Tubulointerstitial Nephropathy Through Regulation of the NF‐ƙB/Nrf2 Pathway Via Aryl Hydrocarbon Receptor Signaling [J]. *Phytother Res*, 38(11): 5441-5457.<http://dx.doi.org/10.1002/ptr.8324>

Wen, F., Zhang, S., Sun, L., Qian, M., Xu, H.(2023). Salvianolic acid B inhibits oxidative stress in glomerular mesangial cells alleviating diabetic nephropathy by regulating SIRT3/FOXO1 signaling [J]. *Kidney Blood Press Res*, 48(1): 738-751.<http://dx.doi.org/10.1159/000534832>

Wu, D., Yang, X., Zheng, T., Xing, S., Wang, J., Chi, J., et al.(2016). A novel mechanism of action for salidroside to alleviate diabetic albuminuria: effects on albumin transcytosis across glomerular endothelial cells [J]. *Endocrinol Metab*, 310(3): E225-E237.<http://dx.doi.org/10.1152/ajpendo.00391.2015>

Wu, Y., Deng, H., Sun, J., Tang, J., Li, X., Xu, Y.(2023). Poricoic acid A induces mitophagy to ameliorate podocyte injury in diabetic kidney disease via downregulating FUNDC1 [J]. *J Biochem Mol Toxicol*, 37(12): e23503.<http://dx.doi.org/10.1002/jbt.23503>

Xiang, F., Li, X., Hu, W.(2025). Cornuside Ameliorates Diabetic Nephropathy Possibly by Regulating Angiogenesis and MAPK Signaling [J]. *Tohoku J Exp Med*, 266(1): 87-95.<http://dx.doi.org/10.1620/tjem.2024.J112>

Xie, J., Ma, X., Zheng, Y., Mao, N., Ren, S., Fan, J.(2022). Panax notoginseng saponins alleviate damage to the intestinal barrier and regulate levels of intestinal microbes in a rat model of chronic kidney disease [J]. *Ren Fail*, 44(1): 1958-1970.<http://dx.doi.org/10.1080/0886022X.2022.2143378>

Xu, X., Wang, H., Guo, D., Man, X., Liu, J., Li, J., et al.(2021). Curcumin modulates gut microbiota and improves renal function in rats with uric acid nephropathy [J]. *Ren Fail*, 43(1): 1063-1075.<http://dx.doi.org/10.1080/0886022X.2021.1944875>

Xue, H., Li, P., Luo, Y., Wu, C., Liu, Y., Qin, X., et al.(2019). Salidroside stimulates the Sirt1/PGC-1α axis and ameliorates diabetic nephropathy in mice [J]. *Phytomedicine*, 54: 240-247.<http://dx.doi.org/10.1016/j.phymed.2018.10.031>

Xue, M., Cheng, Y., Han, F., Chang, Y., Yang, Y., Li, X., et al.(2018). Triptolide attenuates renal tubular epithelial-mesenchymal transition via the MiR-188-5p-mediated PI3K/AKT pathway in diabetic kidney disease [J]. *Int J Biol Sci*, 14(11): 1545.<http://dx.doi.org/10.7150/ijbs.24032>

Yang, F., Jia, M., Deng, C., Xiao, B., Dai, R., Xiang, Y.(2022). Silibinin ameliorates cisplatin-induced acute kidney injury via activating Nfe2l1-mediated antioxidative response to suppress the ROS/MAPK signaling pathway [J]. *J Mol Histol*, 53(4): 729-740.<http://dx.doi.org/10.1007/s10735-022-10089-3>

Yao, L., Zhao, R., He, S., Feng, Q., Qiao, Y., Wang, P., et al.(2022). Effects of salvianolic acid A and salvianolic acid B in renal interstitial fibrosis via PDGF-C/PDGFR-α signaling pathway [J]. *Phytomedicine*, 106: 154414.<http://dx.doi.org/10.1016/j.phymed.2022.154414>

You, Y., Chen, L., Wu, Y., Wang, M., Lu, H., Zhou, X., et al.(2020). Silibinin promotes cell proliferation through facilitating G1/S transitions by activating drp1-mediated mitochondrial fission in cells [J]. *Cell Transplant*, 29: 0963689720950213.<http://dx.doi.org/10.1016/j.ejmech.2025.117429>

Zhang, B., Zhang, X., Zhang, C., Shen, Q., Sun, G., Sun, X.(2019). Notoginsenoside R1 protects db/db mice against diabetic nephropathy via upregulation of Nrf2-mediated HO-1 expression [J]. *Molecules*, 24(2): 247.<http://dx.doi.org/10.3390/molecules24020247>

Zhang, L., Wang, X., Chang, L., Ren, Y., Sui, M., Fu, Y., et al.(2024). Quercetin improves diabetic kidney disease by inhibiting ferroptosis and regulating the Nrf2 in streptozotocin-induced diabetic rats [J]. *Ren Fail*, 46(1): 2327495.<http://dx.doi.org/10.1080/0886022X.2024.2327495>

Zhang, M., Yang, L., Zhu, M., Yang, B., Yang, Y., Jia, X., et al.(2022). Moutan Cortex polysaccharide ameliorates diabetic kidney disease via modulating gut microbiota dynamically in rats [J]. *Int J Biol Macromol*, 206: 849-860.<http://dx.doi.org/10.1016/j.ijbiomac.2022.03.077>

Zhang, R., Wang, X., Gao, Q., Jiang, H., Zhang, S., Lu, M., et al.(2020). Taurine supplementation reverses diabetes-induced podocytes injury via modulation of the CSE/TRPC6 axis and improvement of mitochondrial function [J]. *Nephron*, 144(2): 84-95.<http://dx.doi.org/10.1159/000503832>

Zhang, T., Chi, Y., Kang, Y., Lu, H., Niu, H., Liu, W., et al.(2019). Resveratrol ameliorates podocyte damage in diabetic mice via SIRT1/PGC‐1α mediated attenuation of mitochondrial oxidative stress [J]. *J Cell Physiol*, 234(4): 5033-5043.<http://dx.doi.org/10.1002/jcp.27306>

Zhang, W., Yuan, W., Xu, N., Li, J., Chang, W.(2017). Icariin improves acute kidney injury and proteinuria in a rat model of pregnancy-induced hypertension [J]. *Mol Med Rep*, 16(5): 7398-7404.<http://dx.doi.org/10.3892/mmr.2017.7513>

Zheng, C.-M., Hou, Y.-C., Tsai, K.-W., Hu, W.-C., Yang, H.-C., Liao, M.-T., et al.(2024). Resveratrol Mitigates Uremic Toxin-Induced Intestinal Barrier Dysfunction in Chronic Kidney Disease by Promoting Mitophagy and Inhibiting Apoptosis Pathways [J]. 21(13): 2437.<http://dx.doi.org/10.7150/ijms.100963>

Zhong, Y., Liu, J., Sun, D., Guo, T., Yao, Y., Xia, X., et al.(2022a). Dioscin relieves diabetic nephropathy via suppressing oxidative stress and apoptosis, and improving mitochondrial quality and quantity control [J]. *Food Funct*, 13(6): 3660-3673.<http://dx.doi.org/10.1039/d1fo02733f>

Zhong, Y., Luo, R., Liu, Q., Zhu, J., Lei, M., Liang, X., et al.(2022b). Jujuboside A ameliorates high fat diet and streptozotocin induced diabetic nephropathy via suppressing oxidative stress, apoptosis, and enhancing autophagy [J]. *Food Chem Toxicol*, 159: 112697.<http://dx.doi.org/10.1016/j.fct.2021.112697>

Zhong, Z., Zhang, Y., Wei, Y., Li, X., Ren, L., Li, Y., et al.(2024). Fucoidan improves early stage diabetic nephropathy via the gut microbiota–mitochondria Axis in high-fat diet-induced diabetic mice [J]. *J Agric Food Chem*, 72(17): 9755-9767.<http://dx.doi.org/10.1021/acs.jafc.3c08503>

Zhu, B., Ni, Y., Gong, Y., Kang, X., Guo, H., Liu, X., et al.(2023). Formononetin ameliorates ferroptosis-associated fibrosis in renal tubular epithelial cells and in mice with chronic kidney disease by suppressing the Smad3/ATF3/SLC7A11 signaling [J]. *Life Sci*, 315: 121331.<http://dx.doi.org/10.3389/fphar.2022.901234>

Zhu, X., Zhang, C., Liu, L., Xu, L., Yao, L.(2024). Senolytic combination of dasatinib and quercetin protects against diabetic kidney disease by activating autophagy to alleviate podocyte dedifferentiation via the Notch pathway [J]. *Int J Mol Med*, 53(3): 26.<http://dx.doi.org/10.3892/ijmm.2024.5350>
